# Unveiling the Photophysical Properties of Indole‐Containing Boron Complexes: Exploring Intramolecular Charge Transfer Character, Tunable Emission, and Large Stokes Shifts

**DOI:** 10.1002/cphc.70386

**Published:** 2026-04-25

**Authors:** Emrah Özcan, Valentyna Kuznetsova, Alina Kaliuzhna, Zehra Coşkun, Mehmet F. Saglam, Ibrahim F. Sengul, Fahri Alkan, Tomas Polivka, Bünyemin Çoşut

**Affiliations:** ^1^ Department of Chemistry Gebze Technical University Gebze Kocaeli Türkiye; ^2^ Department of Physics Faculty of Science University of South Bohemia České Budějovice Czech Republic; ^3^ Department of Chemistry Bilkent University Ankara Türkiye

**Keywords:** BF_2_ complexes, indole, intramolecular charge transfer, large Stokes shift

## Abstract

Embedding boron into indole scaffolds offers promising potential for a diverse range of applications, since both indole and boron‐containing compounds possess remarkable and adjustable chemical, photochemical, and photophysical properties with effortless modifications. In the present study, we showed synthesis and characterization of indolyl‐7‐imines‐based N—N boron complexes. These indole boron platforms were extensively characterized using steady‐state absorption, fluorescence, and ultrafast transient spectroscopy for their photophysical properties and excited‐state dynamics in various solvents. More importantly, our targeted compounds exhibited intramolecular charge transfer (ICT) phenomena, resulting in substantial Stokes shifts and tunable emissions. Theoretical results reported that large Stokes shifts mainly result from the spatial arrangement of HOMO and LUMO energies, with reduced oscillator strengths during the emission process indicating enhanced charge transfer (CT). Notably, tailoring the peripheral phenyl groups allowed the precise control of CT contribution, demonstrating the possibility of manipulating the photophysical properties of indolyl‐imine‐based N—N boron complexes. These findings indicate that this system is one of the most adaptable and tunable among the reported N—N boron complexes‐based molecular systems. The present study critically underscores the potential of N—N boron complexes as a promising building block for applications requiring large Stokes shifts, such as biomedical applications or organic light‐emitting diodes.

## Graphical abstract

Indolyl‐imine–based N—N complexes were synthesized and investigated to clarify how structural modification tunes intramolecular charge transfer (ICT), resulting in large Stokes shifts and tunable emission properties. Steady‐state and ultrafast spectroscopy, supported by density‐functional theory (DFT), showed that altering peripheral phenyl groups allows precise modulation of charge–transfer (CT) behavior.

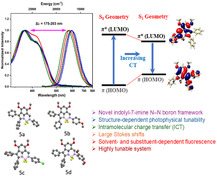

## 1 Introduction

Boron has recently emerged as a key element for organic luminescent materials because of its excellent photophysical properties [1, 2, 3]. Boron‐containing organic compounds have been proven to be versatile platforms for a variety of applications, including organic light‐emitting diodes, field‐effect transistors, photoresponsive materials, photosensitizers, sensors, and imaging materials, with the advantages of environment‐sensitive luminescence, long lifetimes, and high carrier mobility [4, 5, 6, 7, 8]. Delocalization of π‐electrons from organic chelates to the unhybridized p‐orbital of the boron rigidifies boron‐containing organic compounds and thus stabilizes the π‐conjugated systems [9]. The ring‐fused structures lower the energy level of the lowest unoccupied molecular orbital and constrain the conjugated framework, enhancing the radiative emission mechanism. The photophysical properties of such compounds are greatly influenced by both ligand type and nature of the substituted groups on the ligands and/or boron [1]. Over the past few decades, a great number of ligands have been designed and synthesized in order to construct novel boron‐containing platforms. The fluorescence of the reported organoboron systems spans a broad spectral range across the ultraviolet (UV) and visible regions [2, 10, 11, 12].

One well‐known class of boron compounds is boron‐dipyrromethene (BODIPY) derivatives, which stand out for their exceptional fluorescence, robust absorption and emission peaks, and high extinction coefficients [13, 14, 15, 16, 17, 18]. The easily functionalized BODIPY structure allows the creation of various derivatives, offering control over optical properties for a wide range of applications [19]. The BODIPY compounds excel in molecular probes [20], imaging [21], drug delivery [22], organic light‐emitting devices [23], tunable laser dyes [24], light‐harvesting systems [25], and solar cell sensitizers [26]. Despite their excellent photophysical and easily tunable spectroscopic properties, BODIPY derivatives generally exhibit small Stokes shifts, which could lead to self‐absorption [19, 27]. Large Stokes shifts, generally over 80 nm, are preferred for optimal emission efficiency, while BODIPYs typically range from 7 to 15 nm due to their inherent symmetry and conformational rigidity [27, 28, 29, 30]. Major efforts have been devoted to developed new BODIPY analogs with desired properties such as large Stokes shifts, intramolecular charge transfer (ICT), and solvatochromism [19, 31, 32]. As previously shown, the ICT process can lead to a significant increase in the dipole moment of the excited state, resulting in a strong solvatochromic effect and a large Stokes shift [33, 34, 35]. A powerful approach to achieve ICT is the desymmetrization strategy by the expansion of the π‐conjugated system with ligands [19, 36]. Various N, N‐ligands consisting of donor and acceptor sides have been reported in this regard [37, 38, 39]. Another route to achieve ICT property is to synthesize nonpyrrolic BODIPY analogs, which exhibit advantages such as stability; ease of synthesis, particularly for unsymmetrical dyes; larger Stokes shifts; and enhanced solvatochromic effects. It was found from the literature that a wide range of O, O‐bi‐ [40, 41], N, N‐bi‐ [42, 43, 44], N, O‐bi‐ [45, 46], O, N, O‐tri‐ [47, 48], N, N, N‐tri‐ [48], and N, N, O, O‐tetradentated [49, 50] boron complexes have been used to obtain a larger Stokes shift and remarkable photostability.

In that regard, indole is an electron‐rich nitrogen‐containing aromatic heterocyclic compound and one of the most ubiquitous heterocyclic structures found in nature [51, 52]. A variety of coordination systems based on indolyl‐imines have been studied previously [53]. It is well known that Schiff bases can serve as chelating agents, especially when a nearby –NH, –OH, or –SH group is available, allowing the formation of five‐ or six‐membered boron complexes [54, 55]. Indoles, having aldehyde functionality at the C7 position, provide a useful platform to generate indolyl systems through Schiff base reactions, which can be further utilized to produce six‐membered boron complexes. Consequently, indole can be employed as a perfect precursor for the creation of N—N boron complexes. The photophysical properties of indole‐based compounds are dominated by the ICT phenomenon, which provides large Stokes shifts and emissions that can be fine‐tuned by electron‐donor substitution and/or solvent effects [55].

For example, Curiel et al. reported the synthesis of N, N‐chelated 7‐(azaheteroaryl) indole BF_2_ complexes for their luminescent properties and found that large Stokes shift was detected in solution as well as in solid state [56]. In another study, a range of indole‐7‐quinoline boron complexes have been synthesized by Ye et al. for their spectroscopic properties, and the experiments revealed that the targeted indole BF_2_ systems showed an excellent Stokes shift up to 211 nm [57].

**FIGURE 1:**
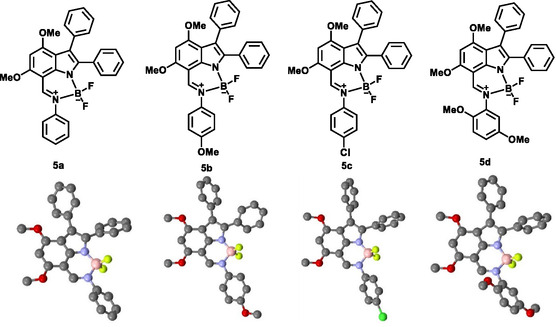
The molecular structures (top) and crystal structures (bottom) of compounds 5a–d.

As part of an ongoing investigation into organoboron analogs, we have successfully synthesized indolyl‐7‐imine‐based N—N boron complexes (Figure 1) featuring both electron donor and acceptor groups. In addition, we present a comprehensive experimental and theoretical analysis of the photophysical properties and excited‐state dynamics of these compounds in various solvents using steady‐state spectra, femtosecond transient absorption spectroscopy, and time‐dependent density functional theory (TDDFT) calculations. The photophysical properties of these compounds are dominated by the ICT phenomenon, which results in large Stokes shifts (175–218 nm) and emissions that can be fine‐tuned by electron‐donor substitution and/or solvent effects.

## 2 Results and Discussion

### 2.1 Synthesis and Structural Characterization

In the current work, 2,3‐diphenyl‐4,6‐dimethoxyindole **1** (Scheme S1) was chosen as the parent scaffold because the unsubstituted indole does not show a propensity to react at position C7. The introduction of electron‐donating methoxy groups located at the C4 and C6 positions of the indole ring directs the electrophilic substitution reaction to the C7 position, forming the basis of the synthetic strategy. The parent molecule 2,3‐diphenyl‐4,6‐dimethoxyindole **1** was prepared by reaction of 3,5‐dimethoxyaniline with benzoin in high yield. The dimethoxyindole **1** was then used as the starting compound to produce indole‐7‐carbaldehyde **2** by the Vilsmeier–Haack reaction employing phosphoryl chloride (POCl_3_) and N, N‐dimethylformamide (DMF). It is well established that the presence of the 2,3‐disubstituted groups and methoxy on the benzenoid ring of the indole ensures that formylation occurs exclusively at the C7 position.

The bidentate indolyl imine ligand precursors **4a‐d** (Scheme S1) were synthesized by the condensation reaction of indole‐7‐carbaldehyde **2** with different commercially available electron‐donating and withdrawing anilines **3a‐d** in ethanol and in the presence of a catalytic amount of glacial acetic acid. Among the indolyl imine ligand systems, compound **4d** was found to be novel, and its structure was confirmed by MALDI‐TOF mass, ^1^H, and ^13^C NMR spectroscopic data (Figures S1–S3). The ^1^H NMR spectrum of compound **4d** showed the replacement of the aldehyde proton at 10.43 ppm by the new imine proton at 9.23 ppm. Four singlets appeared at 3.80, 3.86, 3.90, and 4.01 ppm, which corresponded to the methoxy –(OCH_3_) protons. The ^13^C NMR spectrum showed methoxy –(OCH_3_) carbon peaks at 55.72, 56.30, 56.91, and 57.15 ppm. The molecular ion peak of the **4d** was also determined by MALDI‐TOF mass spectrometry as 493.212 m/z, which is in good agreement with the predicted structure.

The Schiff base reaction provided a straightforward method for obtaining indolyl imine precursors for the preparation of six‐membered BF_2_ complexes. The core of indolyl imines was formed through a Schiff base reaction in which the NH proton of the indole ring was replaced by the BF_2_ moiety. The indolyl‐imine compound **4a** subsequently underwent complexation with boron trifluoride in toluene in the presence of triethylamine to generate the corresponding indole‐based boron complex **5a** with a yield of 65%. Similarly, the compounds **4b‐d** reacted with BF_3_·Et_2_O to produce targeted boron complexes **5b‐d** with high yields. The successful synthesis of target compounds **5a‐d** was confirmed by multiple spectroscopic techniques, including MALDI‐TOF and high‐resolution mass spectrometry (HRMS), ^1^H, ^13^C, ^11^B, and ^19^F NMR, and X‐ray crystallography (Figure S4–S28, Tables S1–S4). In the ^1^H NMR spectra, the characteristic indole NH proton of the starting materials was absent, while the ^13^C, ^11^B, and ^19^F NMR spectra were consistent with the predicted structures. Molecular ion peaks observed in MALDI‐TOF and HRMS matched the calculated values, with only minor deviations. The structures of **5a‐d** were further confirmed by X‐ray crystallography (Figure 1). The asymmetric unit cells of the single crystals and the molecular packing within the crystallographic network are presented in Figure S28, with corresponding crystallographic data provided in Tables S1–S4.

### 2.2 Photophysical Properties

The photophysical properties and solvent dependence of indolyl imine‐based N—N boron complexes (**5a‐d**) containing electron‐donating (–OCH_3_) and withdrawing (–Cl) groups (Figure 1) were measured in solvents with varying polarities at room temperature by steady‐state absorption and fluorescence spectroscopy. The comparative photophysical parameters of the complexes in polar and nonpolar solvents, acetonitrile and toluene, are summarized in Table 1.

**TABLE 1:** Photophysical properties of compounds 5a‐d.

Compounds	Solvents	*λ*_ab_, nm	*λ*_em_, nm	ΔStokes, nm	*τ* a_*F*_, ns	Φ^b^_***F***_
**5a**	Acetonitrile Toluene	376, 416 383, 428	592 578	216 195	T, 1.78 CHISQ, 0.57 T, 3.90 CHISQ, 1.37	0.022 0.106
**5b**	Acetonitrile Toluene	378, 412 386, 420	580 565	202 179	T, 2.59 CHISQ, 0.71 T, 5.12 CHISQ, 1.21	0.054 0.138
**5c**	Acetonitrile Toluene	378, 417 387, 430	596 590	218 203	T, 1.43 CHISQ, 0.84 T, 3.71 CHISQ, 1.26	0.018 0.08
**5d**	Acetonitrile Toluene	373, 412 380, 418	570 555	197 175	T, 2.79 CHISQ, 0.67 T, 5.59 CHISQ, 1.27	0.065 0.142

a Lifetime.

b Fluorescence quantum yield.

**FIGURE 2:**
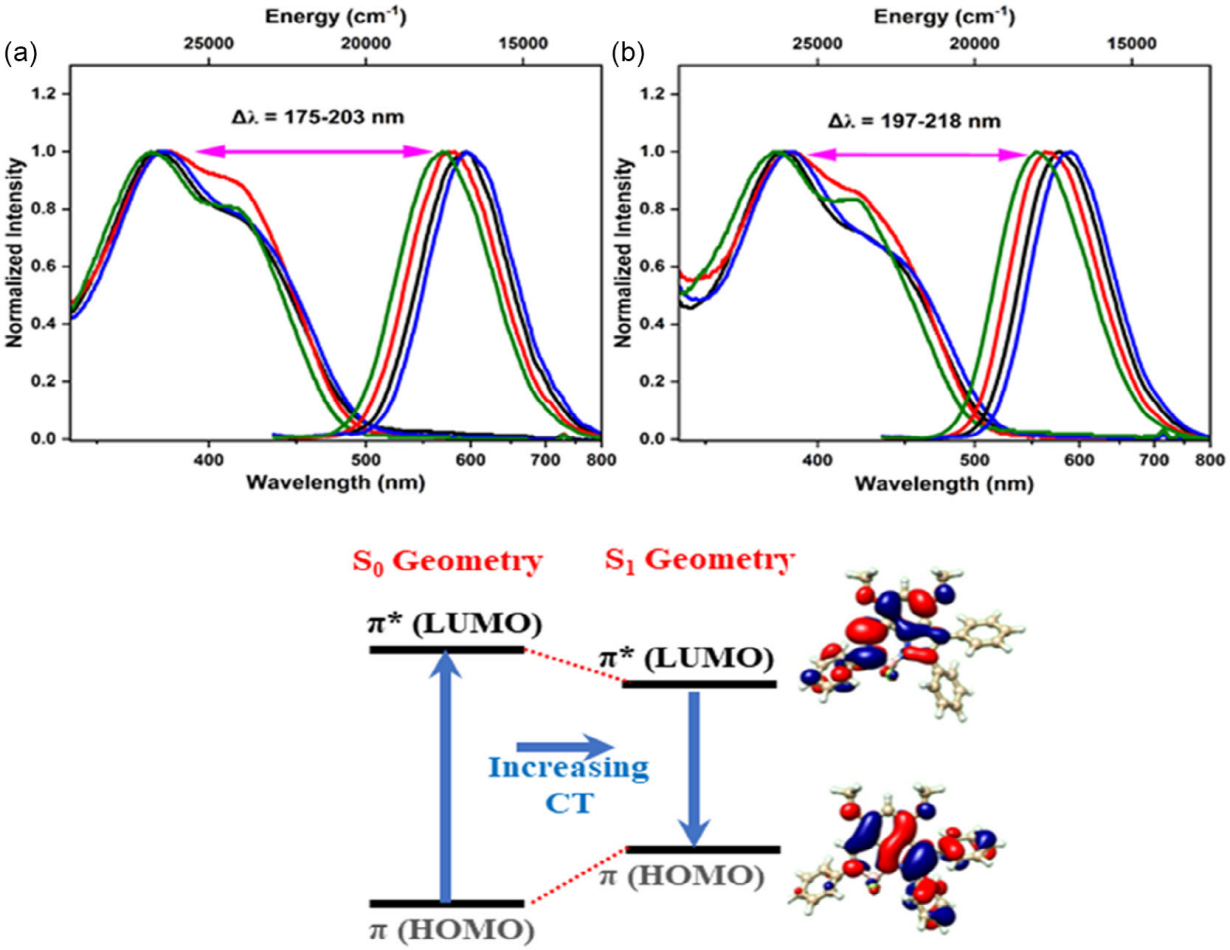
(top) Normalized absorption and fluorescence spectra of compounds 5a‐d (black, red, blue, and green) in (a) acetonitrile and (b) toluene. Fluorescence was excited at 420 nm. (bottom) Example of molecular orbitals and proposed mechanism of generation of the large Stokes shift based on theoretical calculations.

The normalized absorption and emission spectra of the compounds in these solvents are shown in Figure 2a,b, respectively. Figures S29 and S30 present the absorption and emission spectra of these compounds in various solvents with different polarities. Indolyl imine‐based N—N boron complexes showed two distinct absorption peaks. The higher energy transition was observed at around 375 nm, while the lower energy shoulder was around 420 nm.

**FIGURE 3:**
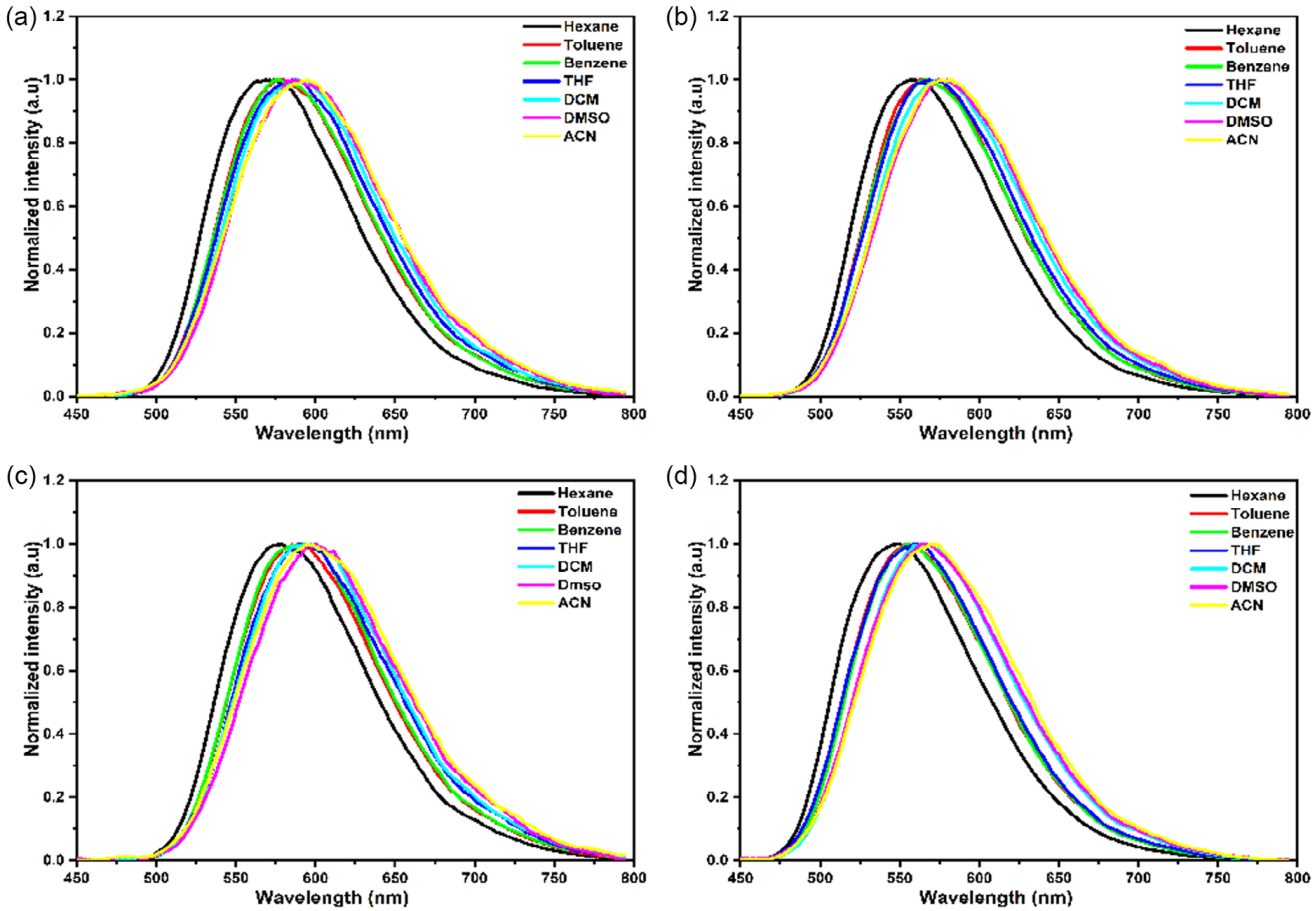
Normalized fluorescence spectra of compounds (a) 5a, (b) 5b, (c) 5c**,** and (d) 5d in solvents with several polarities. Excitation at 420 nm.

The molar absorption coefficients (*ε*) were calculated to be 19.75 × 10^4^, 15.33 × 10^4^, 19.70 × 10^4^, and 13.08 × 10^4^ M^−1^cm^−1^ at the maximum absorption band of the lower energy shoulder for compounds **5a–d**, respectively (Figure S31). The absorption profiles vary slightly in different solvents and exhibit a blue shift (~9 nm) with increasing solvent polarity from toluene to acetonitrile (Figure 2 and Table 1). The small solvatochromic effect and broad absorption peaks of the compounds indicate a possible ICT character, stabilized by solvent polarity, putatively due to a change in the dipole moment in the ground state (Figures 2 and S29) [27, 28, 58]. The compounds exhibit fluorescence emission properties that were recorded using steady‐state and time‐resolved fluorescence spectroscopy techniques. Although the absorption profiles of the compounds demonstrate a slight blue shift with increasing solvent polarity (Figure S29), the corresponding fluorescence spectra exhibit a red shift and quenching with increasing solvent polarity (Figures 3 and S30). Compound **5a**, which contains two electron‐donating methoxy groups (–OCH_3_), exhibits broad emission bands at 592 and 578 nm in acetonitrile and toluene, respectively.

If we compare emission properties of all compounds in the same solvent, compounds **5b** and **5d** show a blueshift compared to **5a** due to the presence of one and two additional electron‐donating methoxy groups at the para‐ and meta‐positions in the peripheral phenyl groups, respectively. On the other hand, compound **5c**, with an electron‐withdrawing chloride group in the para‐position, demonstrates a red shift of emission peak compared to compound **5a** (Figure 2 and Table 1). This behavior originates from a strong ICT character that is increased by the addition of an electron‐withdrawing group at the para position of the peripheral phenyl group [39].

**FIGURE 4:**
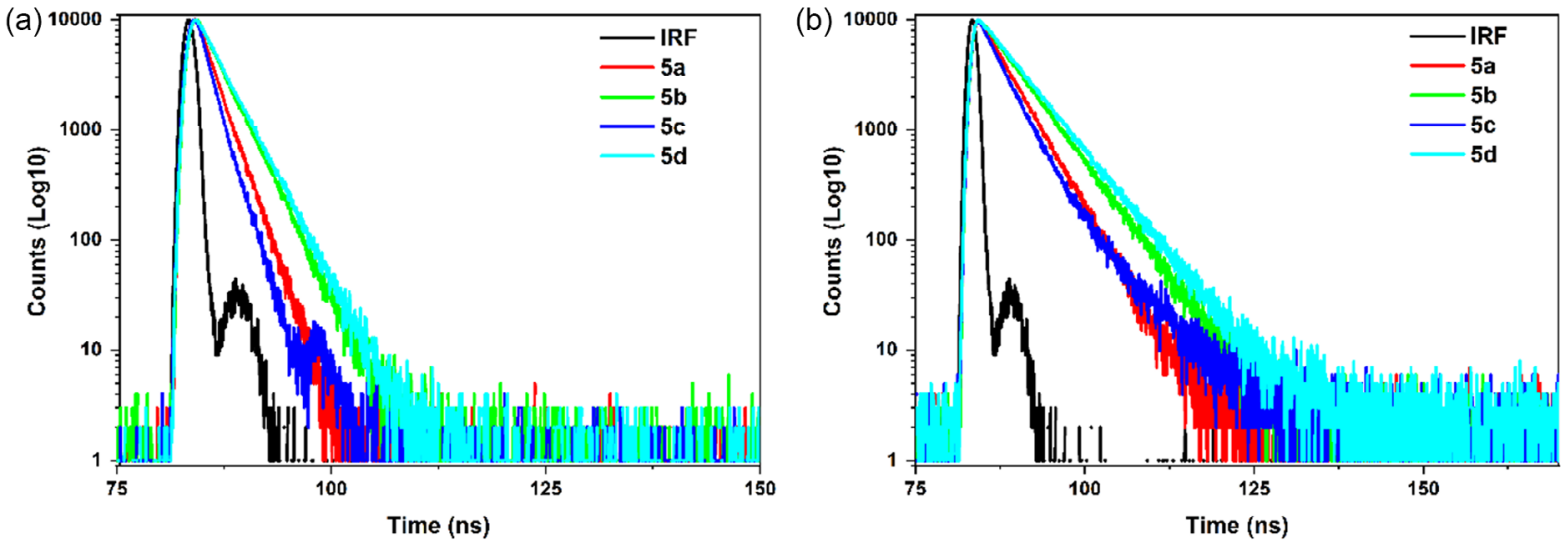
Fluorescence decay profiles of compounds (5a‐d) in a) acetonitrile and b) toluene obtained after excitation at 390 nm.

The results indicate that the excited state has ICT character with a dipole moment larger than that of the ground state, caused by substantial charge redistribution. Due to interaction of a polar solvent and the excited state, a polar solvent makes the ICT state more stable than nonpolar ones [58, 59]. Lippert–Mataga analysis further supports this behavior, showing a linear correlation between the Stokes shift and the solvent orientation polarizability parameter (Δ*f*). The slopes follow the order **5c** > **5a** > **5b** > **5d**, indicating that compound **5c** undergoes the largest change in dipole moment upon excitation and therefore exhibits the strongest ICT character (Figure S32). Despite the presence of the ICT character and large Stokes shifts (vide infra), moderate quantum yields are obtained for compounds **5a**, **5b**, and **5d** in toluene. The quantum yields show a significant decrease in acetonitrile for all systems, indicating an increase in the ICT character (Figure S30 and Table 1). Furthermore, compound **5c** exhibited lower emission and quantum yields (*Φ*_F_ = 0.018) compared to compounds **5a**, **5b,** and **5d** having higher emission and quantum yields (*Φ*_F_ = 0.022, *Φ*_F_ = 0.054, and *Φ*_F_ = 0.065, respectively) in acetonitrile. It is clear that the additional electron‐donating groups, as well as the solvent polarity, increase the quantum yield and emission intensity for the compounds. Furthermore, the fluorescence lifetime values, as a result of low quantum yields, fluorescence intensity, and solvatochromic effect, were determined to be 1.78, 2.59, 1.43, and 2.79 ns in acetonitrile and 3.90, 5.12, 3.71, and 5.59 ns in toluene for compounds **5a** to **5d**, respectively. The lifetimes of all compounds decreased significantly when the solvent polarity increased, and compound **5c** exhibited a shorter lifetime compared to the other compounds, which supports the preceding data (Figures 4, S33, and Table 1).

All compounds exhibit large Stokes shifts ranging from 175 to 218 nm, depending on the solvent polarity. In all cases, the Stokes shift of the compounds shows an increase with increasing solvent polarity, where compound **5c** shows the largest Stokes shift of 218 nm in acetonitrile. Consequently, the broad emission/absorption band, strong solvent dependence of fluorescence emission, along with systematic red shifts and spectral broadening with increasing solvent polarity, align with fluorescence lifetimes and quantum yields. This suggests development of a significant ICT character in excited state, which contributes to the large Stokes shift. Furthermore, the impact of electron‐donating methoxy (–OCH_3_) and electron‐withdrawing chloride (–Cl) groups on indolyl imine‐based N—N boron complexes further influences the photophysical properties in various solvents.

### 2.3 Theoretical Results

To further understand the photophysical properties of investigated systems, density‐functional theory (DFT)/TDDFT computations were performed with B3LYP/6‐311g* level of theory. As shown in Figure S34, ground‐state (*S*_0_) and excited‐state (*S*_1_) geometries of **5a‐d** mainly exhibit six important degrees of freedom: the dihedral angles (*θ*_1_, *θ*_2_, and *θ*_3_) between the peripheral phenyl groups and the central indolyl‐imine moiety, and the bond lengths (C_1_–N_1_, C_4_–C_5_, and C_7_–C_8_) between these groups. Table S5 tabulates these parameters for the *S*_0_ and *S*_1_ geometries of the molecules. For **5a**, **5b**, and **5c**, both S_0_ and S_1_ geometries show similar values for all parameters. In comparison, *θ*_1_ for molecule **5d** is somewhat larger, indicating a more twisted backbone, which is most likely due to the steric effects induced by the –OCH_3_ groups at the phenyl moiety. More importantly, the *S*_1_ geometries for all cases exhibit some degree of planarization with reduced *θ*_1_, *θ*_2_, and *θ*_3_, along with the shortening of bond lengths between phenyl and indolyl‐imine groups, indicating stronger molecular orbital coupling between these moieties upon photoexcitation.

**FIGURE 5:**
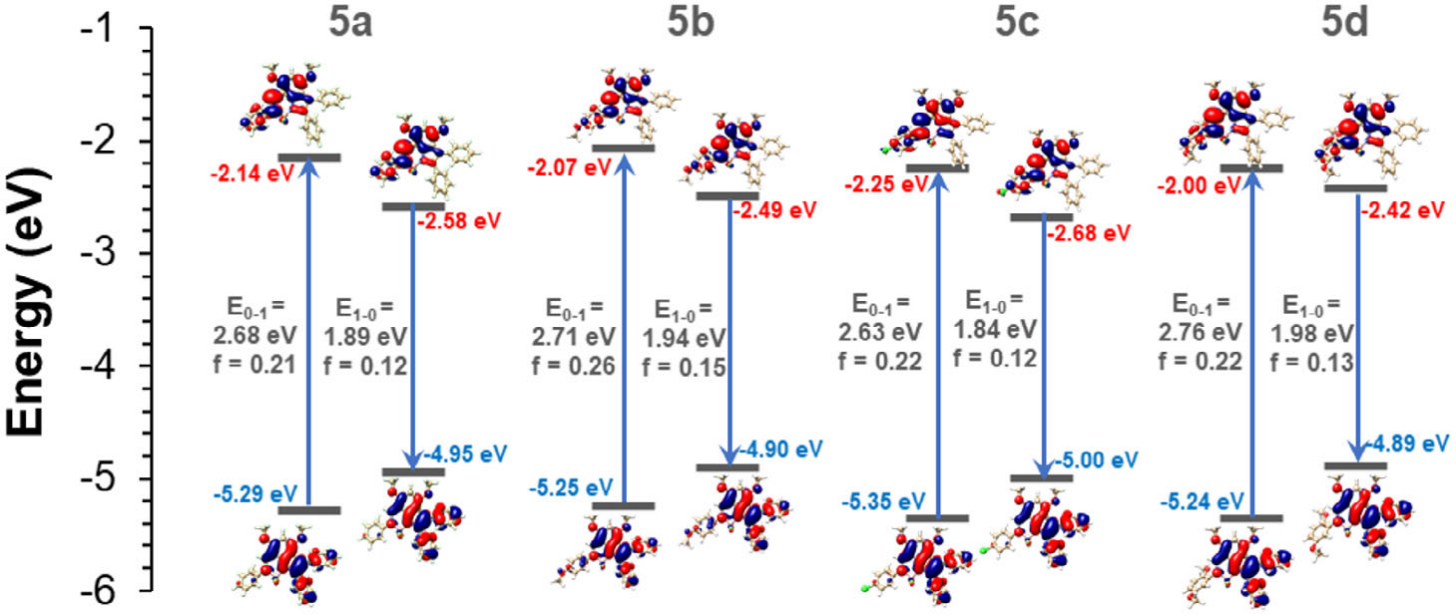
Pictorial representation of absorption (0→1) and emission (1→0) transitions for 5a‐d, along with molecular orbitals, energies (*E*_0→1_ or *E*_1→0_), and oscillator strengths (*f*). Both absorption and emission processes mainly correspond to HOMO–LUMO transitions.

Figure 5 illustrates the correlation between electronic structure and excited states of the investigated systems, whereas Table 2 shows the calculated excited‐state properties for absorption and emission processes. In general, calculated absorption (*λ*_0→1_) and emission (*λ*_1→0_) wavelengths, along with the Stokes shifts (Δ*λ*), show good agreement with the experimental values (Table 1). In all cases, the lowest‐energy absorption (0→1) and emission (1→0) peaks correspond dominantly to the Highest Occupied Molecular Orbital–Lowest Unoccupied Molecular Orbital (HOMO–LUMO) transitions (H→L or L→H). As seen from molecular orbitals (Figure S35), HOMO and LUMO energies exhibit contributions from the π and π* orbitals of a central indolyl‐imine moiety, respectively. For HOMOs, there is a significant contribution from phenyl groups that are bonded to C_4_ and C_7_ positions arising from π‐π interaction between phenyl and indolyl‐imine fragments. In comparison, LUMO energies exhibit contribution from the phenyl group that is attached to the N_1_ position, indicating a π*‐π* interaction between fragments.

**TABLE 2:** Calculated absorption (*λ*_0→1_) and emission (*λ*_1→0_) wavelengths, oscillator strengths (*f*), configurations, and Stokes shifts (Δ*λ*) for 5a‐d.

Compounds	S_0_ geometry			S_1_ geometry			Δ*λ*
	*λ*_0→1_, nm	*f*_0→1_	MO transitions	λ_1→0_, nm	*f*_1→0_	MO transitions	
5a	463	0.21	H→L (0.98)	658	0.12	L→H (0.99)	194
5b	458	0.26	H→L (0.98)	640	0.15	L→H (0.98)	181
5c	472	0.22	H→L (0.98)	673	0.12	L→H (0.98)	201
5d	450	0.22	H→L (0.98)	625	0.13	L→H (0.98)	175

As seen from Figure 5, large Stokes shifts observed for all systems result from both destabilizations of HOMOs and stabilization of LUMOs upon photoexcitation. In the case of HOMO energies, π–π interaction between central indolyl‐imine and phenyl groups is antibonding in nature, and this coupling becomes stronger in the case of the S_1_ geometry because of the reduction of torsion angles (θ_2_ and θ_3_) and shortening bond lengths (C_4_–C_5_ and C_7_–C_8_). In comparison, π*‐π* interaction in LUMOs shows a bonding character between fragments, which stabilizes these levels for the corresponding S_1_ geometries. These results indicate that the spatial arrangements of HOMO and LUMO energies are mainly responsible for the large Stokes shifts of indolyl‐imine‐based N—N boron complexes.

**FIGURE 6:**
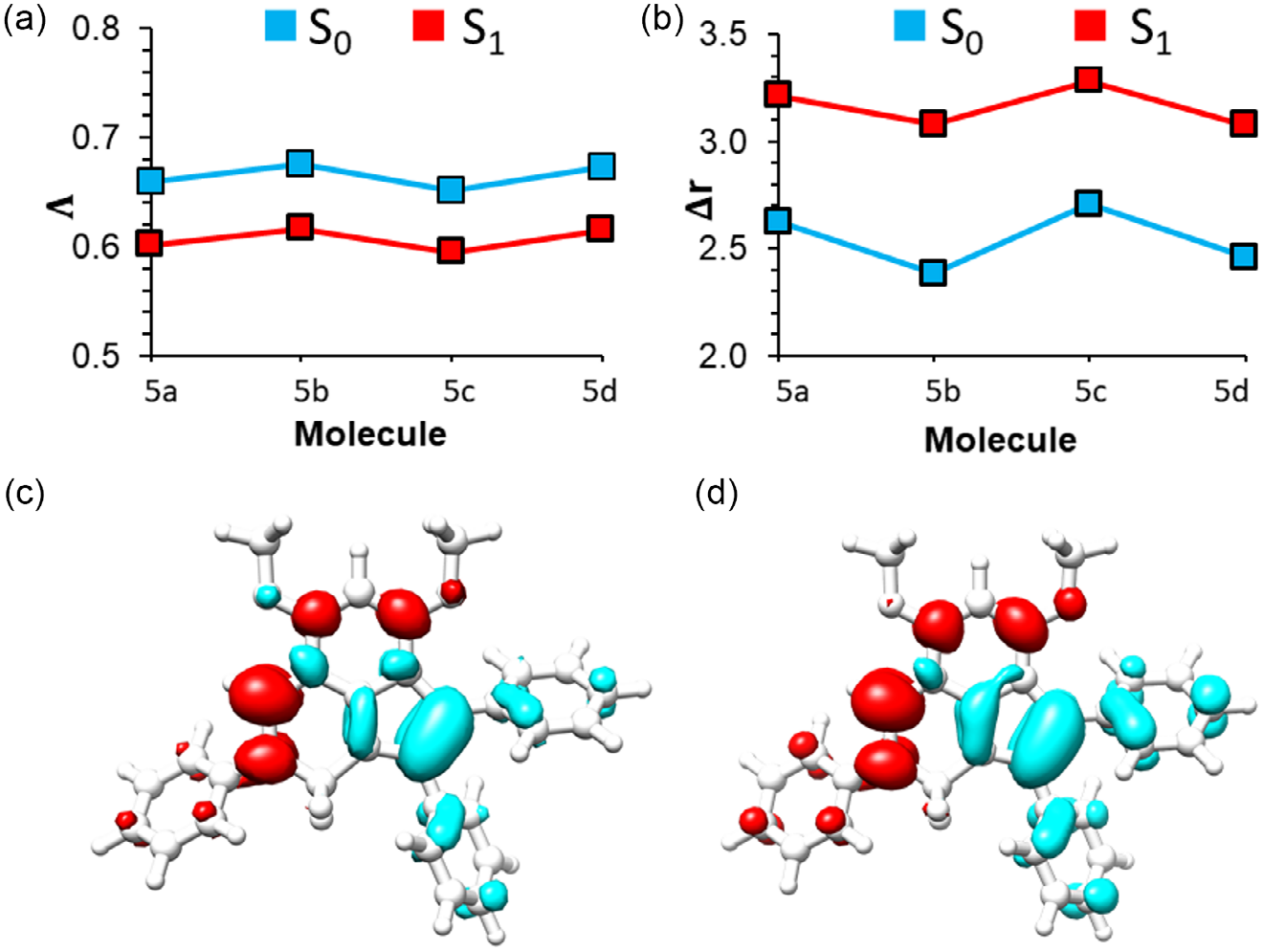
Calculated (a) Λ and (b) Δ*r* parameters for absorption (*S*_0_ geometry) and emission (*S*_1_ geometry) for excited‐state transitions for 5a‐d and excited‐state density differences for 5c for c) 0→1 and d) 1→0 transitions. It is noted that excited‐state density differences are quite similar for the remaining systems as well.

As shown in Table 2, calculated oscillator strengths exhibit a significant decrease for the emission process compared to the case with absorption. It should be noted that this reduction in oscillator strengths can be associated with low fluorescence quantum yields obtained experimentally (Table 1), which mainly arises from increasing ICT character of the emissive state. In order to further understand the ICT mechanism for investigated systems, we have calculated the Λ and Δ*r* (see Computational Methods in supporting information) parameters for absorption (0→1) and emission (1→0) transitions. As shown in Figure 6a, the calculated Λ parameter shows a systematic decrease for 1→0 transitions, indicating a lower degree of overlap for electron and hole wave functions for the emission process compared to the case of absorption. In comparison (Figure 6b), Δ*r* parameters also show a significant increase for ICT character at the S_1_ geometry for 1→0 transitions. We note that the ICT character is calculated to be the largest for **5c** with–Cl substitution at the phenyl group, which also exhibits the lowest quantum yield for emission in the experiment. Meanwhile, ICT character shows a decrease with–OCH_3_ substitutions at the phenyl group, and it is the smallest for **5d,** which also exhibits the highest emission quantum yield experimentally. It is also seen that ICT mainly arises from hole density on phenyl groups attached to the C_4_ and C_7_ positions and electron density on the phenyl group that is attached to the N_1_ position (Figure 6c,d). This ICT character is more enhanced for the 1→0 transitions (S_1_ geometry) as a result of stronger π‐π or π*‐π* interactions between central and peripheral moieties of **5a‐d**. These results indicate that photophysical properties of indolyl‐imine‐based *N–N* boron complexes are strongly correlated with the degree of the ICT character for 1→0 transitions, and it is possible to tune the contribution of charge transfer (CT) with the functionalization of peripheral phenyl groups.

## 3 Ultrafast Transient Absorption Spectroscopy

**FIGURE 7:**
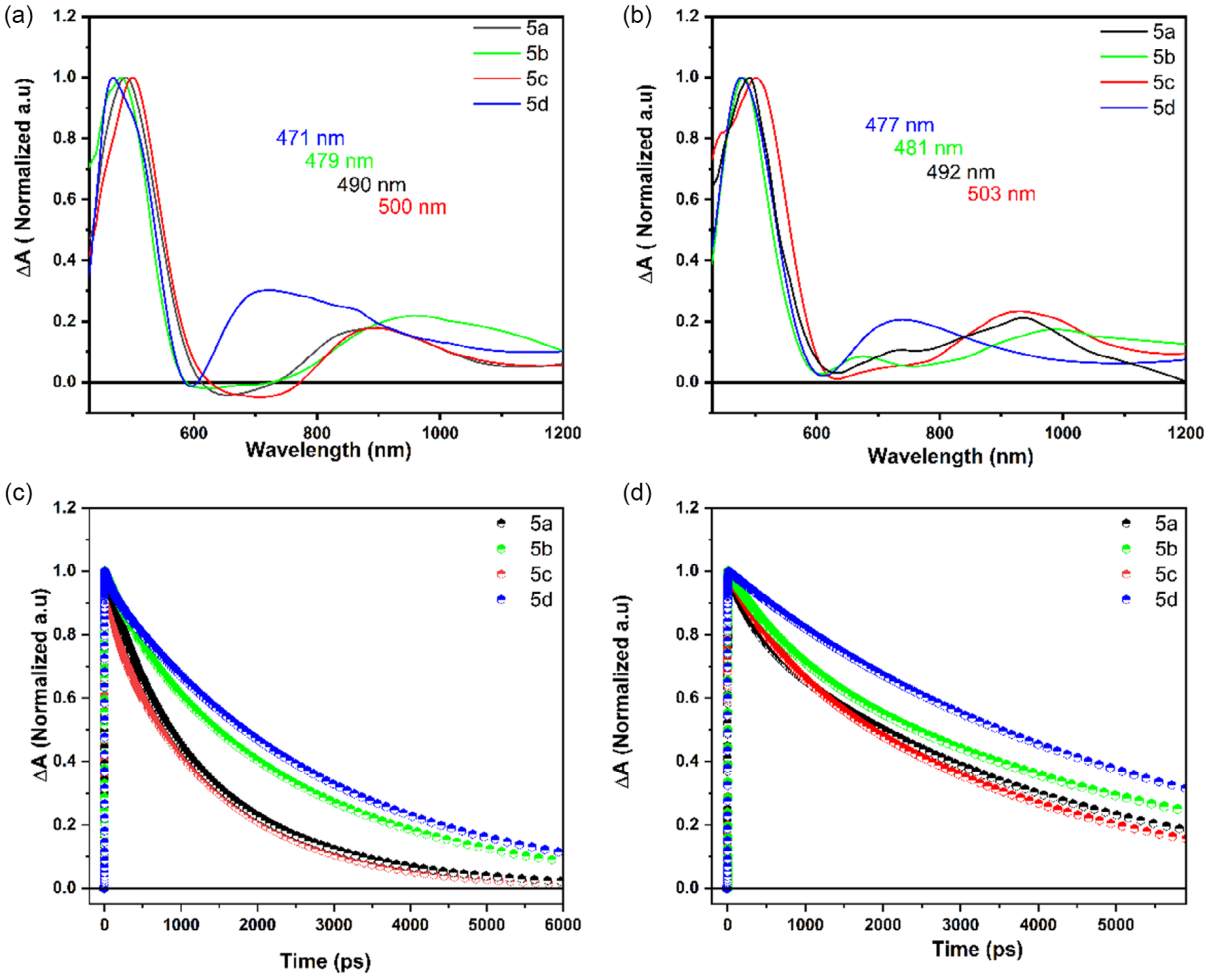
Normalized transient absorption spectra measured at 6 ps delay after excitation at 420 nm of 5a‐d in acetonitrile (a) and toluene (b). The spectra are normalized to the *S*_1_ maximum and smoothed with the Savitzky–Golay method using Origin. The raw data are shown in Figure S36 and S37. Kinetics measured at the maximum of the *S*_1_ *–S*_*n*_ band of 5a‐d in acetonitrile (c) and toluene (d). To better visualize the differences, only normalized fits are shown in the figure, while raw data along with fits are shown in Figure S40 and S41.

Femtosecond transient absorption (TA) spectroscopy was used to understand the dynamic behavior of the excited states of indole‐imine‐based N—N boron complexes **5a‐d** in both polar and nonpolar solvents. In order to eliminate contributions from the higher‐energy vibrational states, the excitation wavelength for each compound was selected to correspond with the lowest vibrational band, and all compounds were excited at 420 nm. Transient absorption spectra follow the excited‐state dynamics by monitoring both positive and negative signals, which include contributions from excited‐state absorption (ESA) and stimulated emission (SE) processes. The ground state bleach (GSB) lies outside the measured spectral window that covers the 450–1200 nm spectral region, as shown in Figure 7a,b. The broad ESA band, due to the *S*_1_–*S*_*n*_ transition, appears between 450 and 600 nm in both polar and nonpolar solvents for all compounds. Apart from minor variations, the overall shape and position of the main transient bands exhibit a similarity for all compounds (Figure S36 and S37). Although the general features observed in the transient absorption spectra are comparable, data recorded for different compounds reveal some changes in energies and dynamics. To visualize these changes, Figure 7a,b compares normalized transient absorption spectra of all compounds measured both in acetonitrile and toluene solutions. The *S*_1_–*S*_*n*_ maximum of all compounds shows a red shift in toluene compared to polar acetonitrile. Compound **5a** peaks at 490 nm, whereas there is a significant blue shift for the compounds **5b** (479 nm) and **5d** (471 nm). This is explained by the presence of one and two additional electron‐donating methoxy groups at the para‐ and meta positions in the peripheral phenyl group, respectively. Conversely, compound **5c** (500 nm), with an electron‐withdrawing chloride group in the para position, demonstrates a red shift compared to compound **5a** (Figure 7a,b). This effect mirrors the shifts observed in absorption spectra (Figure 2a,b).

Besides the dominant ESA bands, the weak negative band in the 600–800 nm spectral region suggests the presence of SE, as these signals align well with the fluorescence spectra of compounds (Figure 2a,b). For some samples, the SE signal appears only as a dip in the strong positive ESA signal caused by the spectral overlap of ESA and SE bands. These SE signals can be ascribed to the ICT signals [60, 61, 62, 63, 64, 65, 66] and display a decreasing trend and a dynamic shift as the solvent polarity decreases from acetonitrile to toluene (Figure 7a,b, and Figure S36 and S37). The amplitude of the SE signal aligns with expected increased ICT character of the emissive excited state (Figures 2–4 and Table 1). The SE band of compound **5c** is localized around 700 nm in acetonitrile, exhibiting a significant red shift and higher magnitude compared to other compounds. This shift and increased magnitude of SE align well with the presence of electron‐donating and electron‐withdrawing groups in the compounds (Figure 7a). The SE band is barely detectable in toluene for all compounds (Figure 7b) due to the nonpolar nature of this solvent. Overall, the results of the ESA and SE signals correlate well with the photophysical and computational results of all compounds, indicating that compound **5c** has an ICT state with largest charge transfer character, while the charge transfer character of the excited state of compound **5d**, which has almost no or very weak SE signal in the 600–800 nm spectral region, is rather small.

Global fitting analysis was employed to estimate the lifetimes of the excited states, and the results are visualized as an evolution‐associated difference spectra (EADS) in both acetonitrile and toluene (Figure S38 and S39). In both acetonitrile and toluene, three‐time components were required to fit the data for all compounds. The first EADS (black), which decays between 300 fs and 3 ps timescale to the red spectrum, is generated upon excitation and could correspond to the initial vibrational relaxation of the excited *S*_1_ state and formation of the state with the ICT character. The second EADS (red) contains the SE signal, indicating that the excited state with the ICT character is generated within the first picosecond. The process associated with the decay of the second EADS has a weak amplitude, as evidenced by only small changes between the second and third EADS. The lifetime of this EADS varies between ~100 ps and ~800 ps and is likely associated with conformational relaxation, which has a much longer lifetime of 3.4 ns/stabilization of the emissive state with ICT character. For compound **5d** in toluene, the second EADS exhibits a longer lifetime of 3.38 ns, which can be attributed to a more twisted backbone, most likely caused by the steric effects of the OCH_3_ groups at the phenyl moiety, as discussed in the theoretical section. The final EADS (blue) is the relaxed *S*_1_ state with ICT character and decays to the ground state within a few nanoseconds. That matches those obtained from fluorescence lifetime experiments. A similar scheme of excited‐state relaxation is observed for all compounds, both in acetonitrile and toluene.

This conclusion is further supported by kinetics taken at prominent (ESA and SE) spectral bands in TA spectra of all compounds in acetonitrile and toluene (Figure 7c,d, and Figure S40 and S41). The kinetics at the maximum of the ESA bands showed a faster decay in acetonitrile compared to toluene for all compounds. The ESA kinetics of compound **5c** decay faster, whereas compound **5d** exhibits slower decay compared to other compounds. These differences are associated with corresponding increases and decreases in the ICT character of the compounds (Figure 7c,d). The obtained results exhibit a notable similarity to the fluorescence lifetimes and oscillator strengths obtained for the compounds (Tables 1 and 2, and Figure 4). Furthermore, kinetics of each compound are compared at the maximum of ESA (black and blue) and SE (red) bands (Figure S40 and S41). The decay kinetics at ~ 490 and ~ 900 nm exhibit similar lifetimes (as seen in Table S6), indicating that the ESA bands at these wavelengths correspond to the same excited‐state process. This suggests that the ESA signal has a broad absorption, covering transitions from the *S*_1_ state and the intramolecular charge transfer (ICT) state. The solvent polarity influences the relative contributions of *S*_1_ and ICT to the overall ESA signal, as demonstrated by the differences between acetonitrile and toluene. In brief, the kinetics observed around ~660–700 nm have very weak amplitude due to the overlap of ESA and SE signals. This spectral overlap complicates the analysis in this region, as the SE dip partially reduces the strong ESA signal.

## 4 Conclusions

In the current study, the synthesis, structural, and photophysical properties of indolyl‐7‐imine‐based N—N boron systems were investigated. Notably, the photophysical characterizations revealed solvent‐dependent ICT characteristics as a predominant feature in those complexes. The photophysical properties of these complexes, with ICT phenomena promoting a large Stokes shift and tunable emission properties, make them excellent candidates for a variety of applications. Their exceptional features have positioned them as promising materials for a wide range of applications, particularly in imaging and organic light‐emitting diodes (OLED) systems.

## 5 Methodology

The details of all characterization methods were given in the Supplementary Information.

### 5.1 Synthesis

The compounds **1–3**, **4a‐c**, and **5a, 5d** (Scheme S1) were synthesized and purified according to the literature [67, 68].

### 5.2 Synthesis of Compound 4d

To a stirred solution of 4,6‐dimethoxy‐2,3‐diphenyl‐indole‐7‐carbaldehyde **2** (100 mg; 0.280 mmol) in ethanol (20 mL), 4‐methoxyaniline **3d** (62 mg; 0.40 mmol) and glacial acetic acid (50 µL) were added. The mixture was stirred under reflux for 2 days. The solvent was removed under reduced pressure, and the crude product was washed with ethanol (50 mL). The resulting orange solid was filtered to give the compound **4d** (Scheme S1; 87 mg; 66%). ^1^H NMR (Figure S1; 500 MHz; CDCl_3_) δ 12.11 (s, 1H, NH), 9.23 (s, 1H, CH=N), 7.45–7.23 (m, 10H, Aryl‐CH), 6.95 (s, 2H, Aryl‐CH), 6.76 (s, 1H, Aryl‐CH), 6.24 (s, 1H, indole‐CH), 4.01 (s, 3H, OCH_3_), 3.90 (s, 3H, OCH_3_), 3.86 (s, 3H, OCH_3_), 3.80 (s, 3H, OCH_3_) ppm; ^13^C NMR (Figure S2; 125 MHz, CDCl_3_) δ 163.32, 161.94, 159.55, 159.12, 154.68, 148.46, 141.83, 137,23, 131.89, 131.74, 128.87, 128.69, 128.10, 127.88, 127.13, 126.62, 126.40, 113.02, 104.78, 102.44, 87.97, 87.33 (Aryl‐C), 57.15, 56.51, 56.30, 55.72 (OCH_3_) ppm; MALDI TOF (m/z) (Figure S3) calc. C_31_H_28_N_2_O_4_ [M]^+^ calculated 492.10, found: 493.21 m/z.

### 5.3 Synthesis of Compound 5a

Triethylamine (1.0 mL) and BF_3_·Et_2_O (1.5 mL) were added to a stirred solution of compound **4a** (90.0 mg; 0.208 mmol) in toluene (35 mL). The reaction mixture was heated to 80°C and stirred for 1 day. The resulting mixture was concentrated under reduced pressure and purified by flash column chromatography (SiO_2_, dichloromethane: hexane; 2:1 v/v) to yield the compound **5a** as an orange powder (Scheme S1; 65 mg, 65%). ^1^H NMR (Figure S4; 500 MHz, CDCl_3_) δ 8.70 (s, 1H, CH = N), 7.52–7.15 (m, 15H, Aryl‐CH), 6.17 (s, 1H, Aryl‐CH), 4.02 (s, 3H, OCH_3_), 3.93 (s, 3H, OCH_3_) ppm; ^13^C NMR (Figure S5; 125 MHz, CDCl_3_) δ 164.80, 162.07, 155.07, 144.61, 139.87, 138.90, 135.20, 133,23, 131.45, 131.41, 129.31, 128.11, 127.62, 127.53, 127.28, 125.86, 125.02, 118.88, 110.80, 98.62, 87.01 (Aryl‐C), 56.44, 55.98 (OCH_3_) ppm; ^11^B NMR (Figure S6; 160 MHz, CDCl_3_) δ 2.03 (t, *J* (B, F)) ppm; ^19^F NMR (Figure S7; 471 MHz, CDCl_3_) δ −122.31 (q, J (B, F)) ppm MALDI TOF (m/z) (Figure S8) calculated for C_29_H_23_BF_2_N_2_O_2_ [M]^+^ 481.18, found: 481.27 [M]^+^, 461.36 [M‐F]^+^ m/z. HRMS measurement (m/z) (Figure S9) calculated for C_29_H_23_BF_2_N_2_O_2_ [M]^+^ 481.1899 and [M‐F]^+^ 461.1837, found: 481.1880 [M]^+^ and 461.1845 [M‐F]^+^. Crystal data and refinement parameters of compound **5a** are given in Table S1.

### 5.4 Synthesis of Compound 5b

Triethylamine (1.0 mL) and BF_3_·Et_2_O (1.5 mL) were added to a stirred solution of compound **4b** (100 mg; 0.216 mmol) in toluene (35 mL). The reaction mixture was heated to 80°C and stirred for 1 day. The resulting mixture was concentrated under reduced pressure and purified by flash column chromatography (SiO_2_, dichloromethane: hexane; 3:1 v/v) to yield the compound **5b** as an orange powder (Scheme S1; 70 mg, 64%). ^1^H NMR (Figure S10; 500 MHz, CDCl_3_) δ 8.67 (s, 1H, CH=N), 7.47–6.91 (m, 14H, Aryl‐CH), 6.16 (s, 1H, Aryl‐CH), 4.01 (s, 3H, OCH_3_), 3.92 (s, 3H, OCH_3_), 3.82 (s, 3H, OCH_3_) ppm; ^13^C NMR (Figure S11; 125 MHz, CDCl_3_) δ 164.36, 161.65, 159.30, 154.61, 139.81, 138.75, 137.64, 135,19, 133.15, 131.38, 131.32, 127.52, 127.41, 127.17, 125.90, 125.72, 118.68, 114.37, 110.72, 98.45, 86.84 (Aryl‐C), 56.31, 55.63 (OCH_3_) ppm; ^11^B NMR (Figure S12; 160 MHz, CDCl_3_) δ 1.99 (t, J (B, F)) ppm; ^19^F NMR (Figure S13; 471 MHz, CDCl_3_) δ −122.73 (q, J (B, F)) ppm. MALDI TOF (m/z) (Figure S14) calculated for C_30_H_25_BF_2_N_2_O_3_ [M]^+^ 510.19, found: 511.19 [M]^+^, 491.19 [M‐F]^+^ m/z. HRMS measurement (m/z) (Figure S15) calculated for C_30_H_25_BF_2_N_2_O_3_ [M+H]^+^ 511.1959 and [M‐F]^+^ 491.1934, found: 511.1988 [M+H]^+^ and 491.1939 [M‐F]^+^.Crystal data and refinement parameters of compound **5b** are given in Table S2.

### 5.5 Synthesis of Compound 5c

Triethylamine (1.0 mL) and BF_3_·Et_2_O (1.5 mL) were added to a stirred solution of compound **4c** (72 mg; 0.154 mmol) in toluene (35 mL). The reaction mixture was heated to 80°C and stirred for 1 day. The resulting mixture was concentrated under reduced pressure and purified by flash column chromatography (SiO_2_, dichloromethane: hexane; 2:1 v/v) to yield the compound **5c** as an orange powder (Scheme S1; 55 mg, 70%). ^1^H NMR (Figure S16); 500 MHz, CDCl_3_) δ 8.66 (s, 1H, CH = N), 7.48–7.14 (m, 14H, Aryl‐CH), 6.16 (s, 1H, Aryl‐CH), 4.03 (s, 3H, OCH_3_), 3.94 (s, 3H, OCH_3_) ppm; ^13^C NMR (Figure S17; 125 MHz, CDCl_3_) δ 165.41, 162.52, 154.91, 143.36, 140.13, 139.15, 135.33, 134,23, 133.36, 131.65, 129.67, 127.89, 127.54, 126.50, 126.15, 119.26, 111.07, 99.03, 87.34 (Aryl‐C), 56.74, 56.25 (OCH_3_) ppm; ^11^B NMR (Figure S18; 160 MHz, CDCl_3_) δ 2.00 (t, J (B, F)) ppm; ^19^F NMR (Figure S19; 471 MHz, CDCl_3_) δ −122.12 (q, J (B, F)) ppm. MALDI TOF (m/z) (Figure S20) calculated for C_29_H_22_BCIF_2_N_2_O_2_ [M]^+^ 515.08, found: 515.09 [M]^+^, 495.29 [M‐F] ^+^ m/z. HRMS measurement (m/z) (Figure. S21) calculated for C_29_H_22_BCIF_2_N_2_O_2_ [M+H]^+^ 515.1509 and [M‐F]^+^ 495.1447, found: 515.1500 [M+H]^+^ and 495.1435 [M‐F]^+^.Crystal data and refinement parameters of compound **5c** are given in Table S3.

### 5.6 Synthesis of Compound 5d

Triethylamine (1.0 mL) and BF_3_·Et_2_O (1.5 mL) were added to a stirred solution of compound **4d** (120 mg; 0.121 mmol) in toluene (35 mL). The reaction mixture was heated to 80°C and stirred for 1 day. The resulting mixture was concentrated under reduced pressure and purified by flash column chromatography (SiO_2_, dichloromethane: hexane; 2:1 v/v) to yield the compound **5d** as a yellow‐orange powder (Scheme S1; 78 mg, 60%). ^1^H NMR (Figure S22; 500 MHz, CDCl_3_) δ 8.66 (s, 1H, CH = N), 7.53–7.01 (m, 10H, Aryl‐CH), 6.99 (s, 1H, Aryl‐CH), 6.87 (s, 1H, Aryl‐CH), 6.19 (s, 1H, Aryl‐CH), 4.02 (s, 3H, OCH_3_), 3.95 (s, 3H, OCH_3_), 3.79 (s, 3H, OCH_3_), 3.78 (s, 3H, OCH_3_) ppm; ^13^C NMR (Figure S23; 125 MHz, CDCl_3_) δ 164.80, 162.23, 158.82, 153.83, 148.12, 140.19, 139.15, 135,60, 133.81, 131.75, 127.86, 127.68, 127.51, 126.03, 118.84, 115.00, 114.76, 113.41, 111.09, 98.31, 87.15, (Aryl‐C), 57.82, 56.64, 56.19, 56.16 (OCH_3_) ppm; ^11^B NMR (Figure S24; 160 MHz, CDCl_3_) δ 1.67 (t, J (B, F)) ppm; ^19^F NMR (Figure S25; 471 MHz, CDCl_3_) δ −123.23 (q, J (B, F)) ppm. MALDI TOF (m/z) (Figure S26) calculated for C_31_H_27_BF_2_N_2_O_4_ [M]^+^ 540.12, found: 541.28 [M]^+^, 521.39 [M‐F]^+^ m/z. HRMS measurement (m/z) (Figure S27) calculated for C_31_H_27_BF_2_N_2_O_4_ [M+H]^+^ 541.2110 and [M‐F]^+^ 521.2048, found: 541.2076 [M+H]^+^ and 521.2054 [M‐F]^+^. Crystal data and refinement parameters of compound **5d** are given in Table S4.

## Supporting Information

Additional supporting information can be found online in the Supporting Information section.

## Conflicts of Interest

The authors declare no conflicts of interest.

## Supporting information

Supplementary Material

## Data Availability

The data that support the findings of this study are available on request from the corresponding author. The data are not publicly available due to privacy or ethical restrictions.
