# Supplementary material for: Unveiling the Photophysical Properties of Indole‐Containing Boron Complexes: Exploring Intramolecular Charge Transfer Character, Tunable Emission, and Large Stokes Shifts
Source: Chemphyschem. 2026 Apr 25;27(8):e70386. doi: 10.1002/cphc.70386 (PMC13110108; doi:10.1002/cphc.70386)
Supplement: Supplementary file 1 — Supplementary Material [file CPHC-27-e70386-s001.pdf]

Unveiling the Photophysical Properties of Indole-Containing Boron Complexes: Exploring Intramolecular Charge Transfer Character, Tunable Emission, and Large Stokes Shifts

Emrah Özcan[a,b*], Valentyna Kuznetsova[b], Alina Kaliuzhna[b], Zehra Çoskun[a], Mehmet F. Saglam[a], Ibrahim F. Sengul[a], Fahri Alkan[c], Tomas Polivka[b], Bünyemin Çoşut[a*]

[a]Department of Chemistry, Gebze Technical University, Gebze, Kocaeli, Türkiye

[b]Department of Physics, Faculty of Science, University of South Bohemia, Branišovská 1760, 37005 České Budějovice

[c]Department of Chemistry, Bilkent University, Ankara, Türkiye

***Corresponding**

Emrah Özcan, PhD

Department of Physics, Faculty of Science, University of South Bohemia, Branišovská 1760, 37005 České Budějovice

ozcane00@prf.jcu.cz

Bunyemin Çoşut, Prof. Dr.

Department of Chemistry, Gebze Technical University, Gebze, Kocaeli, Türkiye

bcosut@gtu.edu.tr

TABLE OF CONTENTS

Scheme S1. Synthesis pathway of compounds **1-5d**....................4

Figure S1: ^{1}H NMR Spectrum of Compound **4** in $CDCl_3$....................9

Figure S2: ^{13}C NMR Spectrum of Compound **4** in $CDCl_3$....................9

Figure S3: Positive ion and linear mode MALDI TOF-MS spectrum of Compound **4**....................10

Figure S4: ^{1}H NMR Spectrum of Compound **5a** in $CDCl_3$....................10

Figure S5: ^{13}C NMR Spectrum of Compound **5a** in $CDCl_3$.....................11

Figure S6: ^{11}B NMR Spectrum of compound **5a** in $CDCl_3$.....................11

Figure S7: ^{19}F NMR Spectrum of compound **5a** in $CDCl_3$.....................12

Figure S8: Positive ion and linear mode MALDI TOF-MS spectrum of Compound **5a**....................13

Figure S9: HRMS spectrum of Compound **5a**....................13

Table S1: Crystal data and refinement parameters for compounds **5a**.....................14

Figure S10: ^{1}H NMR Spectrum of Compound **5b** in $CDCl_3$....................15

Figure S11: ^{13}C NMR Spectrum of Compound **5b** in $CDCl_3$....................15

Figure S12: ^{11}B NMR Spectrum of compound **5b** in $CDCl_3$.....................16

Figure S13: ^{19}F NMR Spectrum of compound **5b** in $CDCl_3$.....................16

Figure S14: Positive ion and linear mode MALDI TOF-MS spectrum of Compound **5b**....................17

Figure S15: HRMS spectrum of Compound **5b**....................17

Table S2: Crystal data and refinement parameters for Compound **5b**.....................14

Figure S16: ^{1}H NMR Spectrum of Compound **5c** in $CDCl_3$....................19

Figure S17: ^{13}C NMR Spectrum of Compound **5c** in $CDCl_3$.....................19

Figure S18: ^{11}B NMR Spectrum of Compound **5c** in $CDCl_3$.....................20

Figure S19: ^{19}F NMR Spectrum of Compound **5c** in $CDCl_3$.....................20

Figure S20: Positive ion and linear mode MALDI TOF-MS spectrum of Compound **5c**....................21

Figure S21: HRMS spectrum of Compound **5c**....................21

Table S3: Crystal data and refinement parameters for Compound **5c**.....................22

Figure S22: ^{1}H NMR Spectrum of Compound **5d** in $CDCl_3$....................23

Figure S23: ^{13}C NMR Spectrum of Compound **5d** in $CDCl_3$.....................23

Figure S24: ^{11}B NMR Spectrum of Compound **5d** in $CDCl_3$.....................24

Figure S25: ^{19}F NMR Spectrum of Compound **5d** in $CDCl_3$.....................24

Figure S26: Positive ion and linear mode MALDI TOF-MS spectrum of Compound **5d**....................25

Figure S27: HRMS spectrum of Compound **5d**....................25

Table S4: Crystal data and refinement parameters for Compound **5d**. ...26

Figure S28: Asymmetric unit cell (left) for the single-crystal of 5a-d and molecular packing (right) along the crystallographic network. ...27

Figure S29: Steady-state absorption spectra of compounds a) **5a**, b) **5b**, c) **5c** and d) **5d** in solvent with several polarities. Color coding is the same as in all panels. ($1x10^{-5}$ M)...28

Figure S30: Steady-state fluorescence emission spectra of compounds a) **5a**, b) **5b**, c) **5c** and d) 5d in solvent with several polarities. Color coding is the same as in all panels. ($1x10^{-5}$ M; Ex: 420 nm)...29

Figure S31: Absorption spectra of compounds a) 5a, b) 5b, c) 5c and d) 5d in chloroform at different concentrations: $8x10^{-6}$ M, $6x10^{-6}$ M, $4x10^{-6}$ M, $2x10^{-6}$ M.)...30

Figure S32: Plot of Stokes shift versus solvent orientation polarizability for compounds 5a–d...31

Figure S33: The fluorescence decay profiles of compounds a) 5a, b) 5b, c) 5c and d) 5d in solvent with several polarities by excitation 390 nm. Color coding is the same as in all panels.)...32

Figure S34: Geometrical parameters for the investigated indolyl-imine-based N-N boron complexes. ...33

Table S5: Calculated geometrical parameters for **5a-d**. ...33

Figure S35: HOMO and LUMO levels for **5a-d** in S_0 geometries...34

Figure S36: Transient absorption spectra of **5a-d** in acetonitrile (Acn) after excitation at 420 nm. The transient absorption spectra were measured at the time delays indicated in the panels ...35

Figure S37: Transient absorption spectra of **5a-d** in toluene (Tol) after excitation at 420 nm. The transient absorption spectra were measured at the time delays indicated in the panels. ...36

Figure S38: EADS data obtained from global fitting of **5a-d** in acetonitrile. ...37

Figure S39: EADS data obtained from global fitting of **5a-d** in toluene...37

Figure S40: Comparison of the kinetics at the excited state absorption (S_1 maximum, black), stimulated emission (red), and NIR spectral region (blue) of compounds **5a-d** in acetonitrile. The probing wavelengths for each molecule are shown individually in the graphs after excitation at 420 nm. The solids lines represent fits obtained from global fitting analysis.... ...38

Figure S41: Comparison of the kinetics at the excited state absorption (S_1 maximum, black), and NIR spectral region (blue and red) of compounds **5a-d** in toluene. The probing wavelengths for each molecule are shown individually in the graphs after excitation at 420 nm. The solids lines represent fits obtained from global fitting analysis...39

Table S6. Fitting parameters of compounds kinetics measured in different solvents. 40

References 41

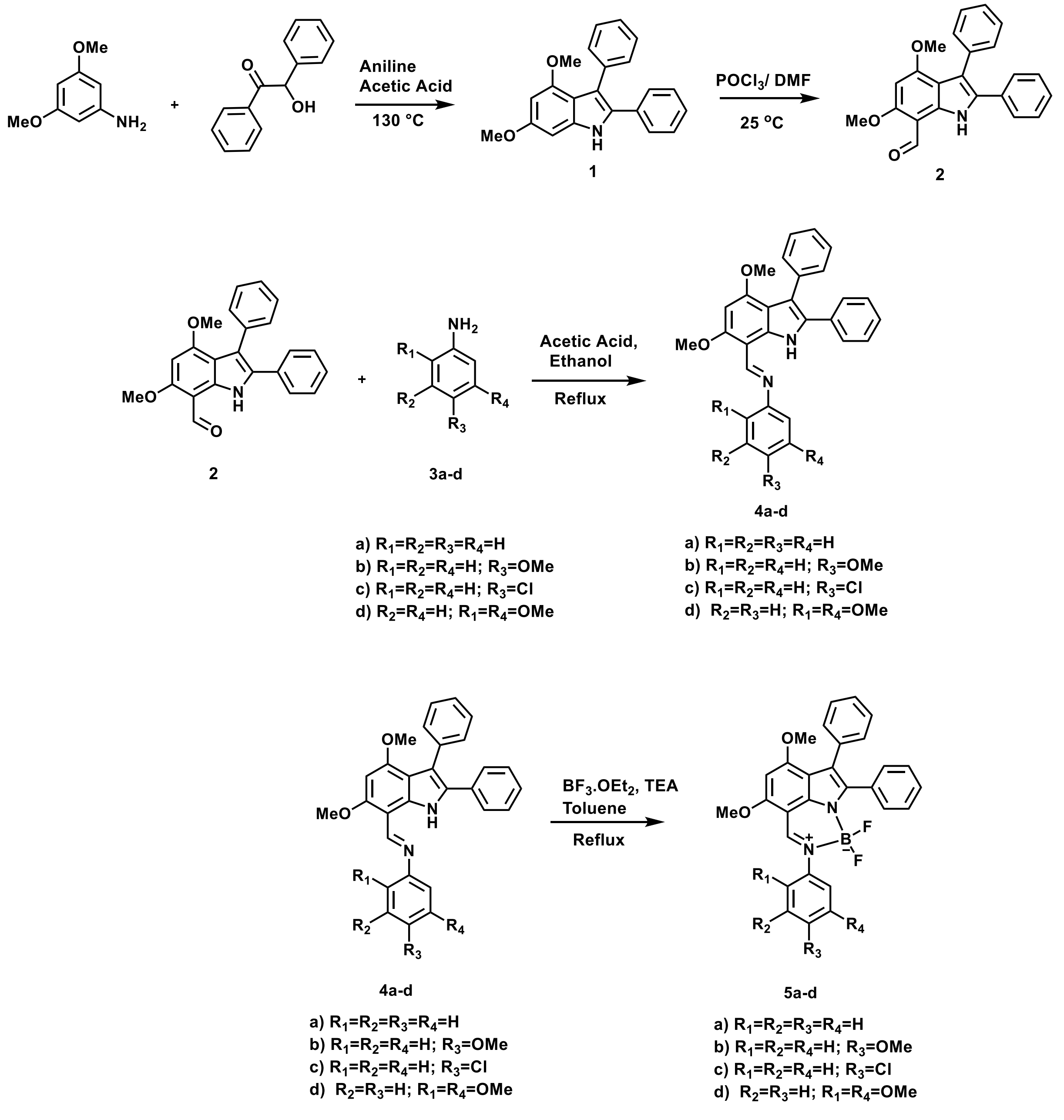

Scheme S1. Synthesis pathway of compounds 1-5d.

Materials and Methods

All reagents used without further purification were purchased from Sigma Aldrich (USA) and all solvents were obtained from Merck (USA). Reactions were monitored by thin layer chromatography using Merck TLC Silica gel 60 F254. Silica gel column chromatography was performed over Merck Silica gel 60 (particle size: 0.040-0.063 mm, 230-400 mesh ASTM).

Chemical characterization

MALDI-TOF mass spectra were acquired in linear modes with average of 50 shots on a Bruker Daltonics Microflex mass spectrometer equipped with a nitrogen UV-Laser operating at 337 nm. HRMS spectra of the synthesized compounds were taken from their solutions in methanol using positive ion electrospray ionization (ESI+) techniques with a Waters LCT Premier XE UPLC/MSTOF system by MassLynx 4.1 software. An Aquity BEH C18 column (2.1100 × 100 mm, 1.7 µM, flow rate 0.3 mL/min) as the stationary phase, CH3CN: H2O (1–90%) gradient solvent system containing formic acid (0.1%) as the mobile phase was used. 1H, 13C, 11B, and 19F NMR spectra were recorded for all compounds in CDCl3 by a Varian INOVA 500 MHz spectrometer. Electronic absorption spectra in the UV-Vis region were recorded with a Shimadzu 2101 UV-Vis spectrophotometer. Fluorescence excitation and emission spectra were recorded on a Varian Eclipse spectrofluorometer using 1 cm path length cuvettes at room temperature. The fluorescence lifetimes were obtained using Horiba- Jobin-Yvon-SPEX Fluorolog 3-2iHR instrument with Fluoro Hub-B Single Photon Counting Controller at an excitation wavelength of 360 nm for all compounds. Signal acquisition was performed using a TCSPC module. The steady-state absorption, fluorescence emission, and fluorescence lifetime measurements were performed at the same concentration (1×10^{-5} M).

The parameters for fluorescence quantum yields

The fluorescence quantum yields were determined in acetonitrile and toluene using the fluorescence of quinine sulfate as a standard. Fluorescence quantum yields (ΦF) were calculated by the comparative method (Eq. 1) [1].

$$\Phi_F = \Phi_F(Std)\,\frac{F \,.\, A_{Std} \,.\, n^2}{F_{Std} \,.\, A \,.\, n_{Std}^2} \qquad \text{(Eq.1)}$$

where ΦF (Std) is the fluorescence quantum yield of standard. Quinine sulfate (ΦF = 0.54 in water) was used as a standard [2]. F and F_{Std} are the areas under the fluorescence emission curve of samples (**5a-d**) and the standard, respectively. A and A_{Std} are the respective absorbance of the samples and standard at the excitation wavelengths, respectively. In addition, n and n_{std} are the refractive indices of solvents used for the sample and standard, respectively.

Lippert–Mataga analysis

The solvent polarity dependence of the photophysical properties of compounds **5a–d** was analyzed using the Lippert–Mataga equation (FigS32), which relates the Stokes shift (Δν) to the solvent orientation polarizability (Δf) [3]. The Stokes shift was calculated from the difference between the absorption and emission maxima according to:

$$\Delta\nu = \nu_{abs} - \nu_{em}$$

where ν_{abs} and ν_{em} represent the absorption and emission maxima expressed in cm^{-1}. The solvent orientation polarizability (Δf) was calculated using:

$$\Delta f = (\varepsilon - 1)/(2\varepsilon + 1) - (n^2 - 1)/(2n^2 + 1)$$

where ε is the dielectric constant and n is the refractive index of the solvent. The absorption and emission maxima of compounds **5a–d** were measured in hexane, toluene, dichloromethane, dimethyl sulfoxide, and acetonitrile. The Stokes shifts were plotted as a function of Δf, and linear fitting was performed using OriginPro software to determine the slopes of the correlations.

X-ray data collection and structure refinement

Unit cell measurements and intensity data collection was performed on an Bruker APEX II QUAZAR three-circle diffractometer using monochromatized Mo *Kα* X-radiation (λ = 0.71073 Å). Indexing was performed using APEX2 [4]. Data integration and reduction were carried out with SAINT V8.34A [5]. Absorption correction was performed by multi-scan method implemented in SADABS V2014/5 [6]. The structures were solved and refined using the Bruker SHELXTL Software Package [7]. All non-hydrogen atoms were refined anisotropically using all reflections with $I > 2\sigma(I)$. The C-bound H atoms were positioned geometrically and refined

using a riding mode. The N-bound H atoms were located from the difference Fourier map and and restrained to be 0.89 Å from N atom using DFIX and their position were constrained to refine on their parent N atoms with Uiso(H) = 1.2Ueq(N). Furthermore, the unit cells of all compounds (**5a-d**) had a disordered solvent molecule which couldn't be modelled. Therefore, the SQUEEZE command of PLATON [8] was used for removing solvent molecules and then the rest of the molecules were refined without the solvent. The final geometrical calculations and the molecular drawings were carried out with Platon (version 1.17) and Mercury CSD (version 3.5.1) program [8,9]. CIFs were deposited with the Cambridge Crystallographic Data Centre (CCDC 2538069 for 5a, CCDC 2469750 for 5b, CCDC 2538083 for 5c, CCDC 2538101 for 5d)

Computational Methods

All DFT and TDDFT computations were performed with ORCA v5.03 [10] program package using B3LYP [11,12] functional and 6-311g* basis set. Both ground state (S0) and excited-state (S1) geometries were optimized without any constraints. Solvent effects were considered with toluene as solvent within the polarizable continuum formalism [13]. To quantify the degree of CT on excited states, Λ [14] and Δr [15] parameters were computed using Multiwfn [16] program. Herein, Λ denotes the overlap between electron and hole wave functions for excited states, which ranges between 0 (full CT) and 1 (no CT). In comparison, Δr computes the spatial extent of CT for an excited state transition. Visualization of molecular orbitals and excited-state density differences were performed with USFC Chimera [17].

Transient absorption spectroscopy.

Transient absorption measurements were performed by a modular laser system consisting of a Ti:sapphire regenerative amplifier (Spitfire Ace-100F, Spectra-Physics, USA) implemented with a Ti:sapphire oscillator (MaiTai SP, Spectra-Physics, USA), and pumped by an Nd:YLF laser (Empower 30, Spectra-Physics, USA). The laser system generates ~100 fs pulses centered at 800 nm at a repetition rate of 1 kHz, and the output is separated by a beam splitter into two beams. The former goes through an optical parametric amplifier (TOPAS Prime, Light Conversion, Lithuania) to generate an excitation beam. The latter is used to generate a 1250 nm seed beam in a home-built nonlinear optical parametric amplifier. The seed beam is focused on a continuously moving 3 mm CaF_2 plate to generate a white-light continuum in the 450–1200 nm spectral region. The white-light beam was collimated by an off-axis

parabolic mirror to minimize chirp and chromatic aberration and split into reference and probe beams by a broadband 50/50 beam splitter. The probe beam was focused by a 250 mm spherical mirror to the sample where it overlapped with the excitation beam. Reference and probe beams were scattered in a prism spectrograph (Pascher Instruments, Sweden) and detected by a double CCD array that allowed to measure in the whole spectral region. Before each experiment the spectrometer was calibrated by placing a multiple oxide filter (WCT 2065, Avian Technologies, USA) into the white light beams in front of an entrance slit. The pump polarization was adjusted at the magic angle (54.7) with regard to the probe. The pump photon density at the sample was kept below 10^{14} photons.cm^{-2}.pulse^{-1}.

Data Analysis.

A global fitting software was used to analyze the resulting spectro-temporal data sets (CarpetView, Light Conversion, Lithuania). To visualize the excited-state dynamics, we assumed that the excited system evolves sequentially and irreversibly. Each component in the sequential scheme represents individual excited-state species, and the spectral profile of each species is called the evolution-associated difference spectrum (EADS). The spectra were chirp-corrected using the same software's correction routine.

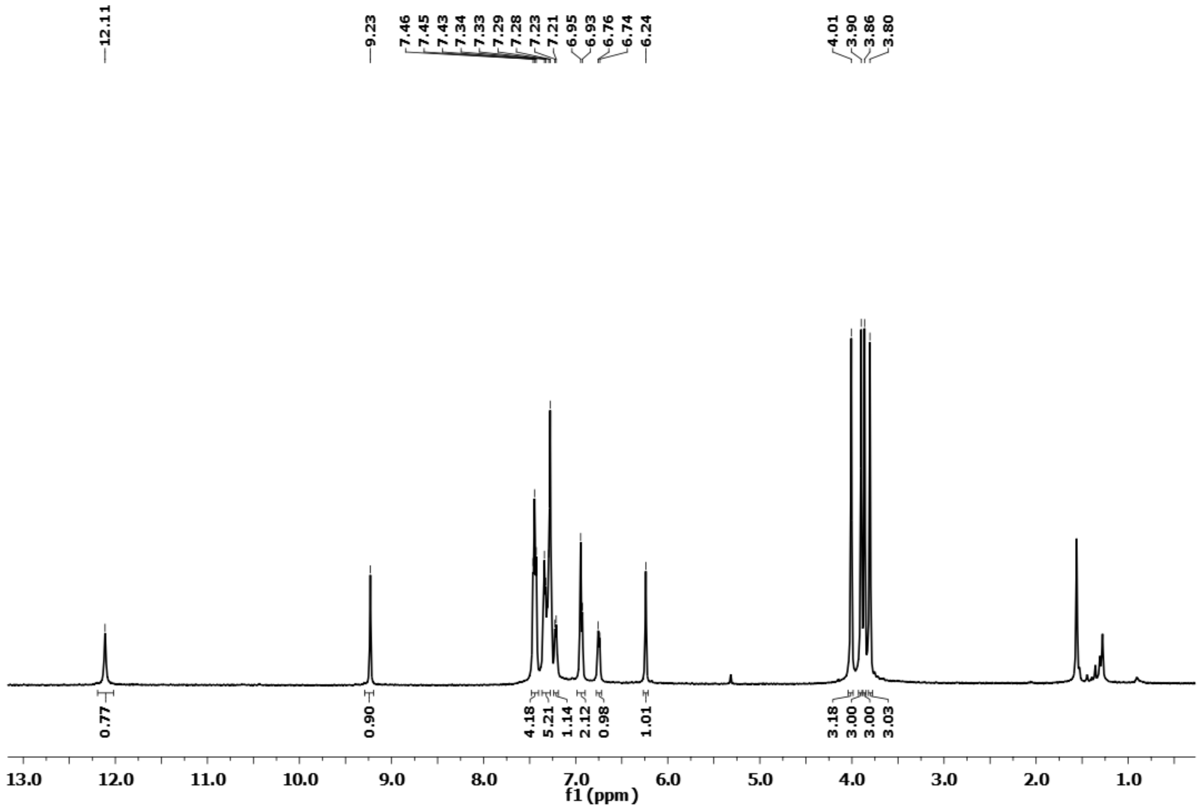

Figure S1. ^{1}H NMR Spectrum of compound 4d.

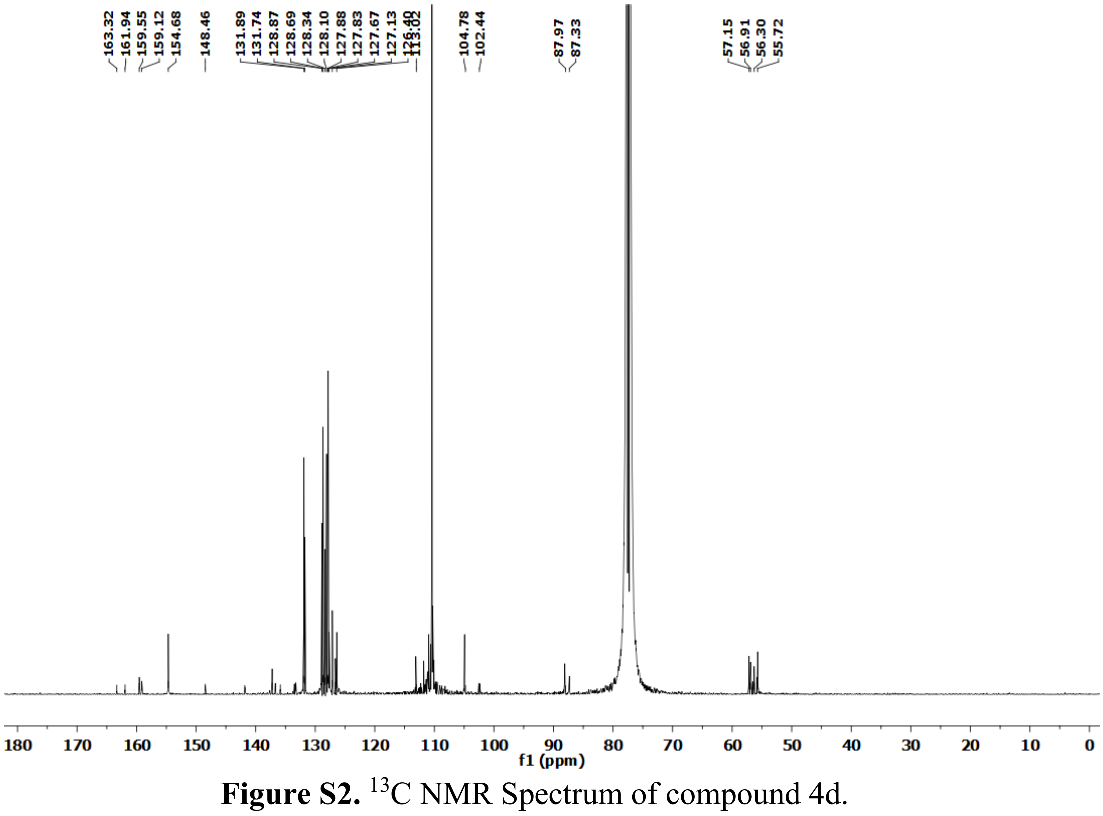

Figure S2. ^{13}C NMR Spectrum of compound 4d.

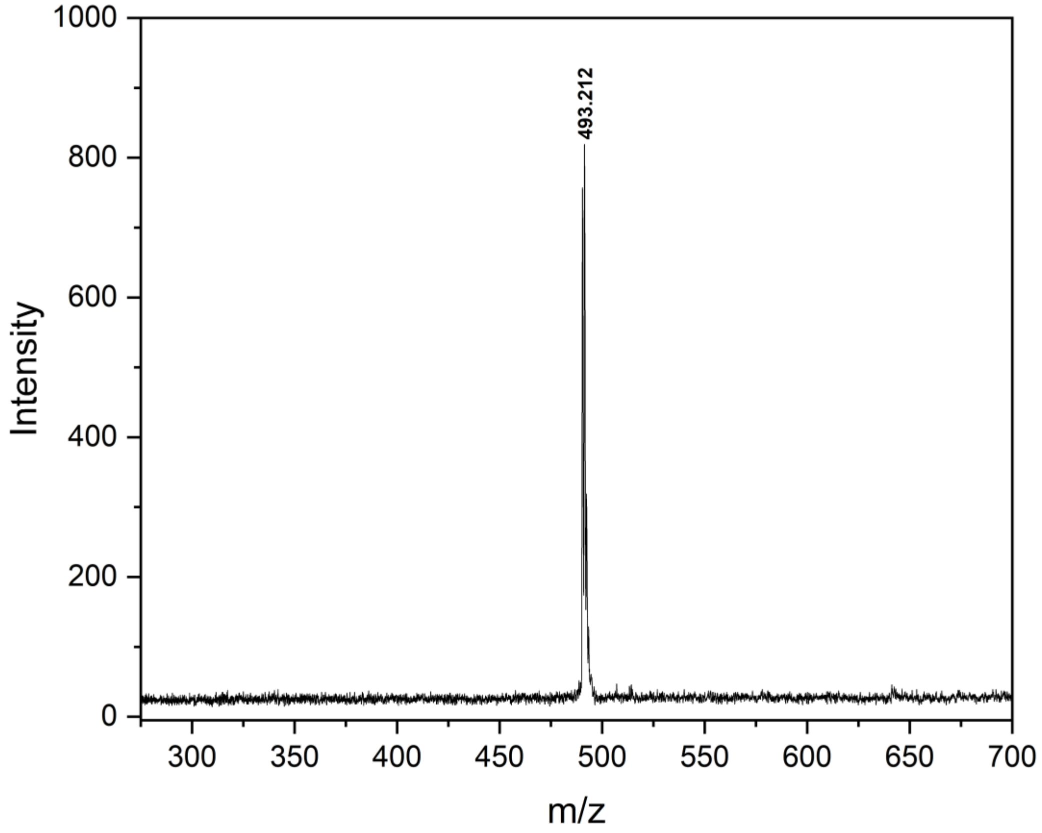

Figure S3. Positive ion and linear mode MALDI TOF-MS spectrum of compound 4d.

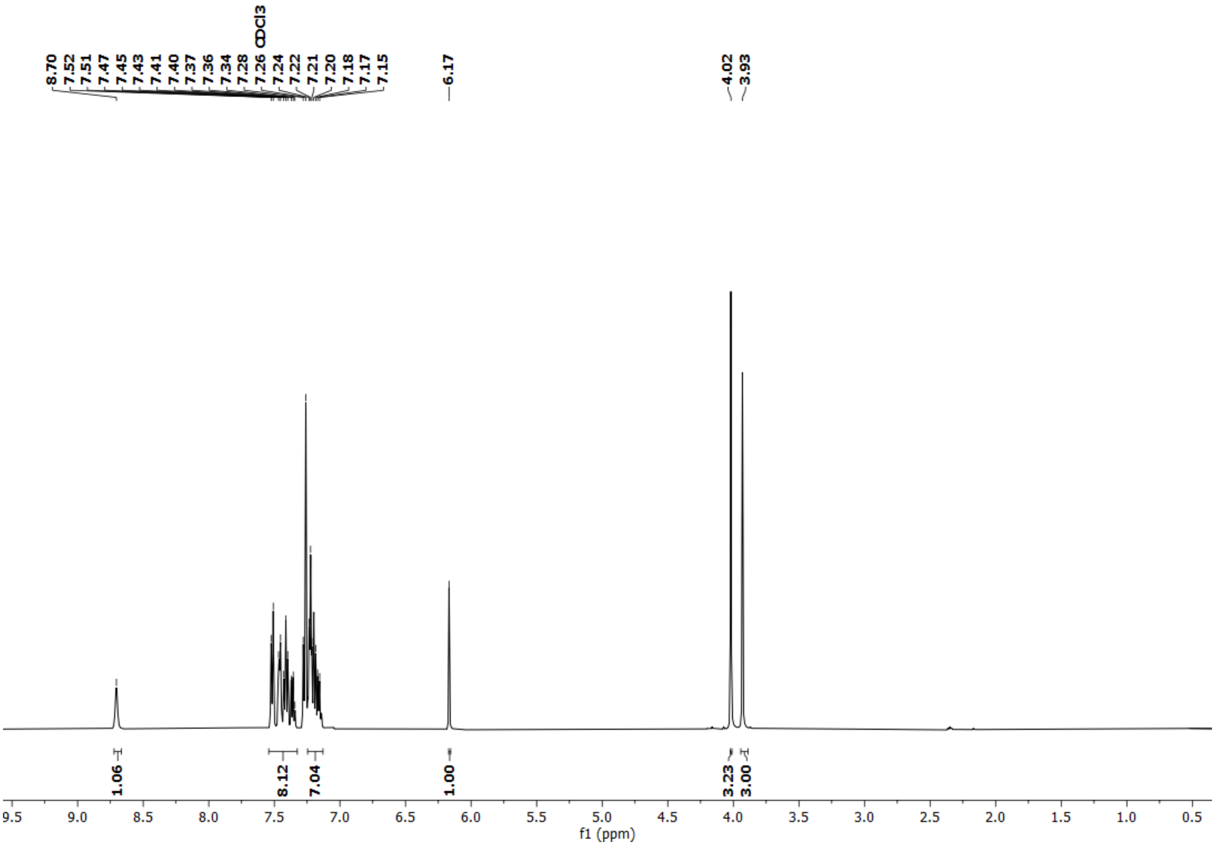

Figure S4. ^{1}H NMR Spectrum of compound 5a.

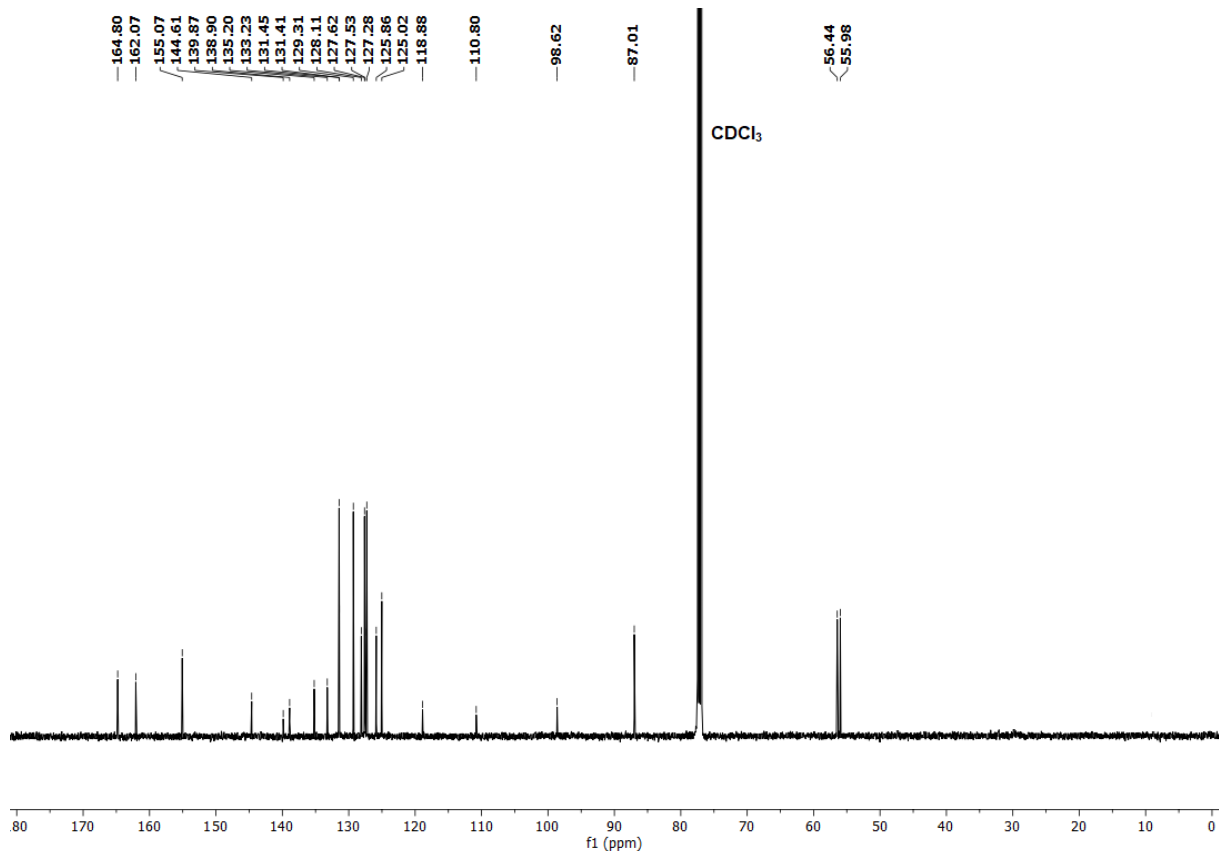

Figure S5. ^{13}C NMR Spectrum of compound 5a.

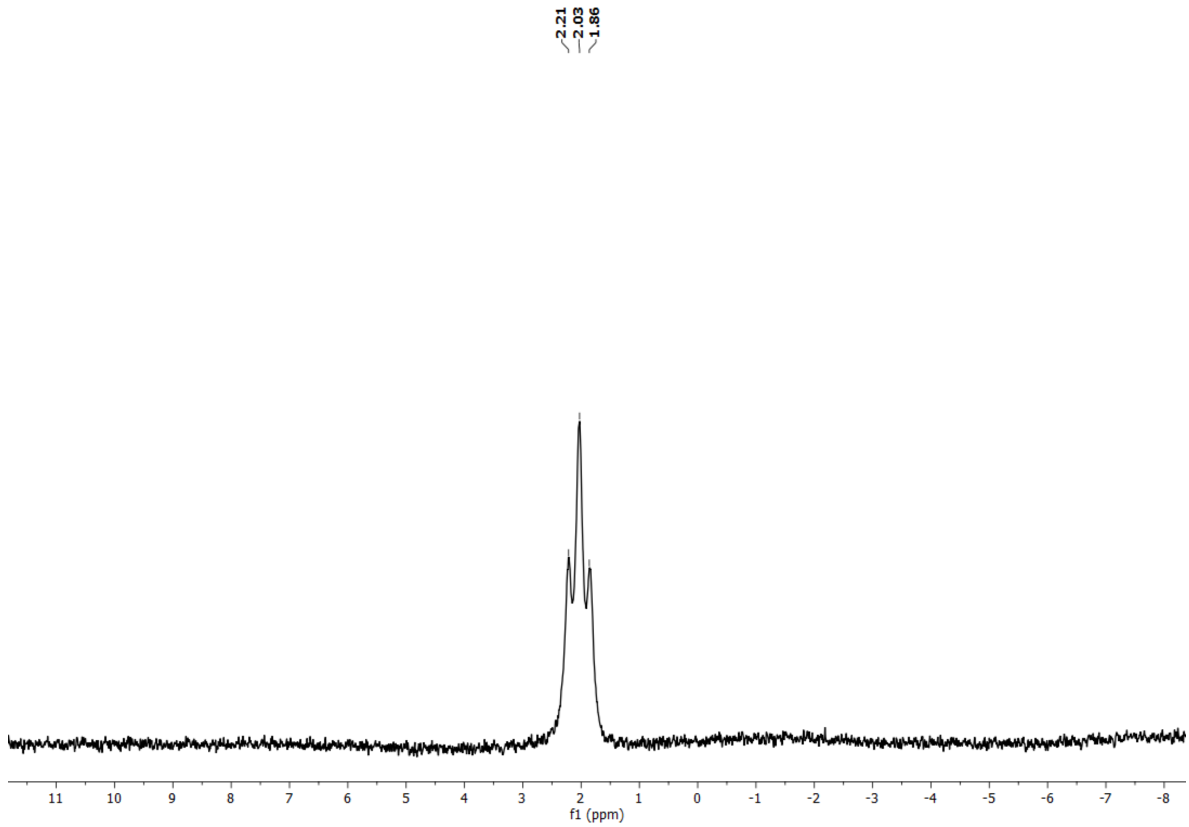

Figure S6. ^{11}B NMR Spectrum of compound 5a.

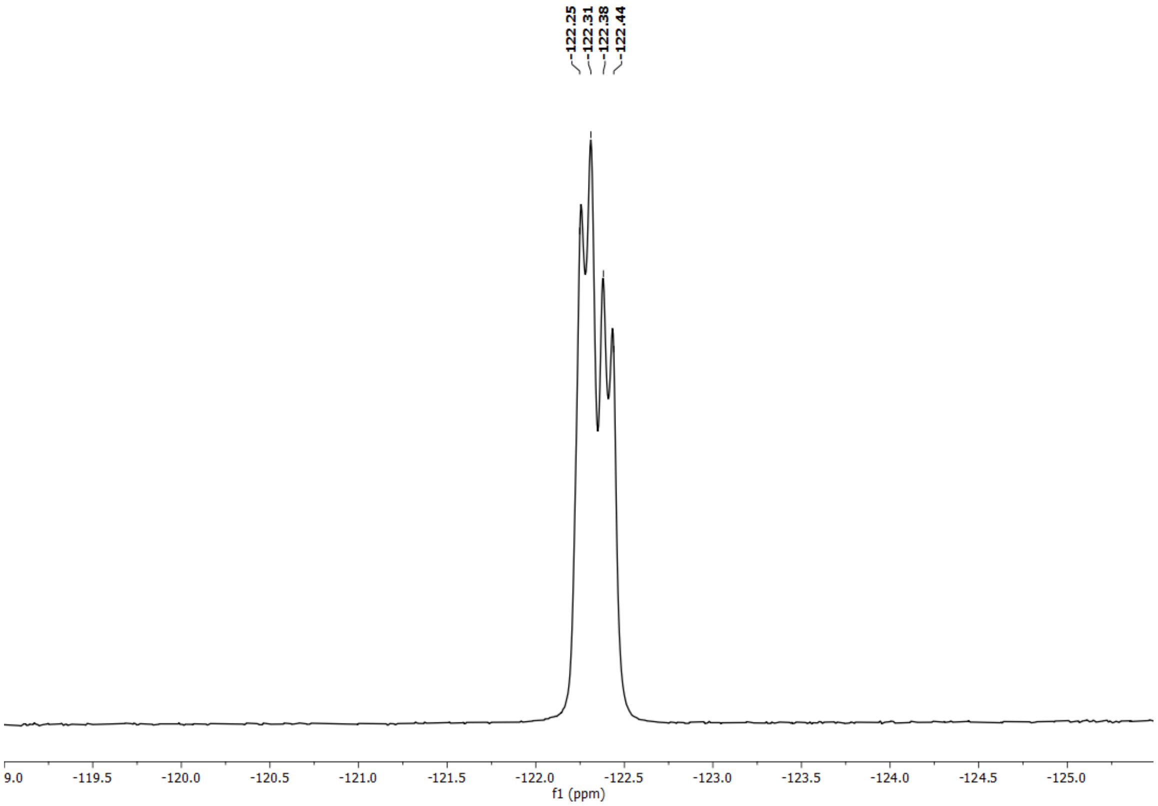

Figure S7. ^{19}F NMR Spectrum of compound 5a.

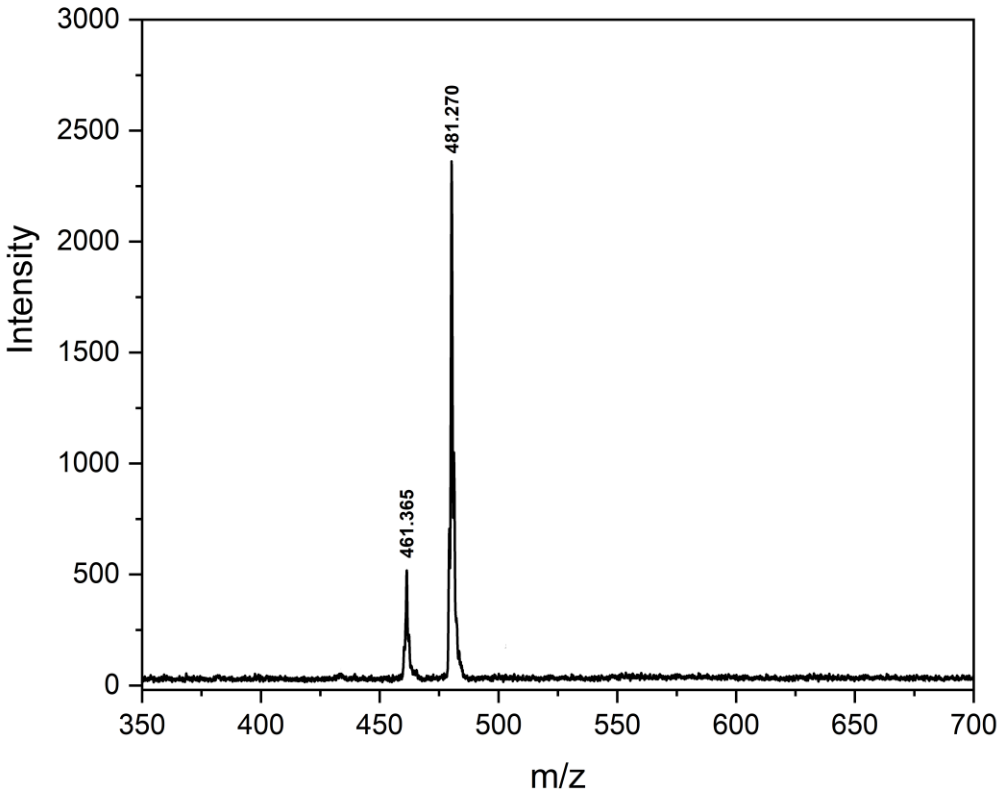

Figure S8. Positive ion and linear mode MALDI TOF-MS spectrum of compound 5a.

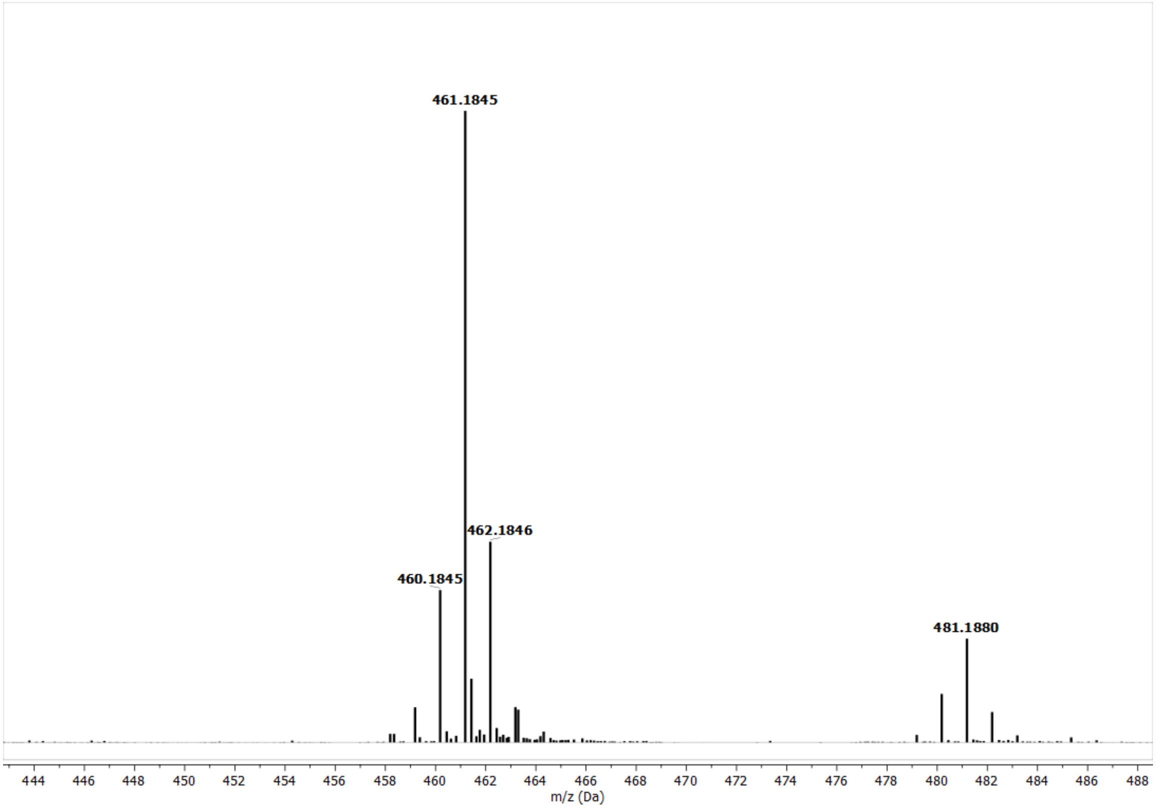

Figure S9. HRMS spectrum of compound 5a.

Table S1. Crystal data and refinement parameters for compound 5a.

**Empirical Formula**	$C_{29}H_{23}BF_2N_2O_2$
**Formula weight (g. mol^{-1})**	480.30
**Temperature (K)**	172.98
**Wavelength (Å)**	MoKα (λ = 0.71073)
**Crystal system**	Triclinic
**Space group**	P -1
**a/Å**	8.0415(8)
**b/Å**	11.1551(10)
**c/Å**	13.1228(12)
**α/°**	95.6130(10)
**β/°**	90.9290(10)
**γ/°**	96.0250(10)
**Crystal size/mm^3**	0.217 × 0.165 × 0.165
**V/ Å^3**	1164.63(19)
**Z**	2
**ρcalcd (g. cm^{-3})**	1.370
**μ (mm^{-1})**	0.096
**F(000)**	500.0
**2θ range for data collection/°**	3.12 to 49.998
**h/k/l**	$-9 \leq h \leq 9$, $-12 \leq k \leq 13$, $-15 \leq l \leq 15$
**Reflections collected**	12505
**Independent reflections**	4094 [R_{int} = 0.0244
**Data/restraints/parameters**	4094/0/327
**Goodness-of-fit on F^2 (S)**	1.056
**Final R indices [I > 2σ(I)]**	R_1 = 0.0362, wR_2 = 0.0923
**R indices (all data)**	R_1 = 0.0442, wR_2 = 0.0958
**Largest diff. peak/hole / e Å^{-3}**	0.18/-0.23

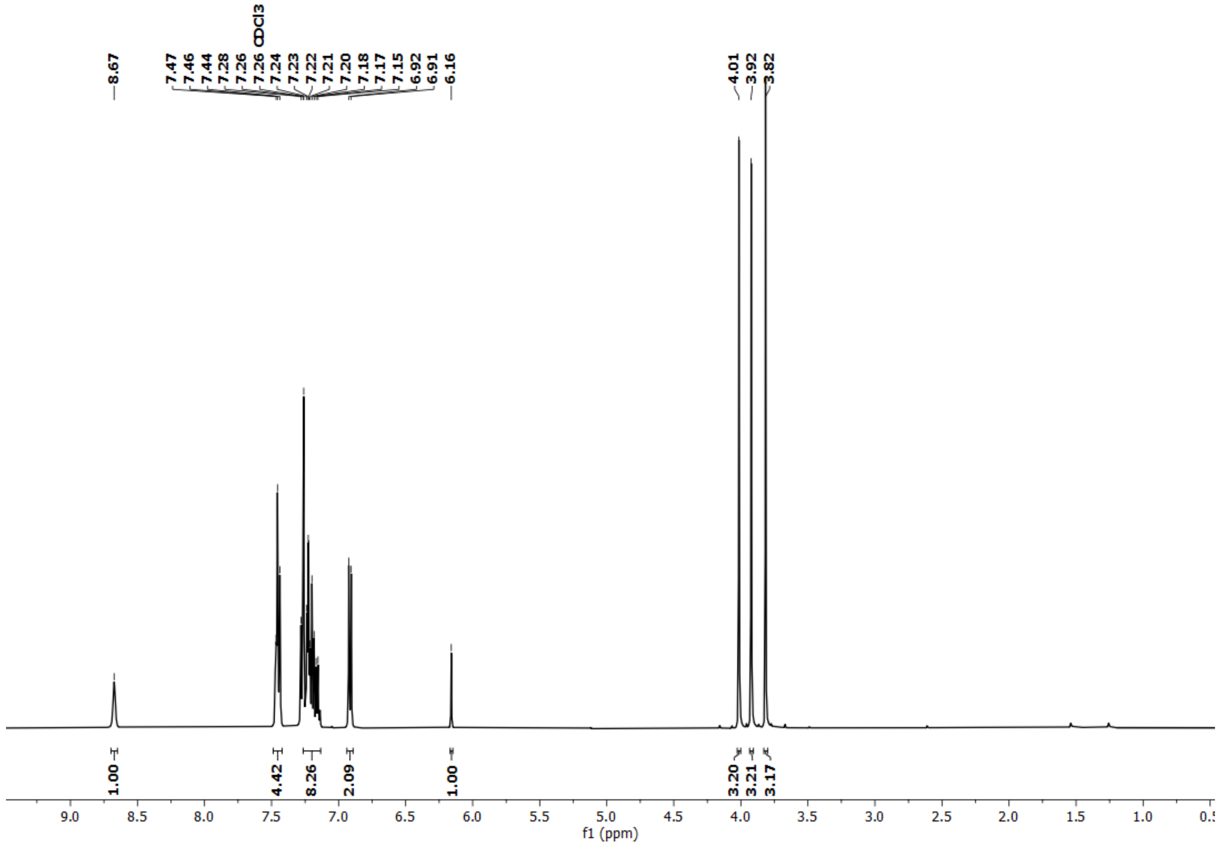

Figure S10. ^{1}H NMR Spectrum of compound 5b.

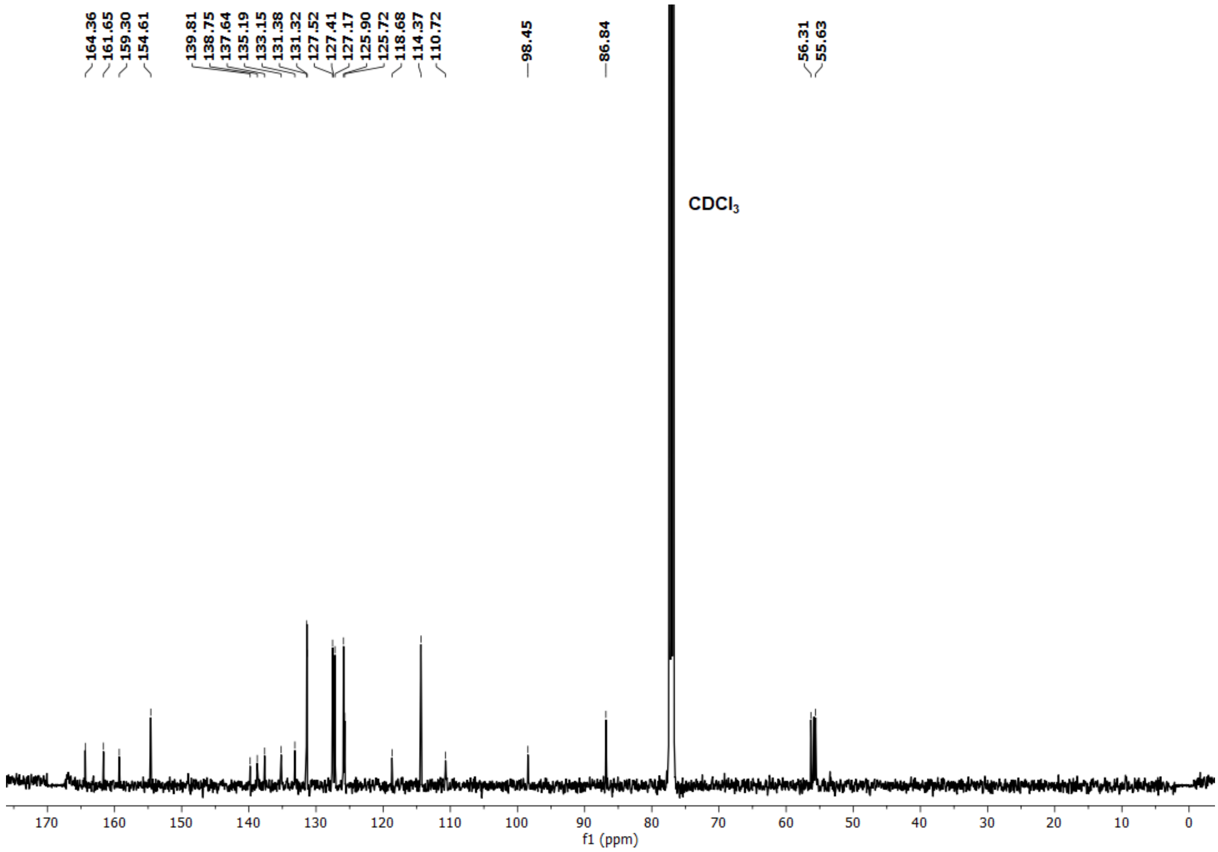

Figure S11. ^{13}C NMR Spectrum of compound 5b.

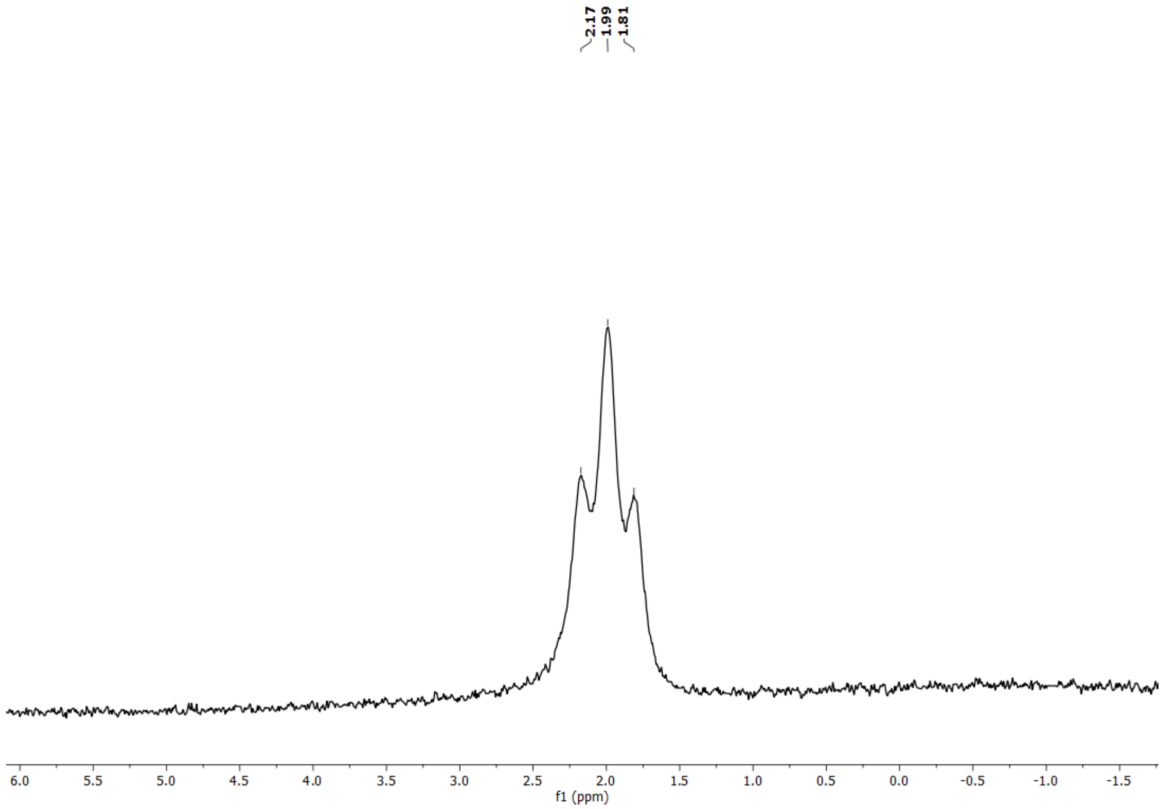

Figure S12. ^{11}B NMR Spectrum of compound 5b

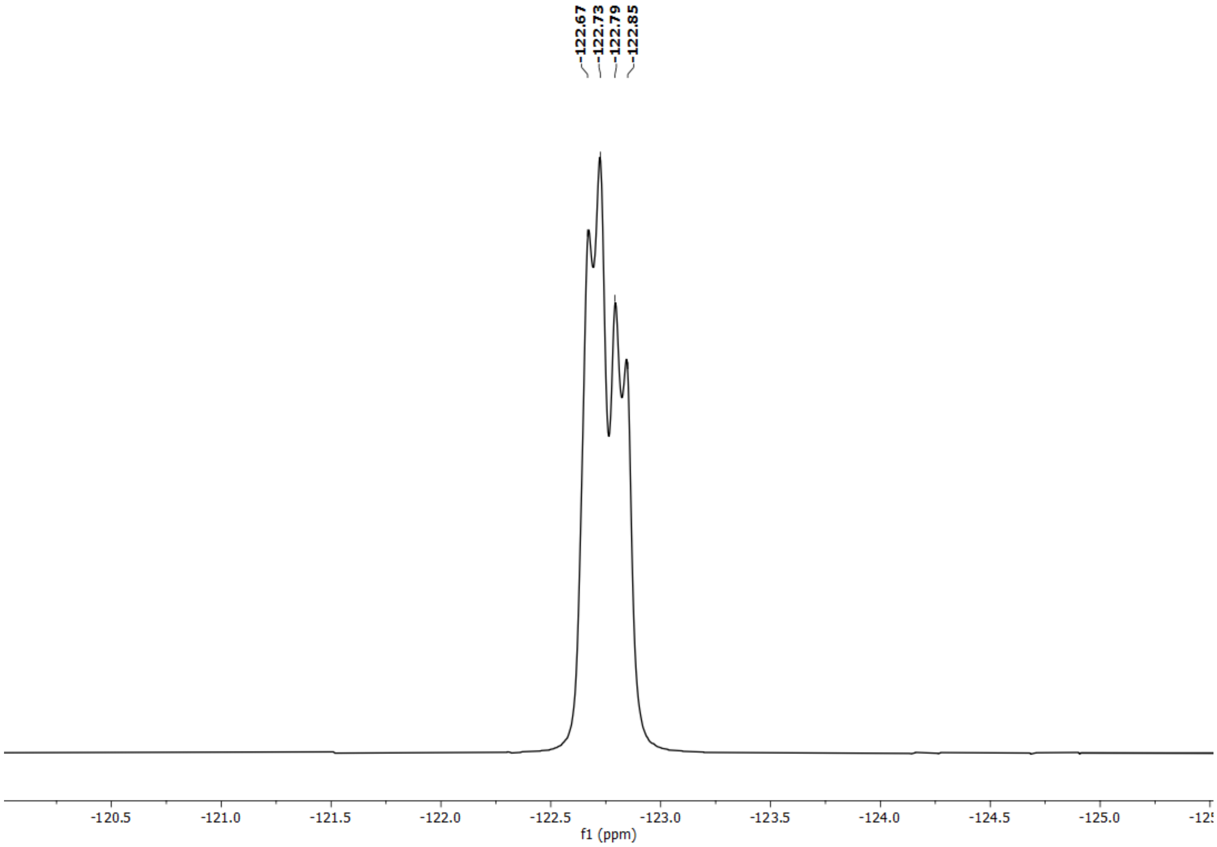

Figure S13. ^{19}F NMR Spectrum of compound 5b

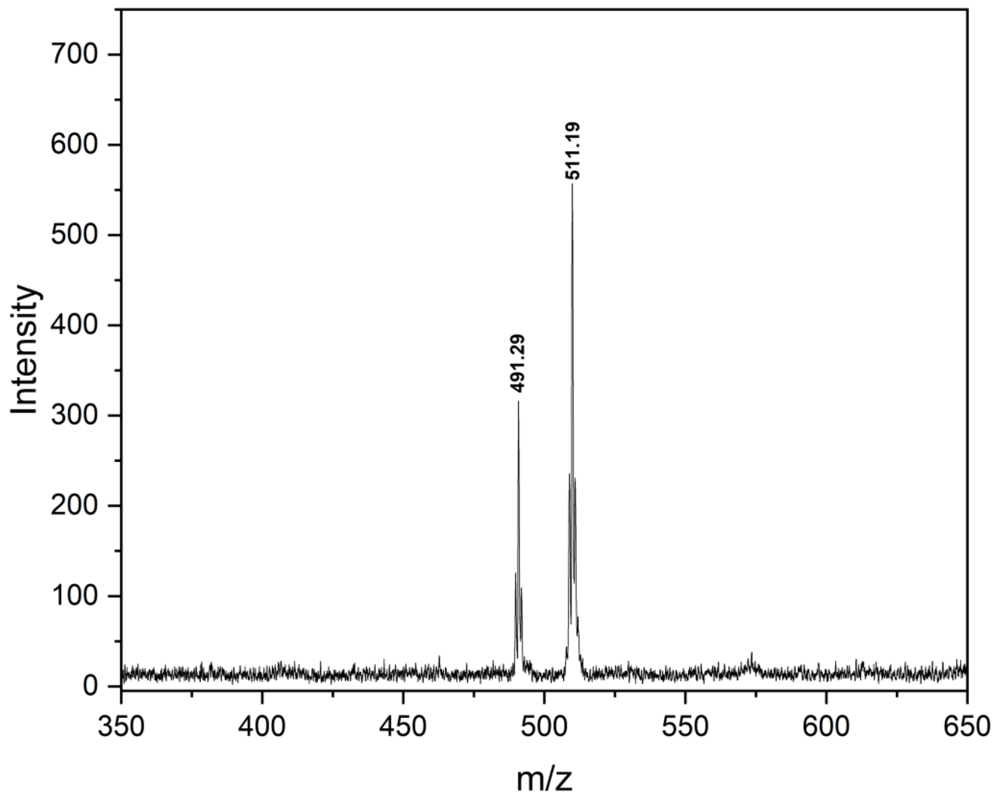

Figure S14. Positive ion and linear mode MALDI TOF-MS spectrum of compound 5b.

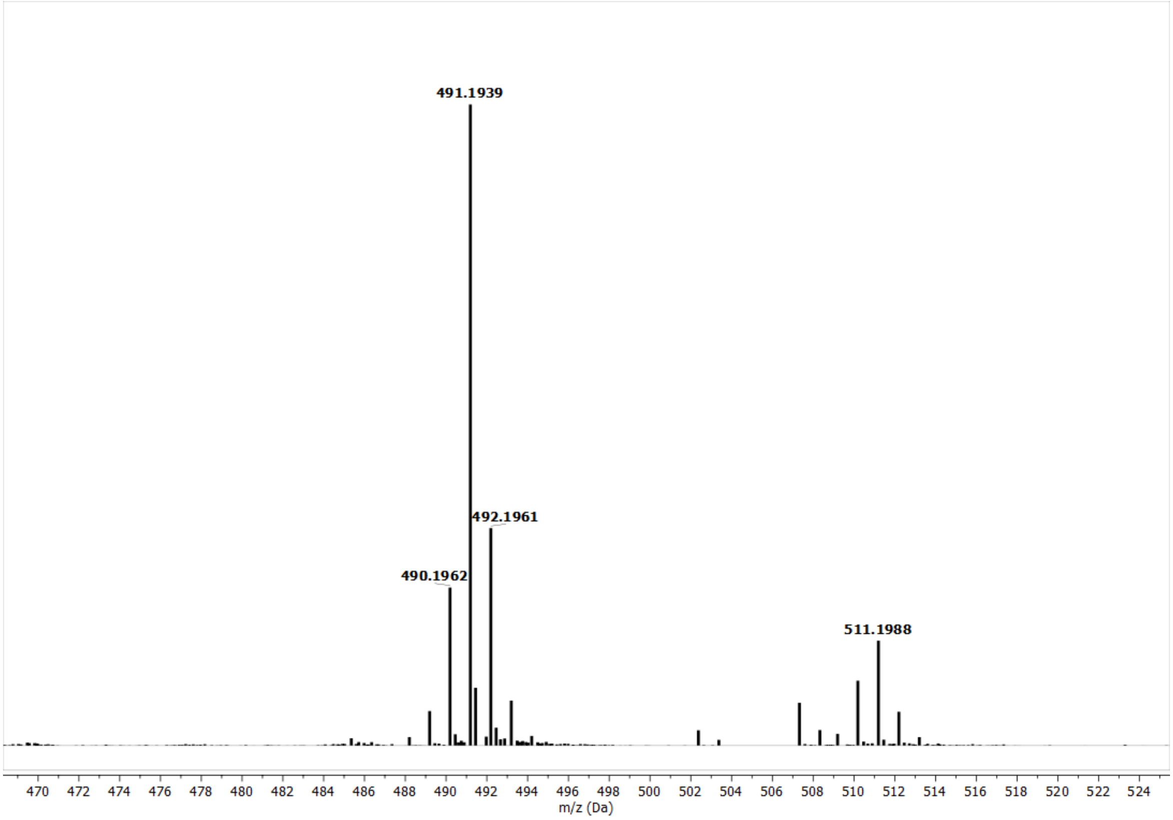

Figure S15. HRMS spectrum of compound 5b.

Table S2. Crystal data and refinement parameters for compound 5b.

**Empirical Formula**	$C_{30}H_{25}BF_2N_2O_3$
**Formula weight/g. mol^{-1}**	510.33
**Temperature/K**	173.01
**Wavelength (Å)**	MoKα (λ = 0.71073)
**Crystal System**	Monoclinic
**Space group**	Pc
**a/Å**	12.841(5)
**b/Å**	27.674(10)
**c/Å**	7.714(3)
**α/°**	90
**β/°**	105.021(5)
**γ/°**	90
**Crystal size/mm^3**	0.321 × 0.217 × 0.108
**V/ Å^3**	2647.7(16)
**Z**	2
**ρcalcd (g. cm^{-3})**	1.280
**μ (mm^{-1})**	0.092
**F(000)**	1064.0
**θ range for data collection/°**	1.472 to 50.05
**h/k/l**	$-15 \le h \le 15$, $-32 \le k \le 32$, $-9 \le l \le 9$
**Reflections collected**	28521
**Independent reflections**	9145 [R_{int} = 0.0564
**Data/restraints/parameters**	9145/2/691
**Goodness-of-fit on F^2 (S)**	1.326
**Final R indices [I > 2σ(I)]**	R_1 = 0.0973, wR_2 = 0.2794
**R indices (all data)**	R_1 = 0.1019, wR_2 = 0.2871
**Largest diff. peak/hole / e Å^{-3}**	0.85/-0.51

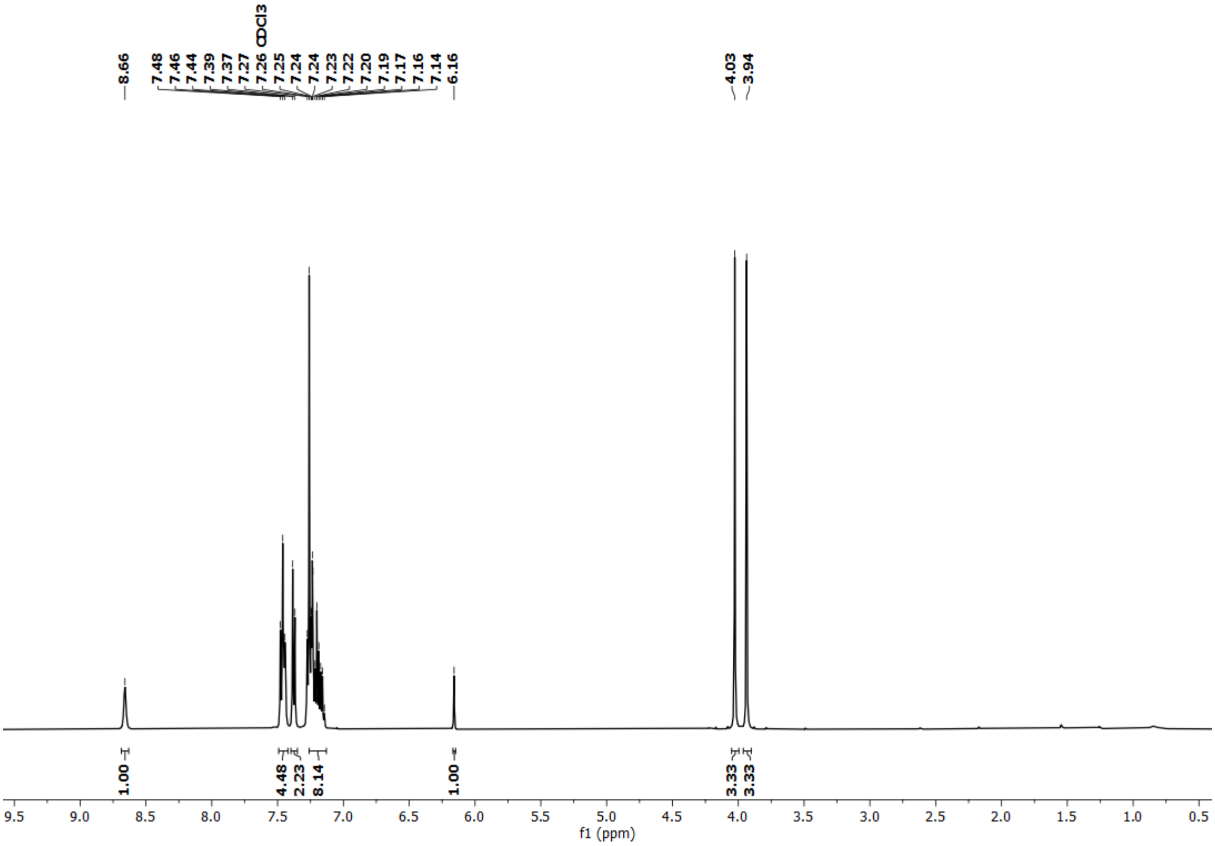

Figure S16. ^{1}H NMR Spectrum of compound 5c.

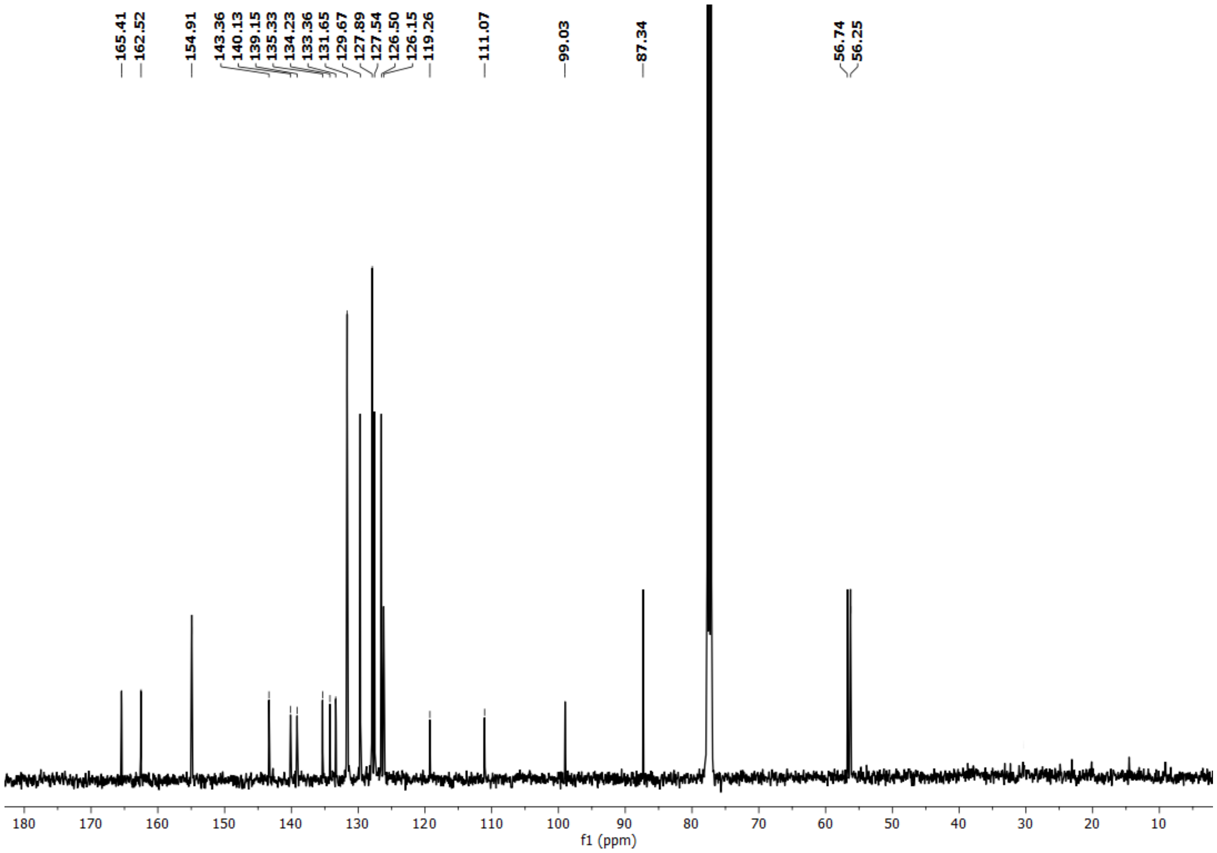

Figure S17. ^{13}C NMR Spectrum of compound 5c.

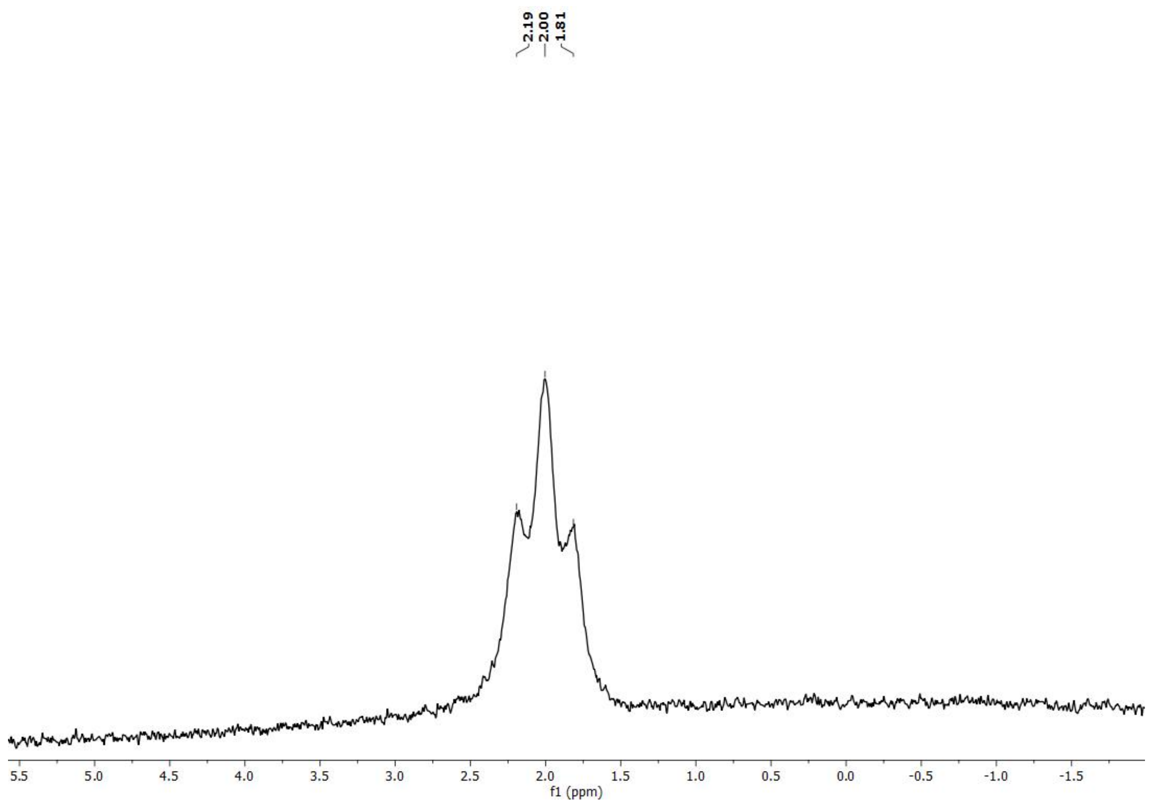

Figure S18. ^{11}B NMR Spectrum of compound 5c

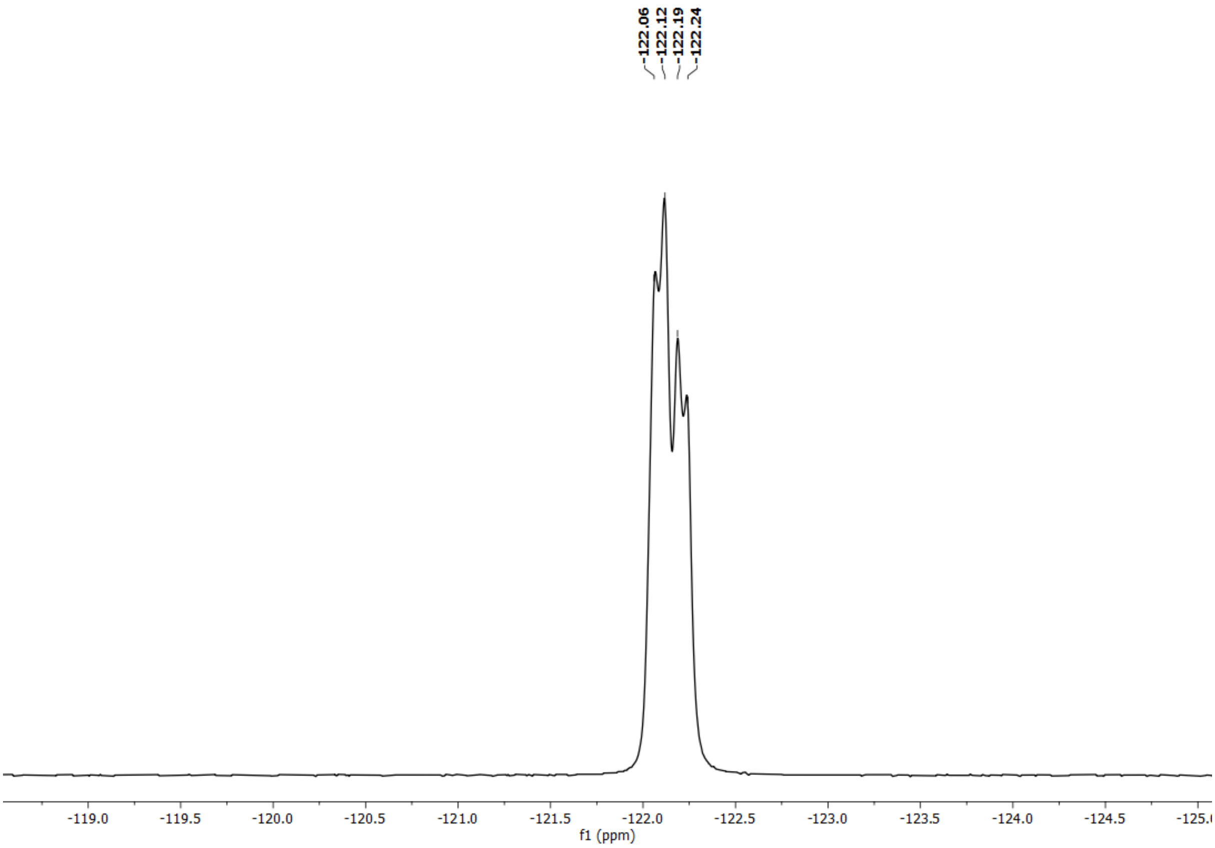

Figure S19. ^{19}F NMR Spectrum of compound 5c

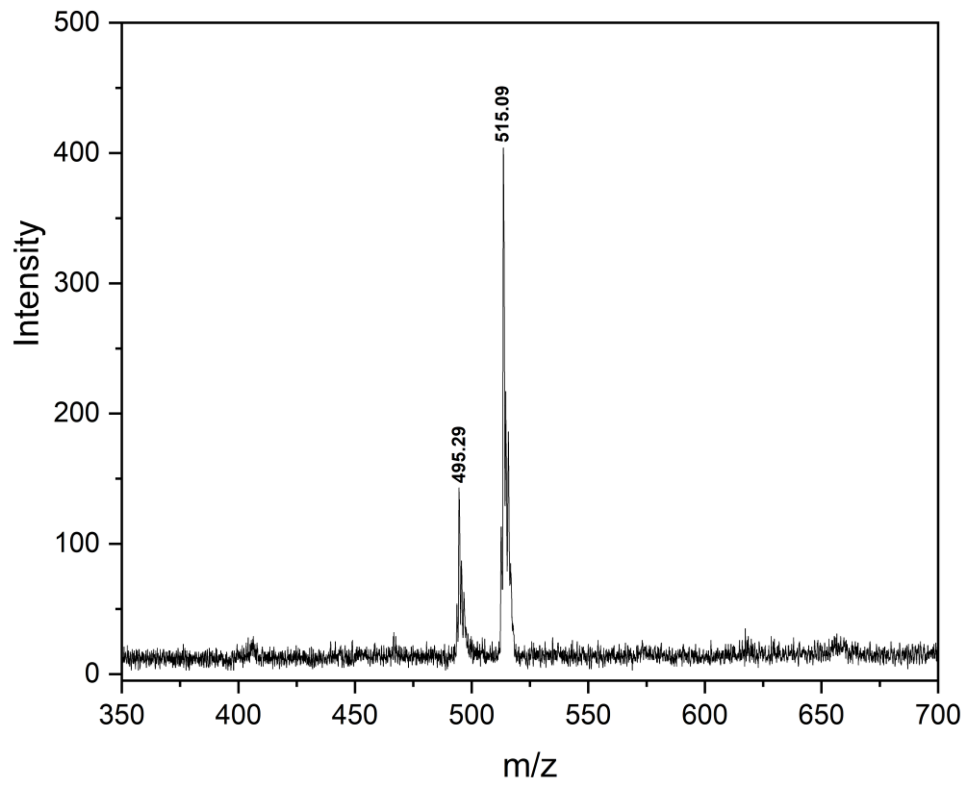

Figure S20. Positive ion and linear mode MALDI TOF-MS spectrum of compound 5c.

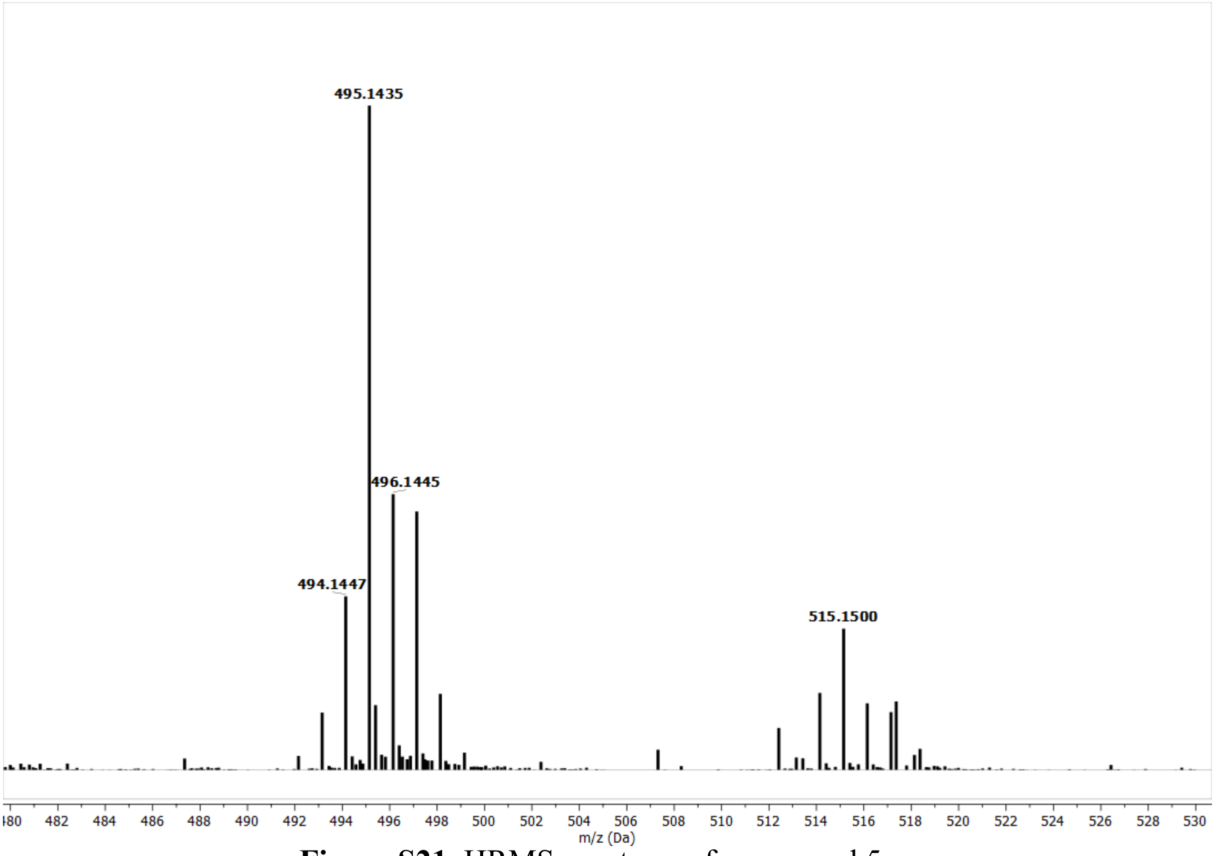

Figure S21. HRMS spectrum of compound 5c.

Table S3. Crystal data and refinement parameters for compound 5c.

**Empirical Formula**	$C_{29}H_{22}BClF_2N_2O_2$
**Formula weight/g. mol^{-1}**	514.74
**Temperature/K**	298.97
**Wavelength (Å)**	MoKα (λ = 0.71073)
**Crystal system**	Monoclinic
**Space group**	$P2_1/n$
**a/Å**	13.6918(18)
**b/Å**	13.1667(17)
**c/Å**	14.1141(19)
**α/°**	90
**β/°**	92.015(6)
**γ/°**	90
**Crystal size/mm^3**	0.234 × 0.224 × 0.124
**V/ Å^3**	2542.9(6)
**Z**	4
**ρcalcd (g. cm^{-3})**	1.345
**μ (mm^{-1})**	0.195
**F(000)**	1064.0
**2θ range for data collection/°**	4.074 to 49.998
**h/k/l**	$-16 \leq h \leq 16$, $-15 \leq k \leq 15$, $-16 \leq l \leq 15$
**Reflections collected**	18580
**Independent reflections**	4470 [R_{int} = 0.0720
**Data/restraints/parameters**	4470/6/336
**Goodness-of-fit on F^2 (S)**	1.069
**Final R indices [I > 2σ(I)]**	R_1 = 0.0553, wR_2 = 0.1342
**R indices (all data)**	R_1 = 0.1049, wR_2 = 0.1530
**Largest diff. peak/hole / e Å^{-3}**	0.36/-0.27

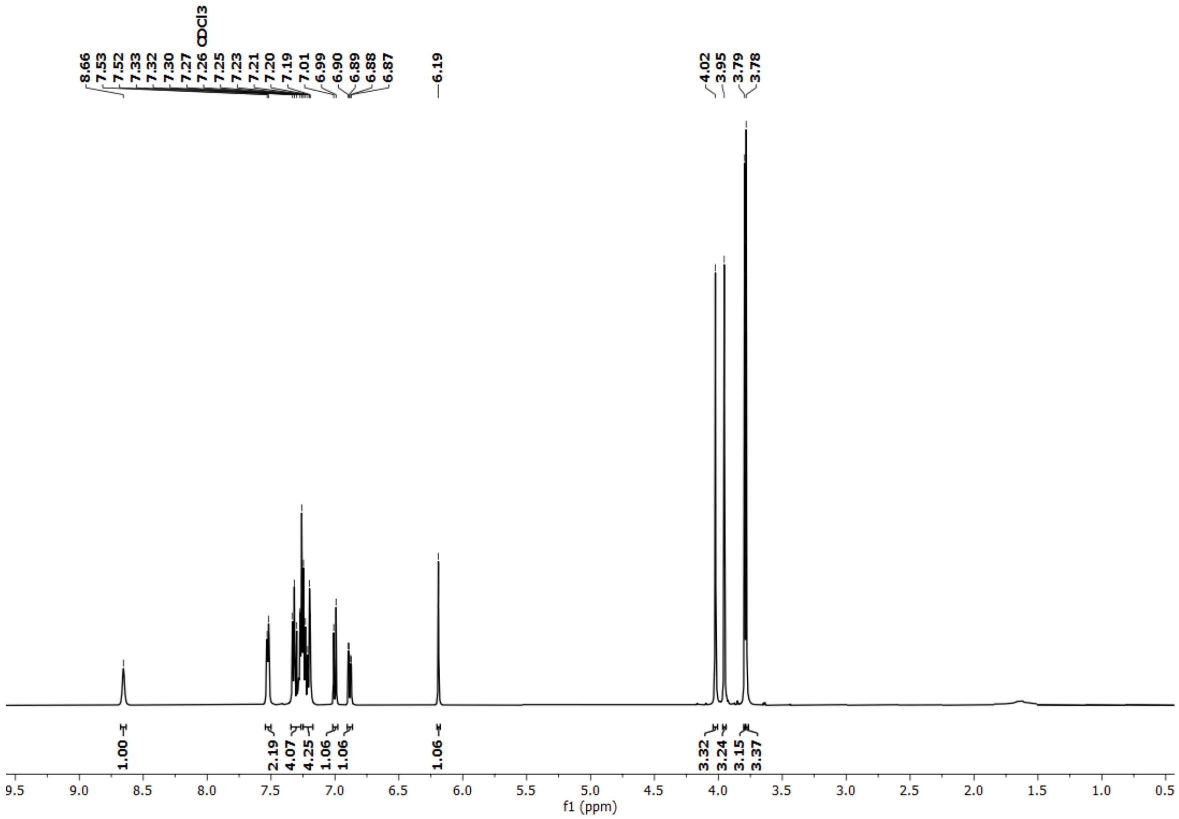

Figure S22. ^{1}H NMR Spectrum of compound 5d.

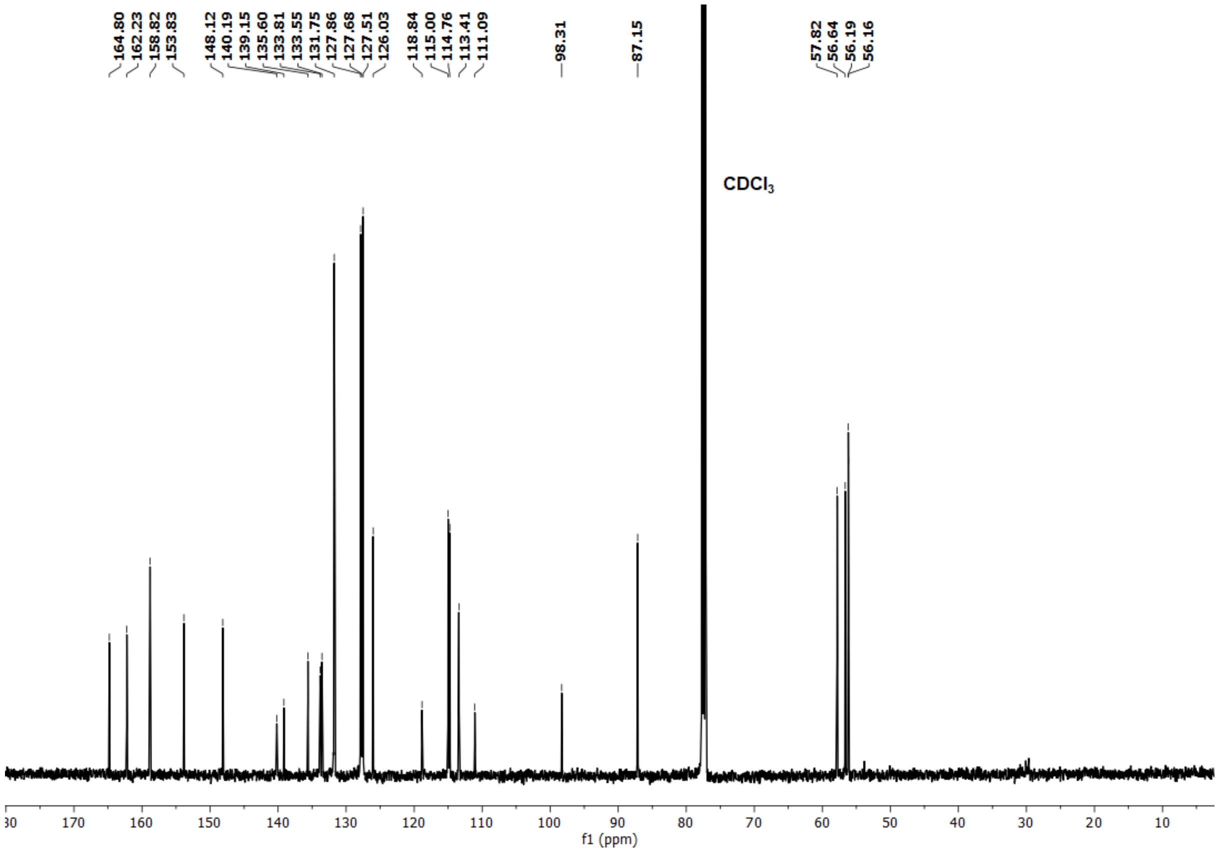

Figure S23. ^{13}C NMR Spectrum of compound 5d.

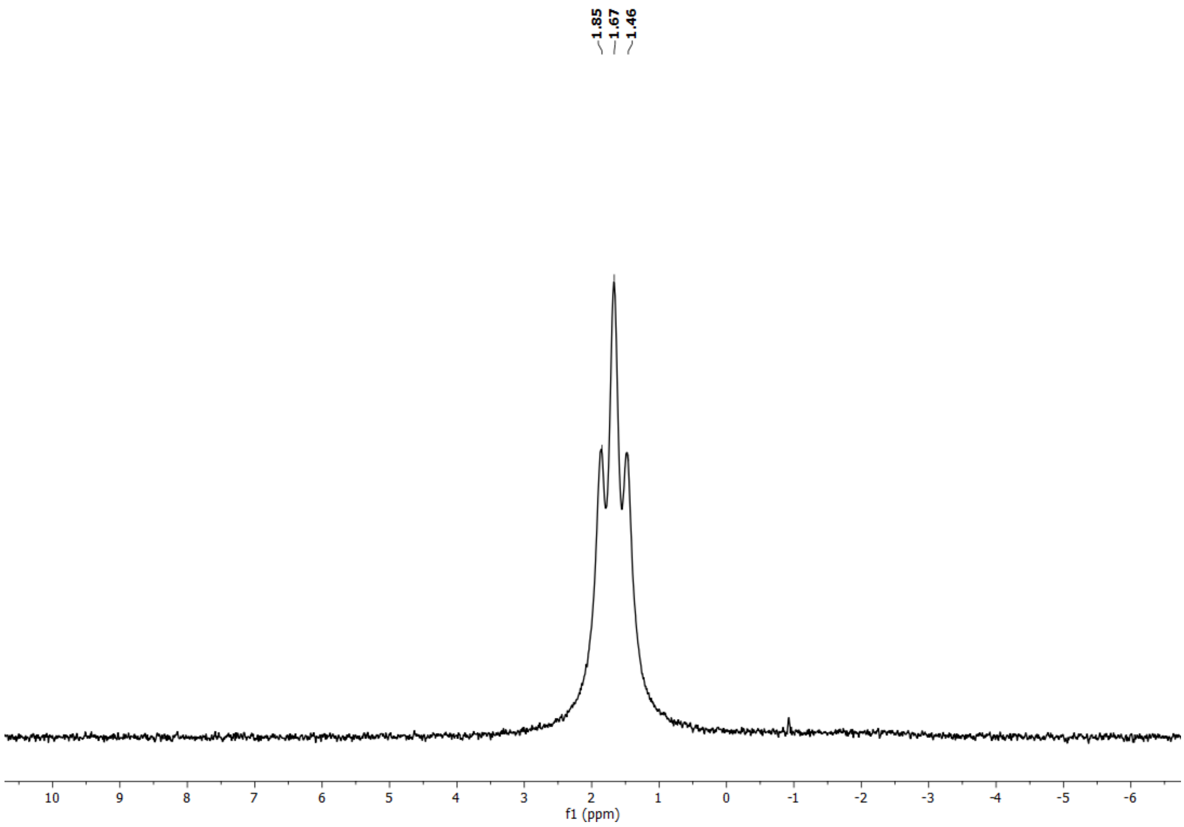

Figure S24. ^{11}B NMR Spectrum of compound 5d.

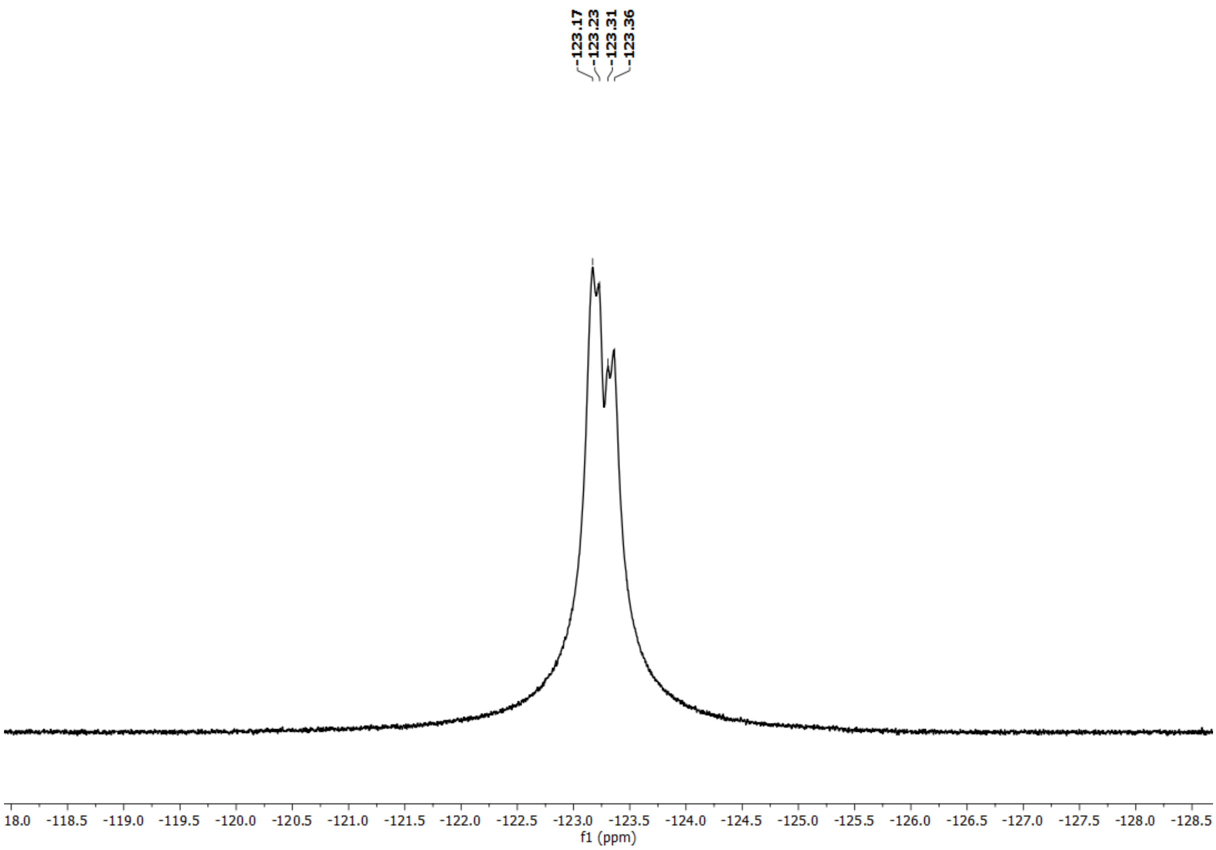

Figure S25. ^{19}F NMR Spectrum of compound 5c

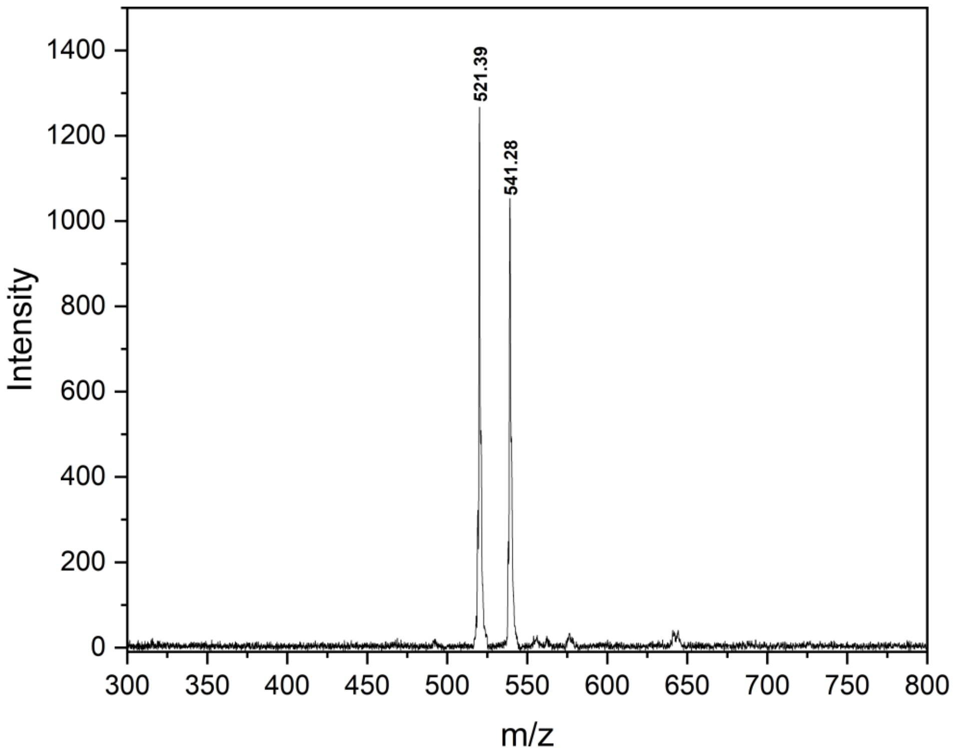

Figure S26. Positive ion and linear mode MALDI TOF-MS spectrum of compound 5d.

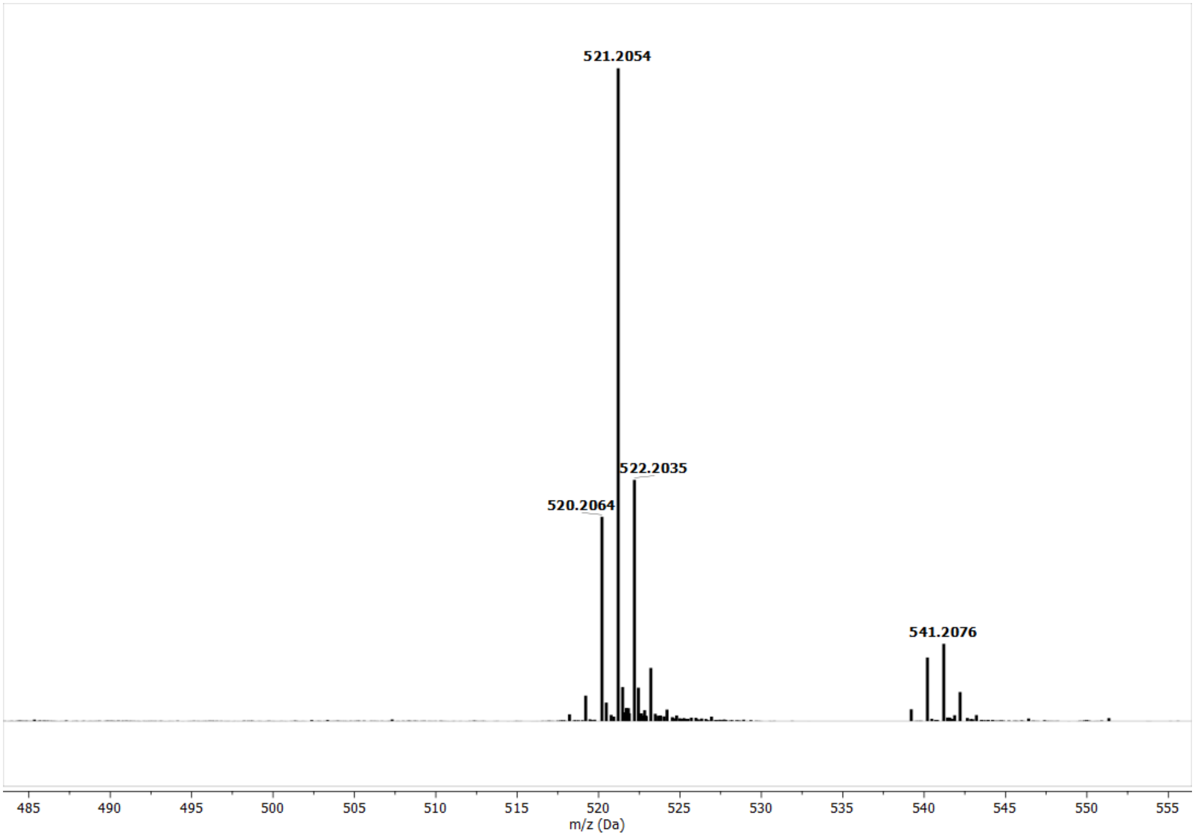

Figure S27. HRMS spectrum of compound 5d.

Table S4. Crystal data and refinement parameters for compound 5d.

**Empirical Formula**	$C_{31}H_{27}BF_2N_2O_4$
**Formula weight/g. mol^{-1}**	540.35
**Temperature/K**	273.15
**Wavelength (Å)**	MoKα (λ = 0.71073)
**Crystal system**	Monoclinic
**Space group**	$P2_1/n$
**a/Å**	15.429(9)
**b/Å**	28.160(16)
**c/Å**	25.611(14)
**α/°**	90
**β/°**	91.647(9)
**γ/°**	90
**Crystal size/mm^3**	0.224 × 0.217 × 0.117
**V/ Å^3**	11122(11)
**Z**	16
**ρcalcd (g. cm^{-3})**	1.291
**μ (mm^{-1})**	0.094
**F(000)**	4512.0
**2θ range for data collection/°**	2.892 to 50
**h/k/l**	-18 ≤ h ≤ 18, -33 ≤ k ≤ 33, -30 ≤ l ≤ 30
**Reflections collected**	172891
**Independent reflections**	19577 [Rint = 0.1111
**Data/restraints/parameters**	19577/0/1458
**Goodness-of-fit on F^2 (S)**	1.048
**Final R indices [I > 2σ(I)]**	R1 = 0.0658, wR2 = 0.1616
**R indices (all data)**	R1 = 0.1501, wR2 = 0.1891
**Largest diff. peak/hole / e Å^{-3}**	0.34/-0.30

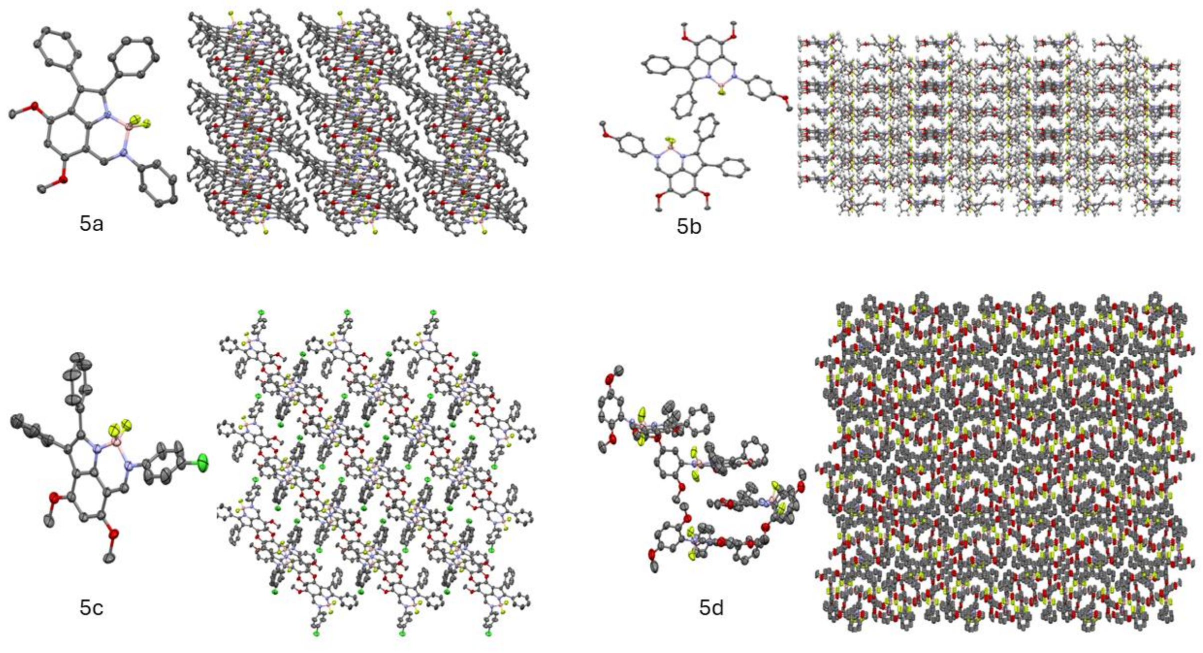

Figure S28. Asymmetric unit cell (left) for the single-crystal of 5a-d and molecular packing (right) along the crystallographic network.

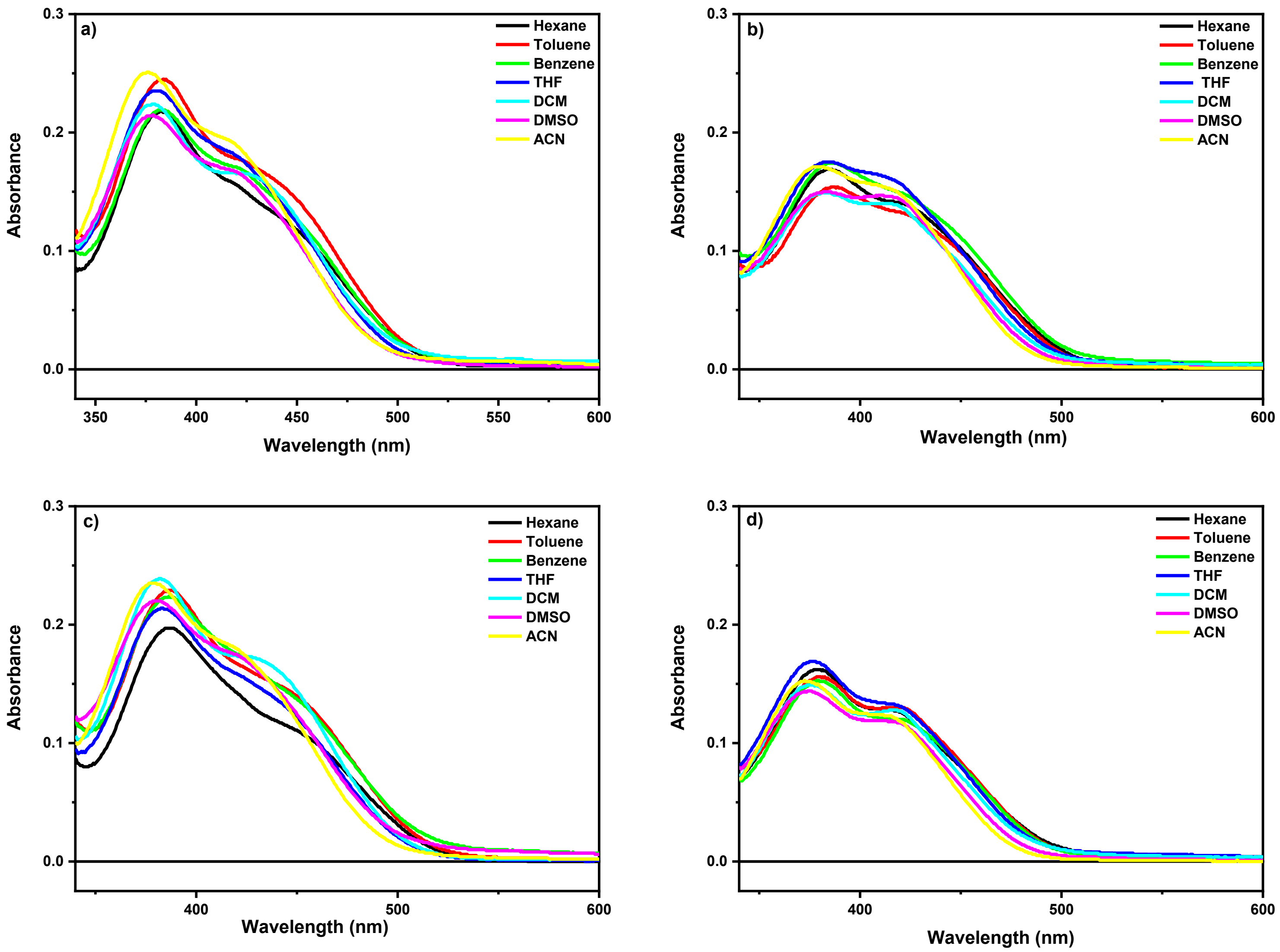

Figure S29. Steady-state absorption spectra of compounds a) 5a, b) 5b, c) 5c and d) 5d in solvent with several polarities.

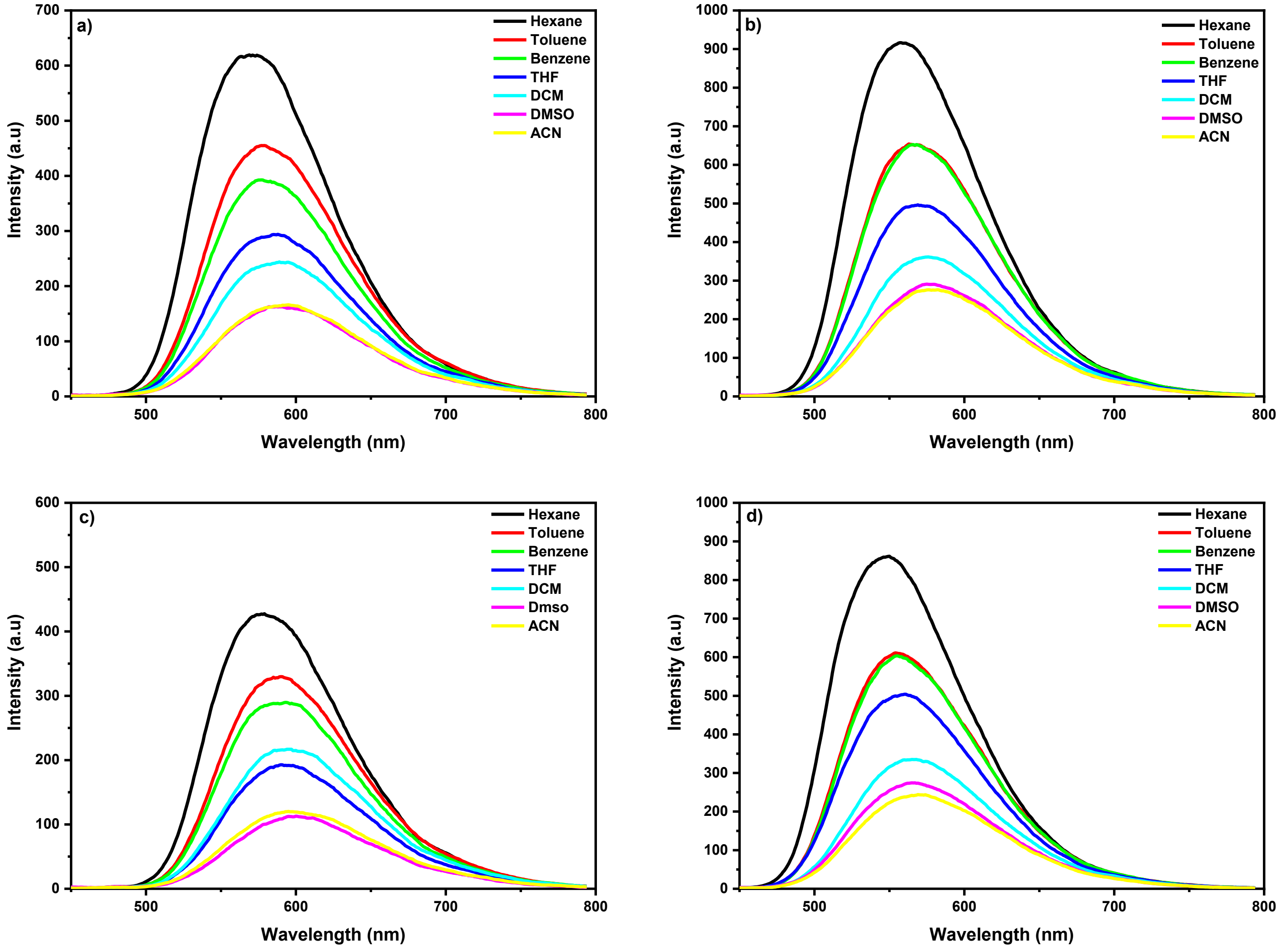

Figure S30. Steady-state fluorescence emission spectra of compounds a) 5a, b) 5b, c) 5c and d) 5d in solvent with several polarities. Excitation at 420 nm.

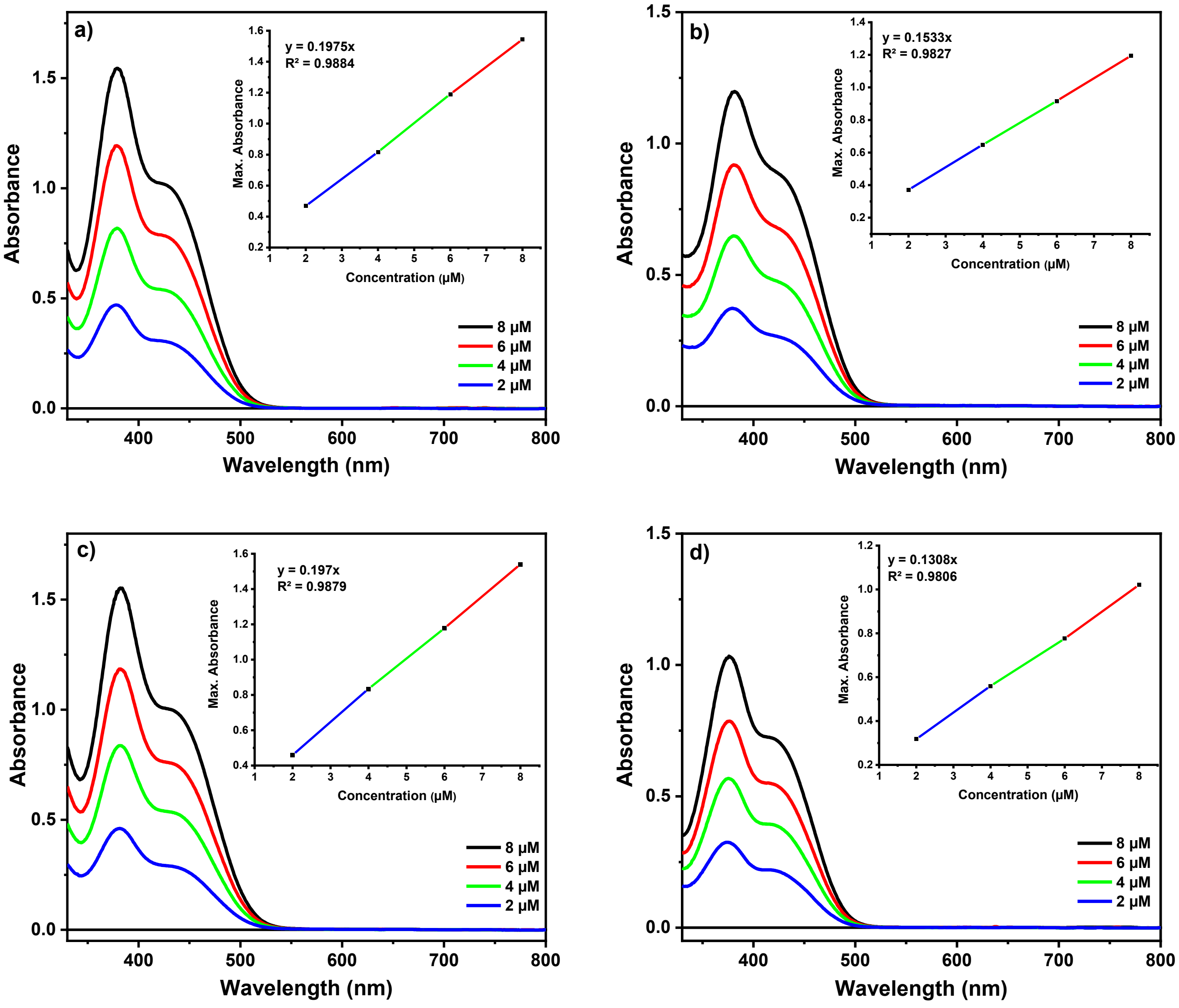

Figure S31. Absorption spectra of compounds a) 5a, b) 5b, c) 5c and d) 5d in chloroform at different concentrations: $8x10^{-6}$ M, $6x10^{-6}$ M, $4x10^{-6}$ M, $2x10^{-6}$ M.

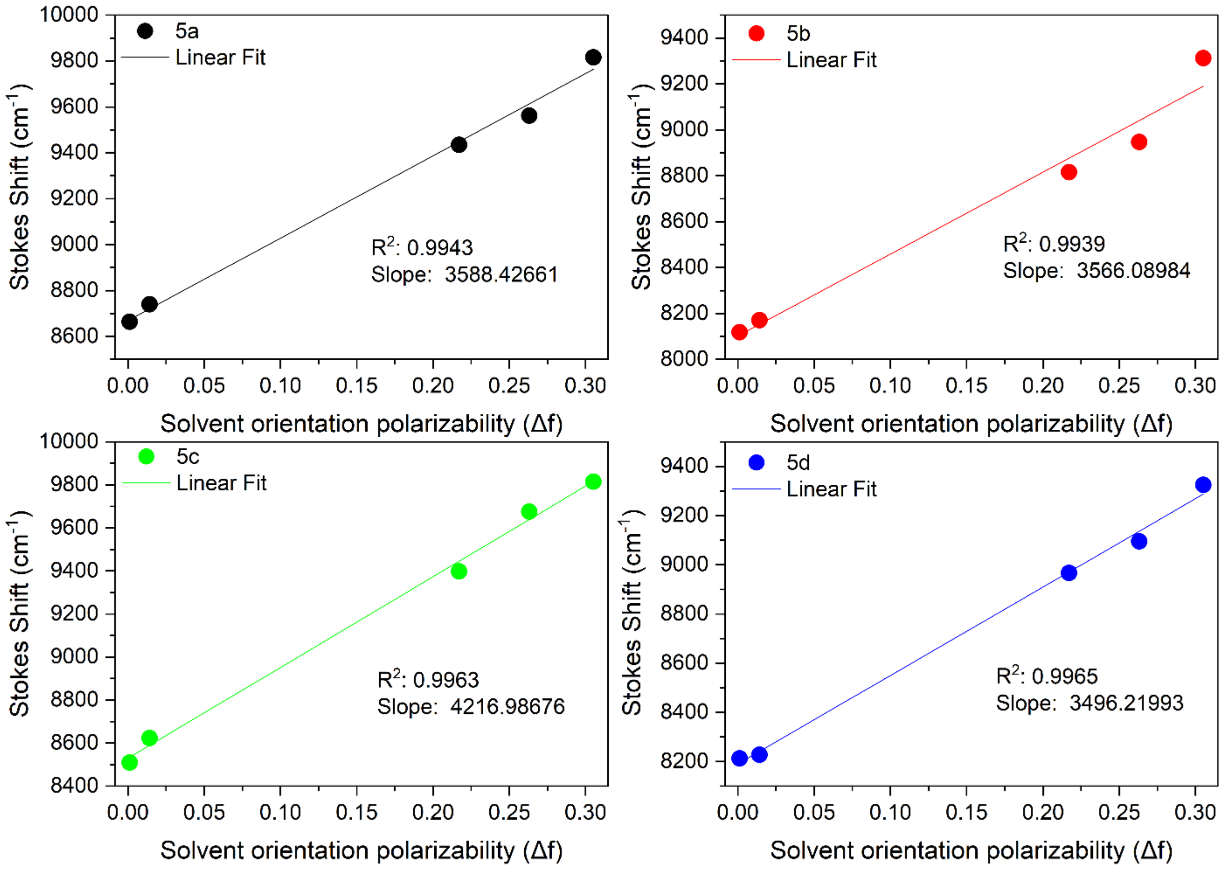

Figure S32. Plot of Stokes shift versus solvent orientation polarizability for compounds 5a–d.

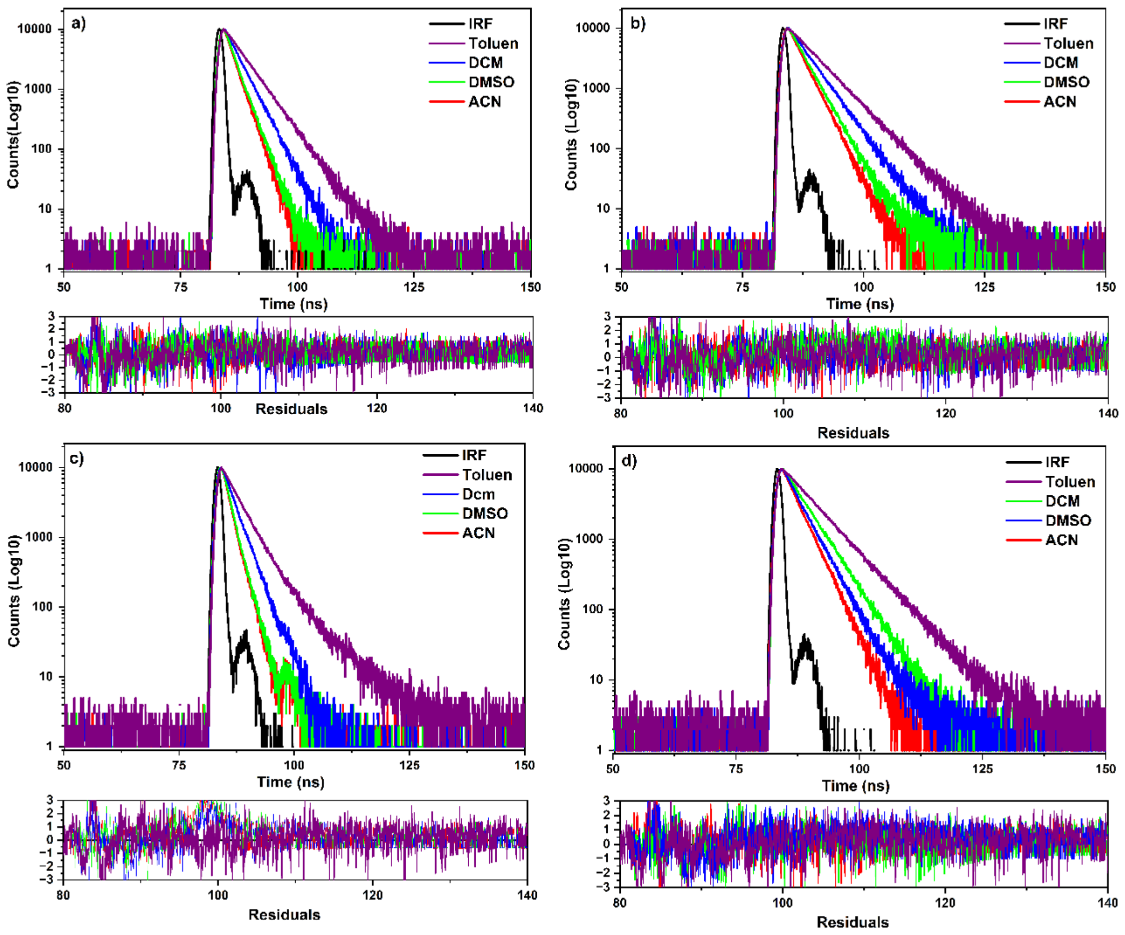

Figure S33. The fluorescence decay profiles of compounds a) 5a, b) 5b, c) 5c and d) 5d in solvent with several polarities by excitation 390 nm.

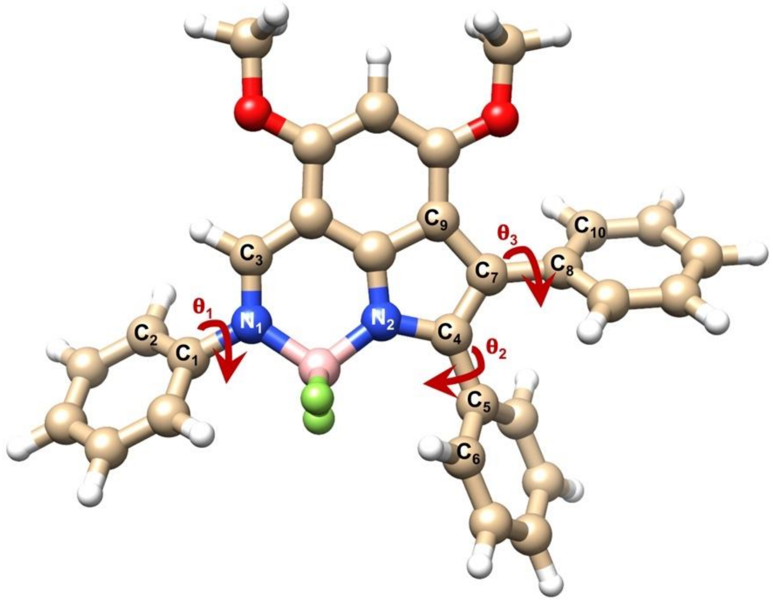

Figure S34. Geometrical parameters for the investigated indolyl-imine-based N-N boron complexes.

Table S5. Calculated geometrical parameters for 5a-d.

Compounds	Geometry	θ_1 (degrees)	θ_2 (degrees)	θ_3 (degrees)	C_1-N_1 (Å)	C_4-C_5 (Å)	C_7-C_8 (Å)
5a	S_0	46.2	-54.0	-55.8	1.44	1.48	1.48
	S_1	32.2	-46.1	-45.5	1.41	1.46	1.46
5b	S_0	46.6	-53.6	-55.7	1.44	1.48	1.48
	S_1	34.4	-46.6	-45.3	1.42	1.46	1.46
5c	S_0	45.4	-54.2	-55.7	1.43	1.48	1.48
	S_1	30.7	-45.9	-45.6	1.41	1.46	1.46
5d	S_0	64.6	-53.3	-54.6	1.44	1.48	1.48
	S_1	52.1	-46.3	-45.20	1.42	1.46	1.46

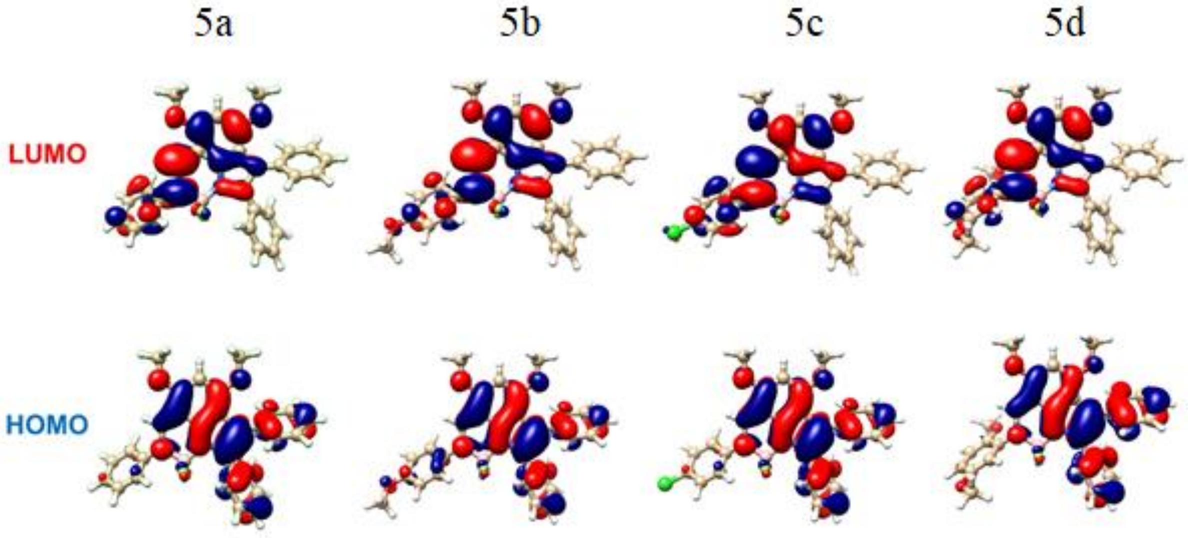

Figure S35. HOMO and LUMO energies for 5a-d in S_0 geometries.

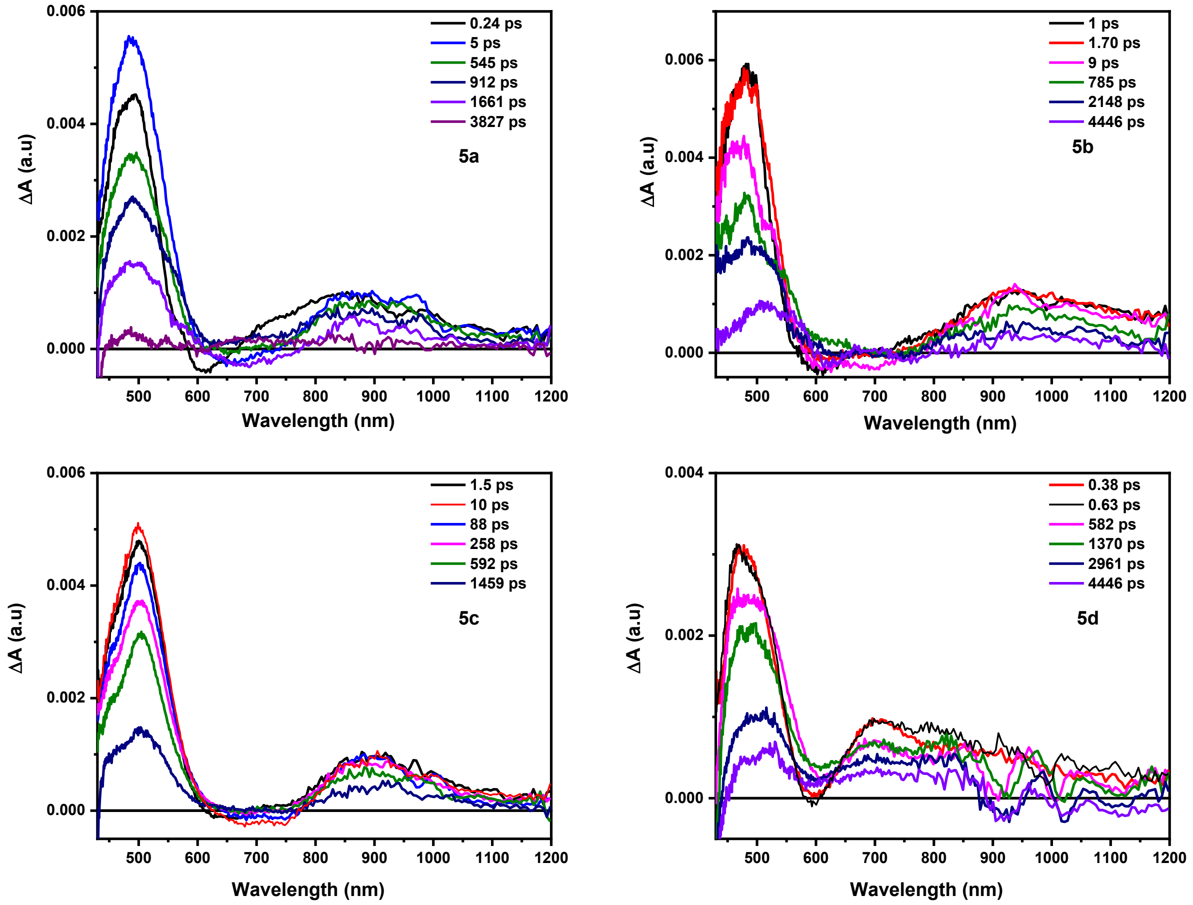

Figure S36. Transient absorption spectra of 5a-d in acetonitrile (Acn) after excitation at 420 nm. The transient absorption spectra were measured at the time delays indicated in the panels.

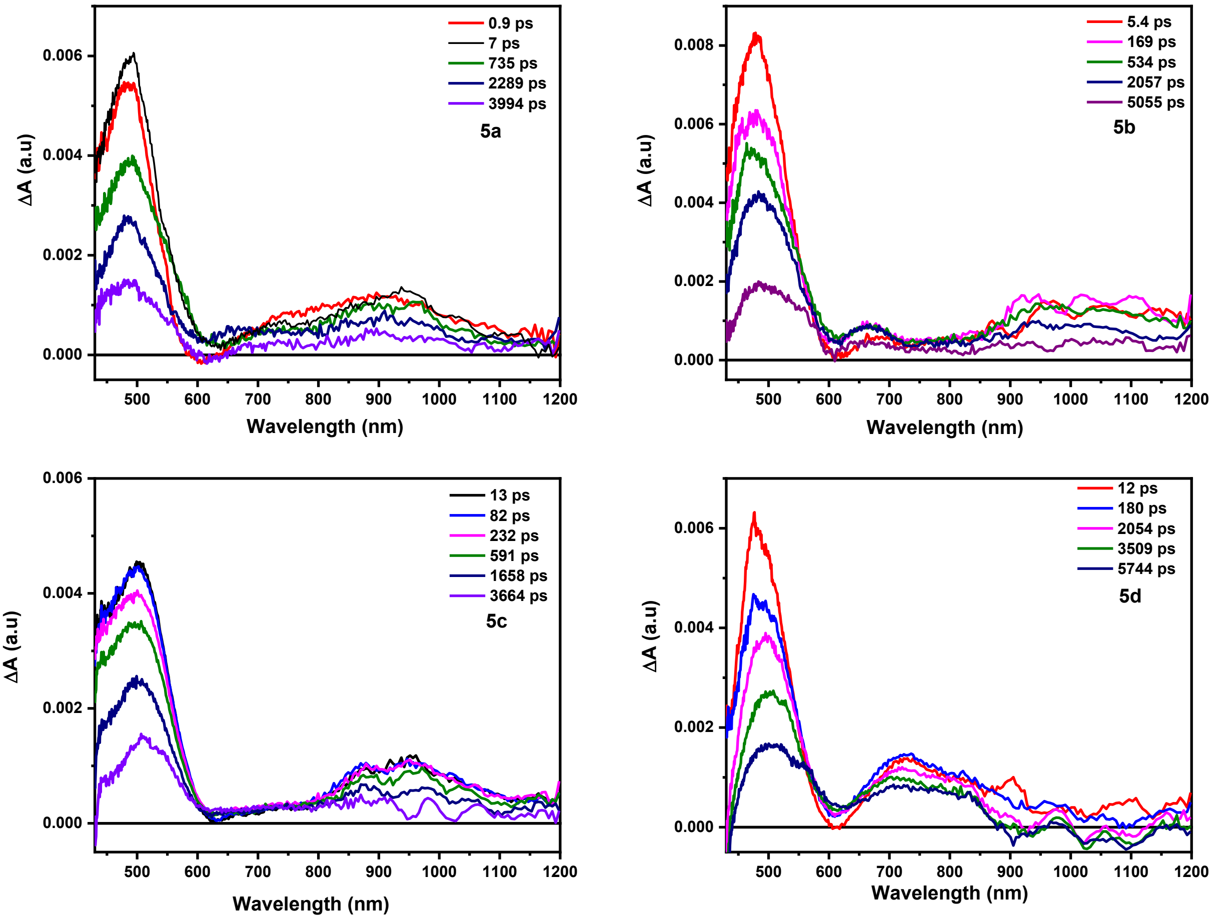

Figure S37. Transient absorption spectra of 5a-d in toluene (Tol) after excitation at 420 nm. The transient absorption spectra were measured at the time delays indicated in the panels.

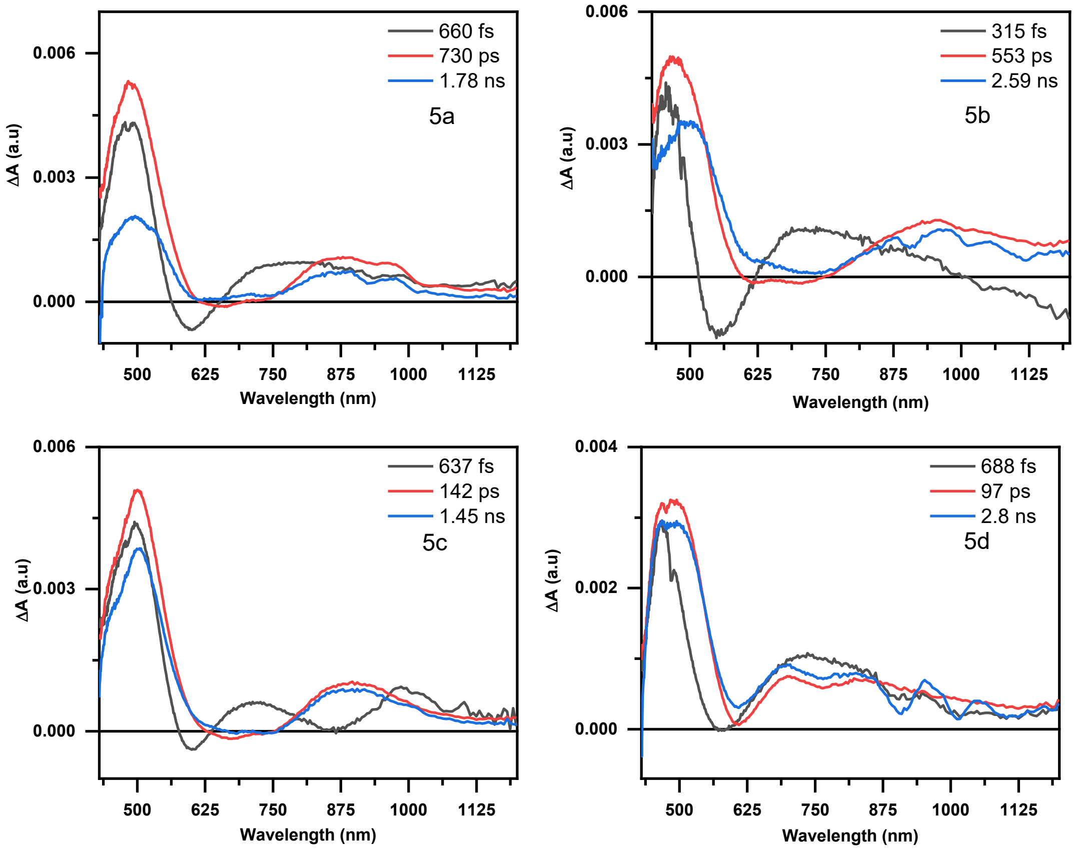

Figure S38. EADS data obtained from global fitting of 5a-d in acetonitrile.

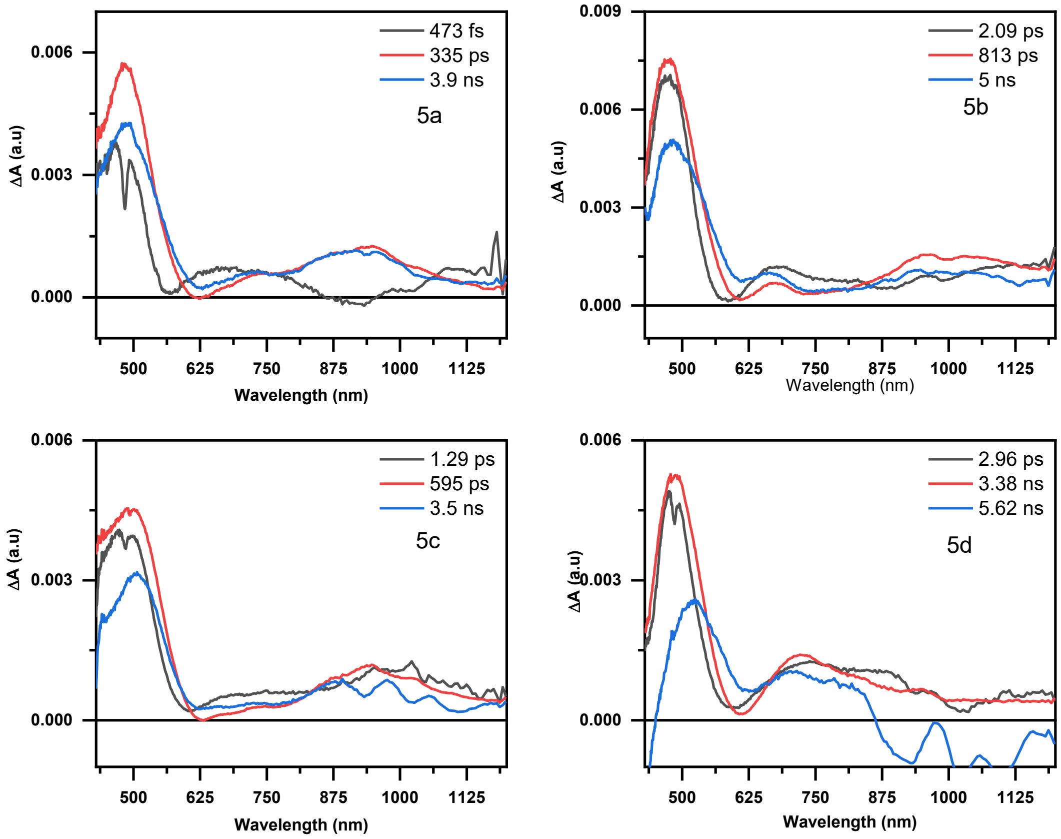

Figure S39. EADS data obtained from global fitting of 5a-d in toluene.

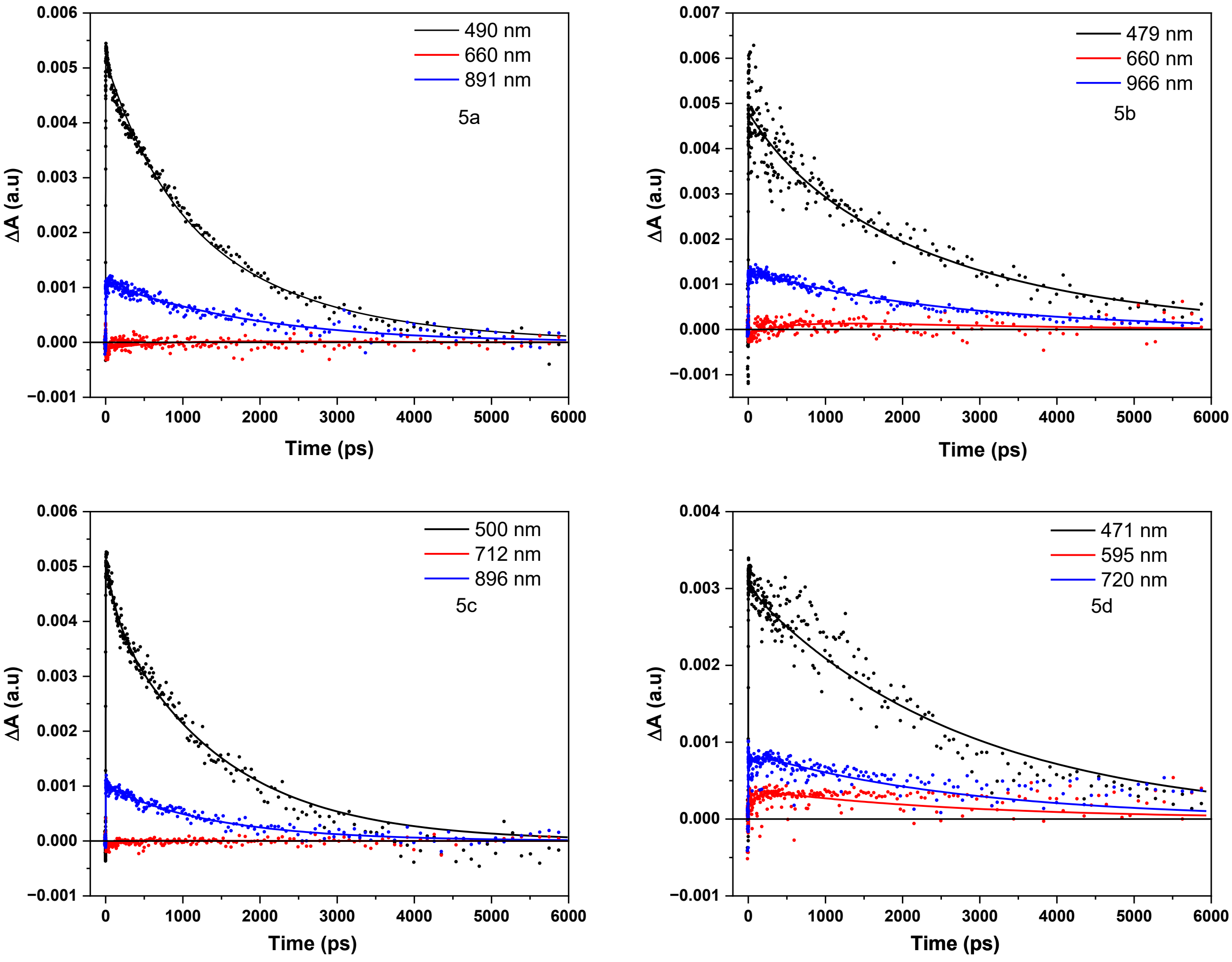

Figure S40. Comparison of the kinetics at the excited state absorption (S_1 maximum, black), stimulated emission (red), and NIR spectral region (blue) of compounds 5a-d in acetonitrile. The probing wavelengths for each molecule are shown in each panel Excitation was at 420 nm. The solids lines represent fits obtained from global fitting analysis.

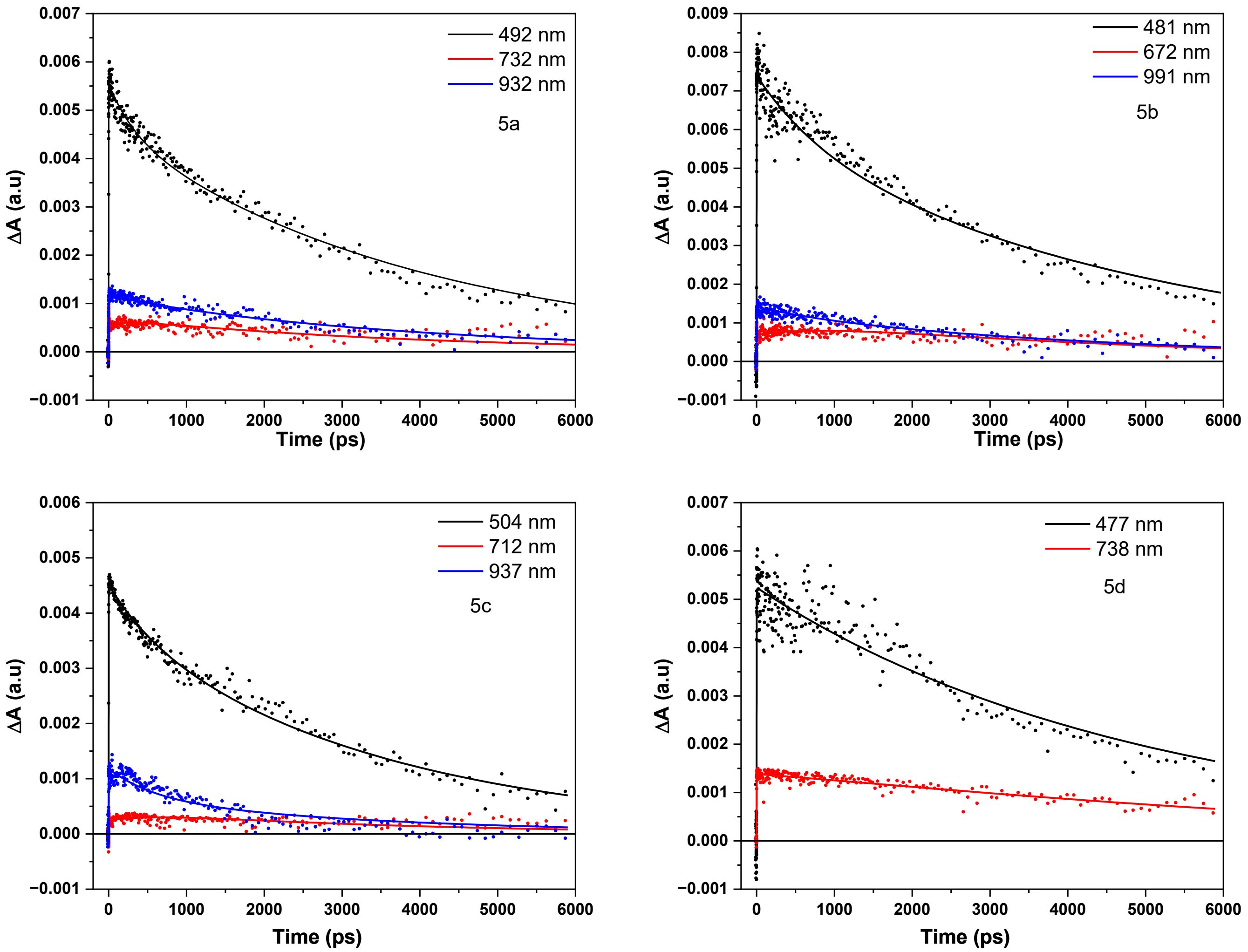

Figure S41. Comparison of the kinetics at the excited state absorption (S_1 maximum, black), and NIR spectral region (blue and red) of compounds 5a-d in toluene. The probing wavelengths for each molecule are shown in each panel. Excitation was at 420 nm. The solids lines represent fits obtained from global fitting analysis.

Table S6. Fitting parameters of compounds kinetics measured in different solvents.

λ/nm	τ_1/ps	τ_2/ps	τ_3/ps
5a acetonitrile			
490	0.17	60	1548
660	-	48.7	-
891	-	-	1837
5b acetonitrile			
479	0.21	149	2798
660	-	94	-
966	-	-	3018
5c acetonitrile			
500	1.79	96	1649
710	-	108	-
896	-	-	1353
5d acetonitrile			
470	0.14	45	3785
594	-	4	-
720	-	-	2277
5a toluene			
492	1.16	96	3568
732	-	-	2519
932	-	-	13575
5b toluene			
481	0.72	-	2894
672	-	-	-
996	0.66	-	3623
5c toluene			
504	-	-	2709
712	-	-	-
937	-	-	1321
5d toluene			
477	-	-	1800
738	-	-	non-decaying

References

1- S. Fery-Forgues and D. Lavabre, Journal of chemical education, 1999, 76, 1260.
2- Y. Zhang, S. Swaminathan, S. Tang, J. Garcia-Amorós, M. Boulina, B. Captain, J. D. Baker and F. M. Raymo, Journal of the American Chemical Society, 2015, 137, 4709-4719.
3- Valeur, B., & Berberan-Santos, M. N. (2013). Molecular fluorescence: principles and applications. John Wiley & Sons.
4- Bruker (2014) APEX2, version 2014.11-0, Bruker AXS Inc., Madison, Wisconsin, USA
5- Bruker (2013) SAINT, version V8.34A, Bruker AXS Inc., Madison, Wisconsin, USA.
6- Bruker (2014) SADABS, version 2014/4, Bruker AXS Inc., Madison, Wisconsin, USA.
7- Bruker (2010) SHELXTL, version 6.14, Bruker AXS Inc., Madison, Wisconsin, USA.
8- A. L. Spek, Biological crystallography, 2009, 65, 148-155.
9- C. F. Macrae, P. R. Edgington, P. McCabe, E. Pidcock, G. P. Shields, R. Taylor, M. Towler and J. V. D. Streek, Applied Crystallography, 2006, 39, 453-457.
10- F. Neese, WIREs Computational Molecular Science, 2018, 8, e1327.
11- A. D. Becke, The Journal of Chemical Physics, 1993, 98, 5648-5652.
12- C. Lee, W. Yang and R. G. Parr, Physical Review B, 1988, 37, 785-789.
13- B. Mennucci, WIREs Computational Molecular Science, 2012, 2, 386-404.
14- M. J. G. Peach, P. Benfield, T. Helgaker and D. J. Tozer, The Journal of Chemical Physics, 2008, 128, 044118.
15- T. Le Bahers, C. Adamo and I. Ciofini, Journal of Chemical Theory and Computation, 2011, 7, 2498-2506.
16- T. Lu and F. Chen, Journal of Computational Chemistry, 2012, 33, 580-592.
17- E. F. Pettersen, T. D. Goddard, C. C. Huang, G. S. Couch, D. M. Greenblatt, E. C. Meng and T. E. Ferrin, Journal of Computational Chemistry, 2004, 25, 1605-1612.
